# Paeoniflorin Suppresses TBHP-Induced Oxidative Stress and Apoptosis in Human Umbilical Vein Endothelial Cells *via* the Nrf2/HO-1 Signaling Pathway and Improves Skin Flap Survival

**DOI:** 10.3389/fphar.2021.735530

**Published:** 2021-11-04

**Authors:** Jingtao Jiang, Chengji Dong, Liang Zhai, Junsheng Lou, Jie Jin, Sheng Cheng, Zhuliu Chen, Xiaoshan Guo, Damu Lin, Jian Ding, Weiyang Gao

**Affiliations:** ^1^ Department of Orthopaedics, The Second Affiliated Hospital and Yuying Children’s Hospital of Wenzhou Medical University, Wenzhou, China; ^2^ Zhejiang Provincial Key Laboratory of Orthopaedics, Wenzhou, China; ^3^ The Second Clinical Medical College of Wenzhou Medical University, Wenzhou, China; ^4^ Department of Medical Cosmetology, The Second Affiliated Hospital of Xi’an Medical College, Xi’an, China

**Keywords:** paeoniflorin, random skin flap, oxidative stress, apoptosis, angiogenesis, Nrf2/HO-1 signaling pathway

## Abstract

Random-pattern skin flap is a vital technique frequently applied in reconstruction surgeries for its convenience and effectiveness in solving skin defects. However, ischemic necrosis, especially in the distal areas of the flap, still needs extra attention after surgery. Earlier evidence has suggested that paeoniflorin (PF) could stimulate angiogenesis and suppress ischemic cardiovascular disease. However, few studies have focused on the role of PF in flap survival. In this study, we have demonstrated that the human umbilical vein endothelial cells (HUVECs) treated with PF can alleviate tert-butyl hydroperoxide (TBHP)-stimulated cellular dysfunction and apoptosis. To better evaluate, HUVECs’ physiology, cell tube formation, migration, and adhesion were assessed. Mechanistically, PF protects HUVECs against apoptosis *via* stimulating the nuclear factor (erythroid-derived 2)-like 2 (Nrf2)/heme oxygenase 1 (HO-1) pathway. PF also downregulates mitochondrial ROS production to reduce excessive intracellular ROS production induced by TBHP and restore TBHP-induced mitochondrial depolarization. As a result, silencing Nrf2 partially abolishes the protective effect of PF exposure on HUVECs. In *in vivo* experiments, the oral administration of PF was shown to have enhanced the vascularization of regenerated tissues and promote flap survival. However, the PF-mediated protection was partially lost after co-treatment with ML385, a selective Nrf2 inhibitor, suggesting that PF is a crucial modulator regulating the Nrf2/HO-1 signaling pathway. In summary, our data have provided a new insight into PF as a potential therapy for enhancing random-pattern flap viability.

## Introduction

The random-pattern skin flap is a crucial tool for tissue reconstruction due to its simplicity, reliability, and convenience (Chen et al., 2021; Luo et al., 2021), which is particularly popular in plastic and reconstructive surgeries (Zhou et al., 2019). The microvascular network at the junction of the skin flap and tissue usually supports the survival of the skin flap with nutrition, which is prone to ischemia and necrosis due to insufficient blood supply at distal flaps (Wang et al., 2017). In particular, when the length–width ratio of the flap is greater than 2:1, it often leads to ischemia necrosis (Li J. et al., 2021). This complication significantly limits its clinical application and postoperative efficacy (Chehelcheraghi et al., 2016). Hence, seeking novel approaches to enhance the survival of the random-pattern skin flap is of great clinical significance.

Randomized model flap survival involves multicellular processes, including keratinocytes, fibroblasts, endothelial cells, macrophages, and platelets (Barrientos et al., 2008). Angiogenesis is thought to play a vital role in the survival of the distal flap, which is mainly performed by endothelial cells (Li S. et al., 2021). In fact, the microvascular network containing angiogenesis capillaries provides nutrient and oxygen to distal flaps and promotes their viability during the healing process of posttransplantation (Tonnesen et al., 2000). Previous studies have reported that the activation of Nrf2/HO-1/HIF-1α axis could upregulate the VEGF expression (Li et al., 2016; Huang et al., 2018). Unfortunately, oxidative stress is generated at the injury site under the conditions of oxidative phosphorylation, which is detrimental to endothelial cells (Huang K. et al., 2021). Hence, protecting endothelial cells against oxidative stress is crucial and urgent for the skin flap viability. In addition, attenuating the ischemic reperfusion injury of ischemic tissue during the blood flow recanalization also enhances the survival of skin flaps.

Nrf2 is an important redox-sensitive transcription factor which promotes cell viability and maintains redox homeostasis by inducing and regulating the constitutive and inductive expressions of phase II detoxifying enzymes and antioxidant enzymes in cells, including heme oxygenase-1 (HO-1) (Li et al., 2009). Hemeoxygenase-1 (HO-1) is an important antioxidant enzyme that catalyzes the metabolization of heme into ferrous, carbon monoxide, and biliverdin (Kim et al., 2018). Under normal physiological conditions, Nrf2 is anchored by Keap1 in the cytoplasm, and Keap1, which promotes Nrf2 ubiquitination and is rapidly degraded by proteasomes, is considered as a substrate for the Cullin 3 (Cul3)-dependent E3 ubiquitin ligase complex. However, when cells are attacked by ROS or electrophiles, Nrf2 would be dissociated from Keap1 and rapidly translocate into the nucleus, forming a heterodimer with small Maf proteins and binding antioxidant response elements (ARE) (Ho et al., 2012). In previous studies, the Nrf2-activating compound N-Me-trichodermamide B isolated from *Penicillium janthinellum* (Yang et al., 2017) protected HUVECs from UV and TBHP-induced apoptosis by inhibiting ROS production while increasing HO-1. To sum up, Nrf2/HO-1 signaling is considered to have cytoprotective effect against ROS-induced endothelial cell injury and apoptosis.

After oxidative stress induces cell death signals, intracellular proapoptotic proteins undergo posttranslational modifications such as acetylation and splicing, and then cytochrome C is released from the apoptotic mitochondria (Huang Y. et al., 2021). At this level, Bcl-2 family proteins are the main regulators of apoptosis, and the expression levels of Bcl-2 and Bax are relatively stable. Once the ratio between the two opposing proteins is broken, cytochrome C will be released from the mitochondria (Veerasamy et al., 2021). Cytochrome C enters the cytoplasm from mitochondria, where it forms an apoptotic activation complex with apoptotic protease activating factor 1 (Apaf-1) in the cytoplasm, activating caspase-9 and the downstream caspase-3 and other caspases, leading to apoptosis (Bao et al., 2021). It is worth noting that mitochondrial ROS (mtROS) stimulates the release of cytochrome C from mitochondria to cytoplasm by disrupting the balance of the system, such as mitochondrial membrane potential downregulation and mitochondrial glutathione oxidation (López-Pérez et al., 2021), suggesting that downregulation of mtROS protects HUVECs from UV-induced apoptosis and exogenous injury such as ROS. Olguín-Albuerne and Morán et al. (2018) demonstrated that Nrf2 was involved in the production of both cytoplasm and mtROS through nicotinamide adenine dinucleotide phosphooxidase, indicating that Nrf2 could downregulate ROS-mediated cytoplasmic and mitochondrial apoptosis.

When the blood flow of ischemic tissue is restored in the recovery process of the flap injury, oxygen molecules brought by blood can easily react with the cell membrane to perform and also destroy lipid peroxidation, resulting in cell death and tissue necrosis. Therefore, oxidative stress is a key determinant of tissue survival (Zhou et al., 2019; Sun et al., 2021). TBHP can stably cause oxidative stress in endothelial cells (Lin et al., 2019); thus, it was used as an *in vitro* stimulus to simulate the oxidative stress process in the survival of the skin flaps. Paeoniflorin (PF), derived from the root of *Paeonia albiflora* Pall, is a traditional Chinese herbal substance (Zhao et al., 2021), which is also well known for its antibacterial, antiviral, antioxidant, and other pharmacological properties (Huang L. et al., 2021). Additionally, previous studies have reported that PF could exert antioxidant effects through the Nrf2 signaling pathway (Lu et al., 2020; Dong et al., 2021). Paeoniflorin activates antioxidant enzymes such as SOD, CAT, and GPX (Jia and He, 2016; Tu et al., 2019). However, studies on the effect of PF on flap survival are scarce. Herein, we examined the PF antioxidant property on human umbilical vein endothelial cells (HUVECs) treated with tert-butyl hydroperoxide (TBHP) treatment and explored its underlying mechanism. Additionally, the random pattern skin flap model was constructed to evaluate whether PF could promote the flaps survival in *in vivo* test models.

## Methods

### Reagents and Antibodies

Paeoniflorin (purity: 98.04%) was acquired from MedChemExpress (Monmouth Junction, NJ, United States). Tin protoporphyrin IX (SnPP) and dimethyl sulfoxide (DMSO) were obtained from Sigma-Aldrich (St. Louis, MO, United States). Primary antibodies were used as following: C-caspase3 (ab32351), Bax (ab32503), Bcl-2 (ab182858), and Cytochrome C (ab133504) were obtained from Abcam (Cambridge, United Kingdom); Keap1 (sc-514914) and poly (ADP-ribose) polymerase (PARP) (sc-7150) were purchased from Santa Cruz Biotechnology (Santa Cruz, CA, United States). Primary antibodies against Nrf2 (16396-1-AP), HO-1 (27282-1-AP), Lamin B (12987-1-AP), NF-κB (66535-1-Ig) and β-actin (66009-1-Ig) were acquired from Proteintech Group (Chicago, IL, United States), and 4′, 6-diamidino-2-phenylindole (DAPI) was received from Beyotime (Shanghai, China). ML385 was purchased from ChemeGen (Shanghai, China). All experimental consumables for cell culture are from Gibco (Grand Island, NY, United States).

### HUVEC Culture and Treatment

HUVECs were acquired from ATCC (Manassas, VA, United States) and cultured in DMEM/F12 (Gibco, Invitrogen, Grand Island, NY) with 10% heat-inactivated FBS, and 1% penicillin and streptomycin were added to a 5% CO_2_ incubator at 37°C. To elucidate the role of PF in HUVEC survival, cells were exposed to differing PF (0, 5, 10, 20, and 50 μM) concentrations over 24 h. To induce oxidative stress and apoptosis *in vitro*, cells were cultured with a range of concentrations of TBHP (0, 100, 200, 500, and 1000 μM), and cell toxicity was measured. After determining the optimal TBHP concentration, HUVECs were exposed to different concentrations of PF (0, 5, 10, and 20 μM) and TBHP (500 μM), and worked together for 24 h to detect the PF-mediated apoptosis and deregulation protection. To delineate the function of HO-1 in this process, HUVECs were pretreated with an HO-1 inhibitor SnPP (20 μM) for 2 h before PF exposure. The data presented here represent replicas of three separate experiments.

### Cytotoxicity Assay

HUVEC viability were evaluated with the Cell Counting Kit-8 (CCK-8) assay (MedChemExpress LLC; Monmouth Junction, NJ, United States), following operational guidelines. In brief, second-generation HUVECs were cultured in 96-well plates (3 × 10^3^ cell/well) and exposed to a combination of differing PF (0, 5, 10, and 20 μM) concentrations and TBHP (500 μM), before incubation in DMEM/F12 medium at 37°C. After 24 h, the cells were PBS-rinsed and exposed to 10 μl CCK8 in DMEM/F12 serum-free medium for an additional 2 h. Finally, absorbance was detected at 450 nm with a microwell plate reader (Thermo Fisher, Waltham, MA, United States).

### Mitochondria and Cytosol Isolation

Isolation of cytosol and mitochondria was performed according to the instructions for the Mitochondria/Cytosol Isolation Kit (Applygen Technologies, Beijing, China) (Zhang et al., 2014). Fifty million HUVECs were resuspended with ice-cold mito-cyto isolation buffer (Applygen Technologies Beijing, China) and homogenized. The homogenate was centrifuged at 800 × g for 10 min at 4°C. The supernatants were combined in a new tube and centrifuged at 12,000 × g for 10 min at 4°C. The supernatant contained the cytosolic fraction and the pellet contained intact mitochondria.

### Western Blotting

Western blot was conducted, as described before (Lin et al., 2018). In brief, HUVECs treated with differing PF (0, 5, 10, and 20 μM) concentrations and TBHP (500 μM) were lysed in 1 mM PMSF (phenylmethanesulfonyl fluoride) radioimmunoprecipitation analysis buffer to extract protein and were subsequently subjected to protein quantification with the BCA Protein Assay Kit (cat# P0012S, Beyotime, Shanghai, China). The equivalent amount of proteins (30 µg per sample) were separated by gel electrophoresis and transferred to the PVDF membrane (BioRad, United States). Next, the membranes were blocked in 5% skim milk at room temperature for 2 h, exposed to primary antibodies at 4°C for 18 h: cleaved caspase-3 (C-caspase3) (1:1,000), Bax (1:1,000), Bcl-2 (1:1,000), β-actin (1:1,000), Cytochrome C (1:1,000), Nrf2 (1:1,000), HO-1 (1:1,000), or Lamin B (1:800), followed by washing and exposure to corresponding HRP-conjugated secondary antibodies for 2 h at room temperature. The strips were imaged using a chemical XRS + imaging system (Bio-Rad, United States). The final protein quantification was done with Image LabV 3.0 (Bio-Rad, United States).

### Tunel Assay

Apoptotic cells were detected with the *in situ* Cell Death Detection Kit (Roche, South San Francisco, CA, United States), following operational guidelines. In brief, HUVECs were treated with different concentrations of PF (0–20 μM) for 24 h with or without 500 μM TBHP for 2 h. The treated HUVECs were PBS-rinsed thrice and fixed in 4% paraformaldehyde for 20 min at room temperature, followed by incubation with 3% hydrogen peroxide and freshly made 0.1% triton X-100 for 10 min. At every step, the cells were PBS-rinsed thrice. We used the TUNEL reagent to detect the cells, and the amount of apoptotic cells were calculated from three arbitrary fields, using a fluorescence microscope (Olympus Inc., Tokyo, Japan).

### Tube Formation Assay

The chamber glass slide was first prepared with endothelial cell matrix gel solution (BD Biocoat, NJ, United States) and kept in a humidified environment at 37°C for 1 h to facilitate matrix solidification. HUVECs were treated with different PF concentrations (0, 5, 10, and 20 μM) for 24 h with or without 500 μM TBHP for 2 h. Pretreated HUVECs were next harvested with trypsin/ethylenediaminetetraacetic acid, plated onto the already made matrigel-coated glass slides, and maintained at 37°C for 6 h. Tube generation was detected with a phase contrast microscope (Olympus Inc., Tokyo, Japan) and counted using randomly selected areas within each well.

### Cell Migration Assay

HUVEC migration was evaluated using an 8 μm polycarbonate membrane Boyden chamber inserted in a transwell system (CoStar, Cambridge, MA, United States). HUVECs were treated with different concentrations of PF (0–20 μM) for 24 h with or without 500 μM TBHP for 2 h, as mentioned previously. Next, HUVECs were separated, centrifuged, and resuspended before 4 × 10^4^ cells, from each treatment, were plated into the transwell system in 400 μl of non-FBS DMEM/F12 medium. Furthermore, 600 μl of medium with 1% FBS was introduced to the bottom chamber. After a 12 h incubation in 5% CO_2_ humidified environment, the membranes were PBS-washed thrice, and the cells were fixed with 4% paraformaldehyde. Additionally, any cells on top of the transwell system were removed with a cotton swab, and it was stained with crystal violet. Migratory cells that traveled from the top to the lower surface of the insert were calculated in three arbitrary fields, using an inverted microscope (Olympus Inc., Tokyo, Japan).

### Small Interfering Ribonucleic Acid Transfection

Double-stranded siRNA was designed and synthesized for silencing human Nrf2 gene (RiboBio, Guangzhou, China). The sequence is listed below: sense strand 5′-CGT​CAT​TGA​TGA​TGA​GGC​T-3′. HUVECs were incorporated with 50 nM siRNA and Lipofectamine 2000 reagent (Thermo Fisher, UT, United States) for 36 h, following operational guidelines. Subsequently, the cells were exposed to different concentrations of PF (0–20 μM) for 24 h with or without 500 μM TBHP for 2 h, as described before. Finally, relevant protein levels were assessed with Western blot.

### Intracellular ROS Assay

The production of intracellular ROS was determined by oxidation sensitive dye (DCFDA). HUVECs were inoculated at a density of 1 × 10^4^ cell/ml and treated with a specified concentration of PF (0–20 μM) for 24 h, following by 500 μM TBHP for 2 h. The cells were rinsed with PBS and immediately treated with 10 μM DCFDA. Intracellular ROS generation was observed by fluorescence microscope (Olympus Inc., Tokyo, Japan).

### Measurement of SOD, CAT, and GPX Activities and Intracellular ATP Assay

HUVECs were treated with different concentrations of PF (0, 5, 10, and 20 μM) for 24 h with or without 500 μM TBHP for 2 h. Then treated HUVECs were washed with PBS three times and placed on ice for 15 min. SOD (S0086, Beyotime, China), CAT (S0082, Beyotime, China), and GPX (S0058, Beyotime, China) assay kits were used to detect the expression levels. We used the ATP Assay Kit (S0026, Beyotime, China) to assess the mitochondrial energy production in HUVECs.

### Mitochondrial Function Determination

HUVECs were treated with different concentrations of PF (0, 5, 10, and 20 μM) for 24 h with or without 500 μM TBHP for 2 h. The treated HUVECs were used with JC-1 staining to determine the changes of mitochondrial membrane potential (MMP). The level of mitochondrial ROS production and mitochondrial distribution was detected by MitoSox staining and MitoTracker (C1035-250μg, Beyotime, China), according to the vendors’ instructions. Fluorescence microscope (Olympus, Tokyo, Japan) was applied to capture the images.

### Animals Experiment

Seventy-two adult male Sprague–Dawley rats (250–300 g) were purchased from Wenzhou Medical University (license no. SYXK [ZJ] 2020-0014) and maintained at 23 ± 2°C with 50 ± 5% humidity, and a 12 h light/dark cycle. Additionally, all the animal experimental protocols were agreed upon by the Wenzhou Medical University’s Animal Research Committee (wydw 2021-0256). The rats were randomly assigned to four experimental categories, namely, control (*n* = 18), PF (*n* = 18), ML385 (*n* = 18), and PF+ML385 (*n* = 18).

### Skin Flap Model Establishment and Drug Administration

All rats were anaesthetized with 2% (w/v) sodium pentobarbital (10 mg/kg) *via* oral administration. Random-pattern skin flaps were created from the central dorsum, using the modified McFarlane flap technique (Lin et al., 2018). In short, an electric razor and hair removal cream were used to eliminate back hair. Next, a caudal skin flap (3 cm × 9 cm) was excised from the subcutaneous deep fascia of the rat’s back. Following this, the bilateral exposed iliac arteries were cut and sectioned. Next, the flap was placed on the donor bed and immediately sutured using 4-0 non-absorbable sutures. The flap on the back can be divided into three equal and independent areas: zone I (near the tail of the flap), zone II (middle region), and zone III (distal region). The PF group animals were administered 10 mg/kg PF *via* daily oral administration for 7 days after the establishment of the random-pattern skin flaps (Dong et al., 2020). Animals in the control group were given the same amount of saline (p.o 10 mg/kg, daily). The ML385 group received 30 mg/kg ML385 daily dose. Last, the PF+ML385 animals received 10 mg/kg PF and 30 mg/kg ML385 daily dose, similar to the other groups. After seven consecutive days of treatment, the rats were sacrificed with excess anesthesia, and tissue samples (1 cm × 1 cm) were collected from the zone II flaps for further study.

### Flap Survival Evaluation

Macroscopic properties, such as appearance, color, and hair condition of the flap, were observed and recorded on day 7 after the operation. On the 7th day, the percentage of the flap survival area was assessed using ImageJ software (ver. 6.2; Media Cybemetics). The survival area (in %) was obtained as listed below: % survival = survival area ÷ total area × 100%.

### Laser Doppler Blood Flow Imaging

To examine vascularization under the flap, rats were anesthetized and made to lie prone, while vascularization was recorded with a laser dopplerometer on postoperative day 7 (POD 7). The data obtained were analyzed with moor LDI review (ver.6.2; Moor Instruments), and perfusion units (PU) were used to assess the blood flow intensity. Scans were performed thrice, and the mean of the results was used to search for statistical significance.

### Histological Analysis

Six tissue specimens (1 cm × 1 cm) were harvested per group from area II to conduct hematoxylin and eosin (H&E) staining. These specimens were fixed in 4% (v/v) paraformaldehyde for 1 day, embedded in paraffin wax before being cut into 4 µm thick sections, and stained with H&E. The sections were observed under an optical microscope (Leica, Wetzlar, Germany). Microvessels were measured on six arbitrary fields per section, and the microvessel quantity per unit area (/mm^2^) was counted to assess the level of microcirculation.

### Immunohistochemistry

Rat skin tissues obtained from area II of the flaps were fixed in 4% paraformaldehyde. Following deparaffinization of the tissue sections in xylene, the sections were rehydrated *via* graded ethanol bath. Next, the samples were PBS-washed thrice, blocked with 3% hydrogen peroxide, and repaired in 10.2 mM sodium citrate buffer before an overnight exposure with following primary antibodies: CD34 (1:100), Nrf2 (1:100), VEGF (1:100), HO-1 (1:100), NF-κB (1:100), Bax (1:100), and C-caspase3 (1:100). Subsequently, tissue samples underwent exposure to HRP-conjugated secondary antibody, followed by staining with the DAB kit, and counterstained with hematoxylin, before visualization under a light microscopy (200× magnification). Quantified absorption values were estimated *via* Image-Pro Plus to detect CD34, Nrf2, HO-1, Bax, NF-κB, VEGF, and C-caspase3 expression levels. Six randomly chosen fields from six sections were analyzed for immunohistochemistry (IHC) statistical calculation.

### Immunofluorescence

For cell immunofluorescence, HUVECs were grown in a 6-well plate for 24 h exposed to PF (20 μM) for 24 h, subsequently, the cells were again exposed 500 μM TBHP for 2 h. Next, the cells were PBS-rinsed, fixed with 4% paraformaldehyde for 15 min, permeabilized with 0.1% TritonX-100 in PBS for 10 min, blocked with 5% bovine serum albumin (BSA) for 1 h at room temperature, with subsequent overnight exposure at 4°C to primary antibodies against Nrf2 (1:200; Abcam), followed by exposure to Alexa Fluor^®^488- or Alexa Fluor^®^594 goat anti-rabbit IgG (H+L) secondary antibodies (1:300; Jackson Immunoresearch, PA, United States) for 1 h at 37°C before staining with DAPI (Beyotime, China) for 5 min, and finally observed under a Nikon ECLIPSE Ti microscope (Nikon, Japan). Six arbitrary fields were analyzed for each slide. Fluorescence quantification was done with Image-Pro Plus (Media Cybernetics, Rockville, MD, United States).

For tissue immunofluorescence, the tissue specimen was deparaffinized, rehydrated, and PBS-washed in the initial steps of immunofluorescence preparation. Meanwhile, the endogenous peroxidase was quenched with 3% hydrogen peroxide, and tissue antigen was repaired using 10.2 mM sodium citrate buffer. The slides were then incubated with primary antibody against a-SMA overnight at 4°C. After washing with PBS, the slices were incubated with Texas red-conjugated anti-IgG secondary antibody and stained by DAPI. Visualization was done with a fluorescence microscope (Zeiss, LSM800, Germany).

### Real-Time PCR

Total RNA was extracted from the skin flaps using TRIzol reagent according to the manufacturer’s specifications. Quantification was performed using reverse transcription (RT) and PCR. RT reaction containing 0.5 μg of RNA, 2 μl of 5× TransScript all-in-one SuperMix for qPCR, and 0.5 μl of gDNA remover in a total volume of 10 μl. The reactions were performed in a GeneAmp^®^9700 PCR system (Applied Biosystems, United States) for 15 min at 42°C and 5°s at 85°C. The RT reaction mix was diluted in nuclease-free water and stored at −20°C. The real-time PCR was performed using a LightCycler^®^480 II real-time PCR instrument (Roche, Switzerland) in 10 μl PCR mixture containing 1 μl of cDNA, 5 μl of 2× PerfectStartTM Green qPCR SuperMix, 0.2 μl of forward primer, 0.2 μl of reverse primer, and 3.6 μl of nuclease-free water. The samples were incubated in a 384-well optical plate (Roche, Switzerland) at 94°C for 30°s, followed by 45 cycles of 94°C for 5°s and 60°C for 30°s. Each sample was assayed in triplicate. At the end of PCR, melting curve analysis was performed to validate specific generation of expected PCR products. The primer sequences were designed in the laboratory and synthesized by TsingKe Biotech based on the mRNA sequences obtained from the NCBI database as follows: *Nrf2, 5*′*- GCA​TTT​CGC​TGA​ACA​CAA-3*′ *(forward), and 5*′*-CTC​TTC​CAT​TTC​CGA​GTC​A-3*′ *(reverse); H O -1, 5*′*-AGA​GTT​TCT​TCG​CCA​GAG​G-3*′ *(forward), and 5*′*-GAG​TGT​GAG​GAC​CCA​TCG-3*′ *(reverse); Bax, 5*′*-CGG​CTG​CTT​GTC​TGG​AT-3*′ *(forward), and 5*′*- TGG​TGA​GTG​AGG​CAG​TGA​G -3*′ *(reverse); Bcl-2, 5*′*-CGG​GAG​AAC​AGG​GTA​TGA-3*′ *(forward), and 5*′*- AGG​CTG​GAA​GGA​GAA​GAT​G-3*′ *(reverse);* glyceraldehyde 3-phosphate dehydrogenase (GAPDH) expression was used as an internal control using the 2^−ΔΔCt^ method to evaluate the expression level of the target gene.

### Molecular Modeling

Nrf2 (PDB ID: 4ZY3) was subjected to docking studies (Khan et al., 2017). The protein was downloaded from PDB (https://www.rcsb.org/) before being prepared for docking. The lowest energy conformations for docking were determined *via* default parameters after being minimized using PYMOL 2.3.4. The protein–ligand docking analysis was carried out using AutoDock Vina 1.1.2, which can provide ligand binding flexibility with the binding pocket residues. Finally, the images were generated using UCSF PyMoL.

### Statistical Analysis

Data are represented by the means ± standard deviation. All statistical analyses were performed by one-way analysis of variance (ANOVA) with Tukey’s multiple comparisons test to assess for differences among the groups. *p* value < 0.05 was regarded as statistically significant.

## Results

### Paeoniflorin Alleviated Apoptosis in HUVECs

**FIGURE 1:**
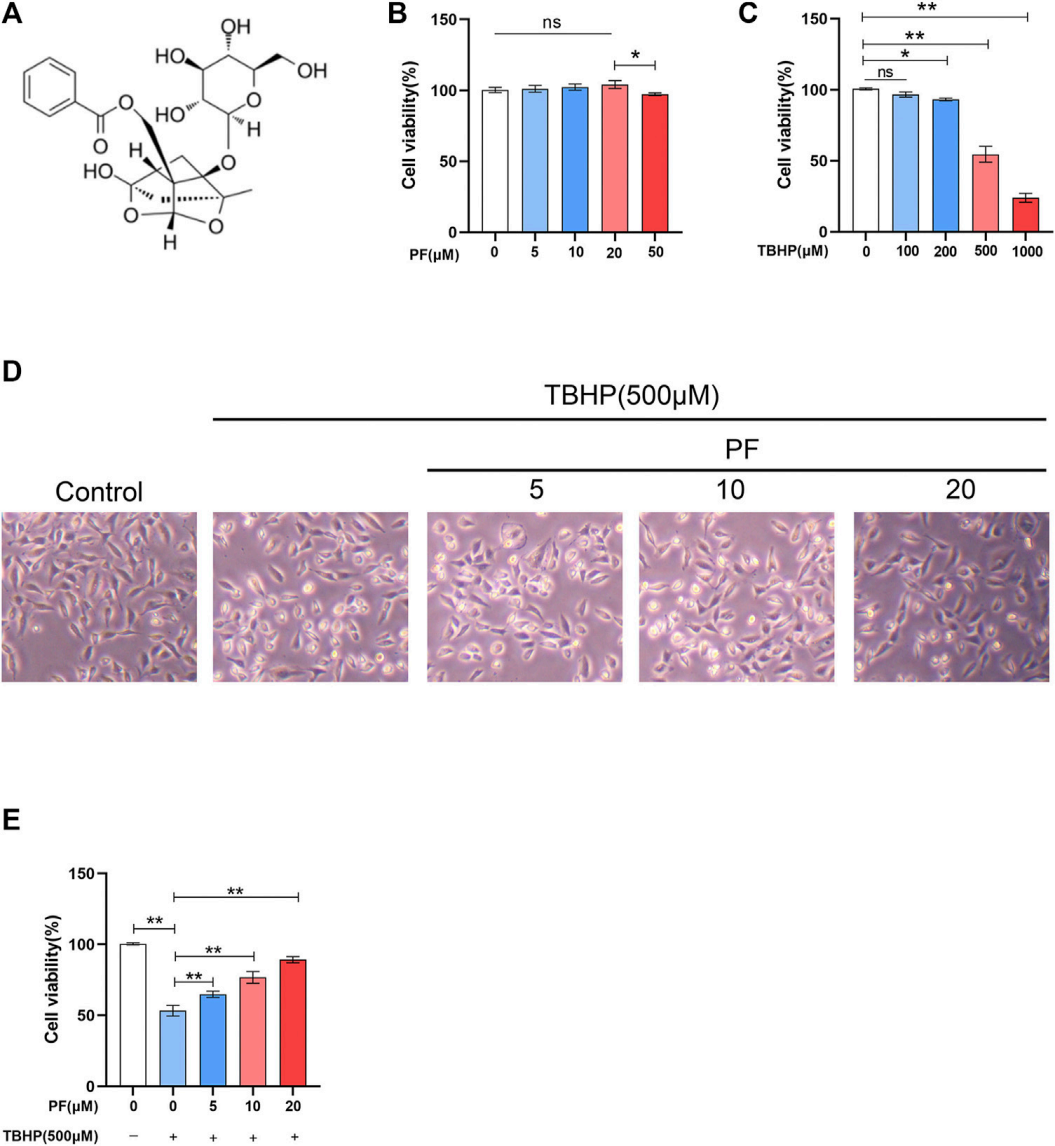
Effect of paeoniflorin (PF) on HUVECs. **(A)** PF chemical structure. **(B)** Evaluation of HUVEC viability using CCK-8 assay after exposure to differing concentrations of PF for 24 h. **(C)** Evaluation of HUVEC viability using CCK-8 assay after stimulation to different concentrations of TBHP for 24 h. **(D)** Representative images demonstrating cell morphology alterations in PF and TBHP co-treatment in HUVECs (scan bar, 100 μm). **(E)** Evaluation of HUVEC viability using CCK-8 assay after PF and TBHP co-treatment in HUVECs. Data: mean ± SD, *p < 0.05 and **p < 0.01. *n* = 3.

**FIGURE 2:**
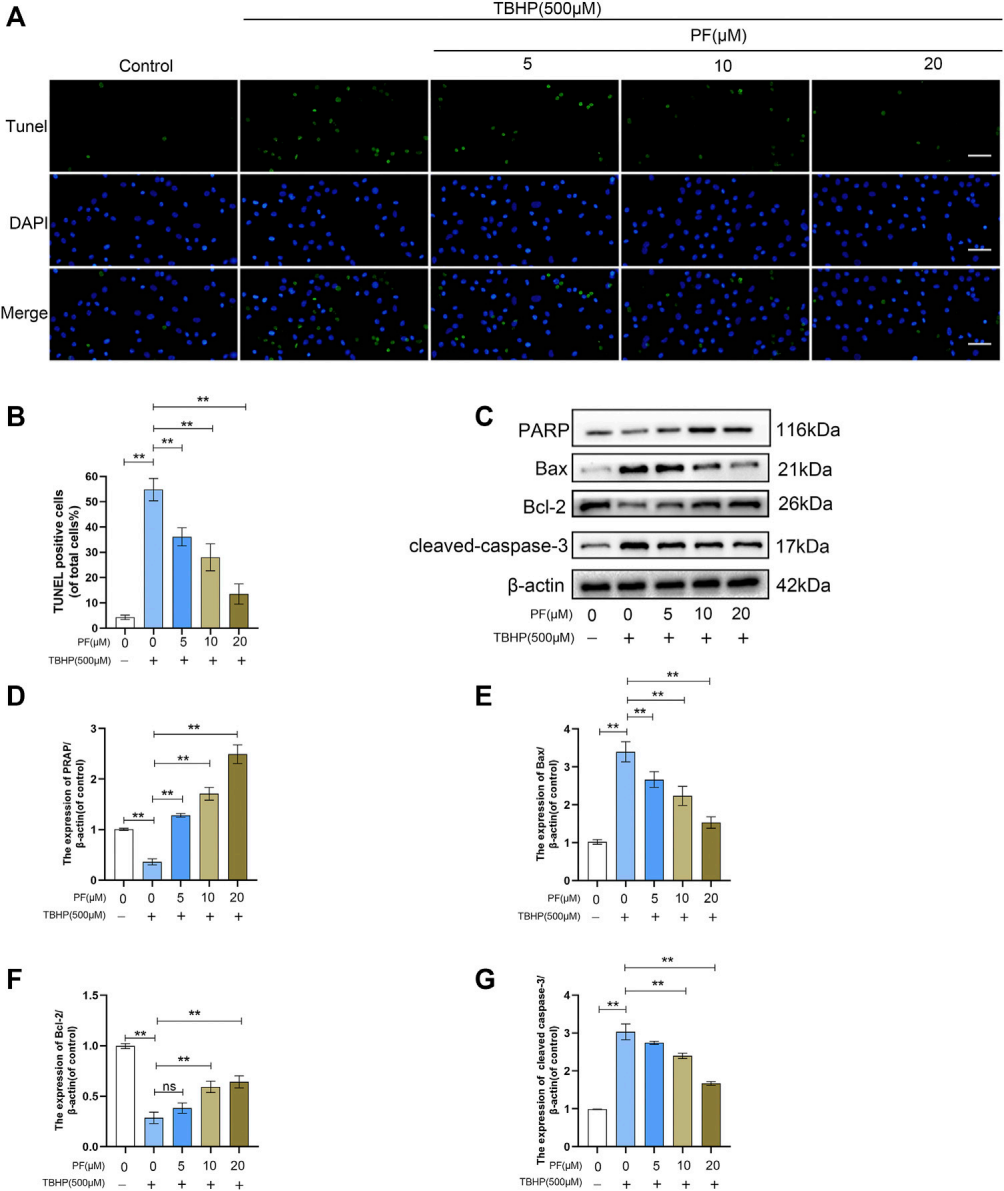
Paeoniflorin (PF) alleviated TBHP-induced apoptosis in HUVECs. **(A)** Evaluation of DNA damage (scan bar, 100 μm) (nuclei, blue; positive cells, green) using TUNEL assay. **(B)** Histogram shows the proportion of TUNEL positive cells. **(C–G)** Evaluation of poly (ADP-ribose) polymerase (PARP), Bax, Bcl-2, and cleaved caspase-3 levels in HUVECs under different treatment conditions using Western blotting. Data: mean ± SD, *p < 0.05 and **p < 0.01. *n* = 3.

To estimate whether PF exerts cytotoxic effects on HUVECs, HUVECs were treated with various doses of PF (Figure 1B). Damaged cells were not observed below 20 μM PF in HUVECs. Based on this, 20 μM PF was determined as the highest nontoxic concentration for subsequent experiments. To assess the optimal concentration of TBHP as a suitable exogenous oxidative stimulus source, the HUVECs were then treated with TBHP concentration gradient (0–1,000 μM), and subsequently, the cell activity was detected. With the increasing of TBHP concentration, the cell morphology atrophy gradually increased, and apoptotic bodies were visible. Compared with unprocessed group, the cell viability was significantly decreased to 54.6 ± 4.5% at 500 μM TBHP (Figure 1C). Subsequently, PF demonstrated a protective effect on TBHP-treated cell damage in a dose-dependent manner (64.9 ± 1.8%, 76.8 ± 3.4%, 89.3 ± 1.9%, respectively, at 5, 10, and 20 μM PF, comparing with that of TBHP-treated group), indicating that PF had exerted cytoprotective effect on TBHP-mediated cytotoxicity (Figures 1D,E). Additionally, TUNEL were introduced for the assessment of PF-induced protection of TBHP-stimulated HUVECs. As expected, the amount of TUNEL+ cells was upregulated with exposure to TBHP but alleviated with PF in a concentration-dependent manner (35.8 ± 1.7%, 28.3 ± 0.8%, 11.8 ± 3.7%, respectively, at 5, 10, and 20 μM PF, comparing with that of TBHP-treated group (51.5 ± 3.4%) (Figures 2A,B). To assess whether the cytoprotective effect of PF was attributable to antioxidant activity, the expression of antiapoptotic proteins such as PARP and Bcl-2 was measured, and proapoptotic proteins such as Bax and cleaved caspase-3 were calculated in HUVECs treated with or without TBHP. TBHP significantly downregulated PARP and Bcl-2, which was shown in Figure 2C. However, PF restored these protein levels in a dose-dependent manner compared with the TBHP-induced group, especially at 20 µM. PF also significantly increased the levels of PARP and Bcl-2 compared with the TBHP-administered group, suggesting that PF could potentially promote the expression of antiapoptotic proteins in HUVECs treated with TBHP (Figures 2D,F). As envisioned, compared with the unadministered group, TBHP had induced massive expression of Bax and cleaved caspase-3; furthermore, these effects were reversed in the PF-treated group, indicating that PF inhibited the expression of Bax and the cleavage of caspase-3 in TBHP-induced oxidative stress (Figures 2E,G). In conclusion, these results suggested that PF had protected HUVECs from apoptosis in response to TBHP-mediated oxidative stress.

### Paeoniflorin Ameliorated Mitochondrial Functional Damage and mtROS Production in TBHP-Induced HUVECs

**FIGURE 3:**
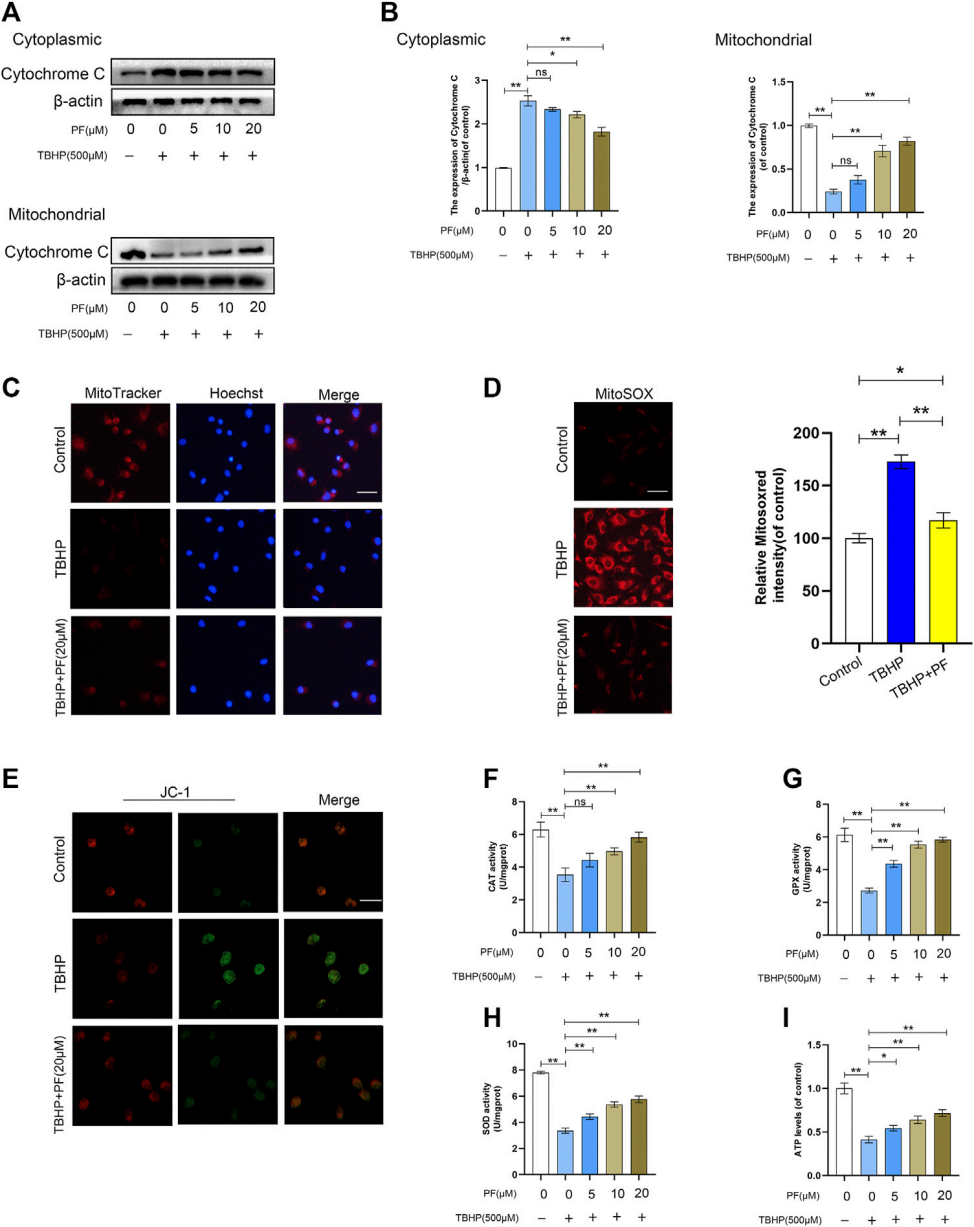
Paeoniflorin (PF) ameliorated TBHP-induced oxidative stress and mitochondrial functional damage in HUVECs. **(A)** Western blotting showing levels of cytoplasmic and mitochondrial cytochrome C under different treatment conditions. **(B)** Histogram showing quantificational comparison of cytoplasmic and mitochondrial cytochrome C expressions in the PF (0–20 μM) with or without TBHP (500 μM) groups. **(C)** The cells were stained with 0.5 µM MitoTracker for 30 min, and nuclei were stained with Hoechest (blue). Immunofluorescence staining was performed and detected by using the Nikon ECLIPSE Ti microscope (Nikon, Japan). (bar: 20 μm). **(D)** The cells were stained with 2 µM MitoSOX Red for 10 min and fluorescence intensity was measured using a fluorometer. Percentage values were calculated compared to those in the untreated group. (bar: 20 μm). **(E)** JC-1 staining was performed to detect the mitochondrial membrane potential (MMP) (bar: 20 μm). **(F–G)** Activities of CAT, GPx, and SOD in the differentially treated HUVECs. **(H)** Activity of ATP in different treatment groups. Data: mean ± SD, *p < 0.05 and **p < 0.01. *n* = 3.

Then, the effect of PF on TBHP-induced mitochondrial depolarization and mtROS generation was investigated. To assess the expression of related proteins in mitochondrial dysfunction, the expression of cytochrome C was measured. The Western blotting results had revealed that cytoplasmic cytochrome C was significantly increased by PF in a concentration-dependent manner, while the level of mitochondrial cytochrome C had decreased (Figures 3A,B). Next, the mitochondrial functional damage and mtROS production in TBHP-treated HUVECs were explored. The high expression of mtROS and mitochondrial dysfunction severely affected the HUVECs functional state. MitoSox red staining revealed that compared with the untreated group, the TBHP-treated group resulted in 172.7 ± 5.2% of mtROS. Then, compared with untreated group, the effect at 20 µM of PF co-treated with TBHP (500 µM) had nearly restored the mtROS generation (Figure 3D). Consistent with the MitoSox data, it was also found that fluorescence images of MitoTracker and JC-1 had largely restored the mitochondrial membrane potential decreased by TBHP. Immunofluorescence staining also revealed that treatment with TBHP significantly increased mtROS (MitoSOXRed) generation in the mitochondria (MitoTracker); however, PF had completely suppressed TBHP-mediated mtROS generation (Figures 3C,E). In addition, TBHP-treated HUVECs, co-cultured with PF, also restored the activities of SOD, CAT, and GPx (Figures 3F–H), along with ATP levels (Figure 3I). In conclusion, these results have suggested that PF downregulated the intracellular ROS generation in TBHP-induced HUVECs by stabilizing the MMP and subsequently inhibiting oxidative stress–mediated apoptosis.

### Paeoniflorin Promoted Cell Function in TBHP-Treated HUVECs

**FIGURE 4:**
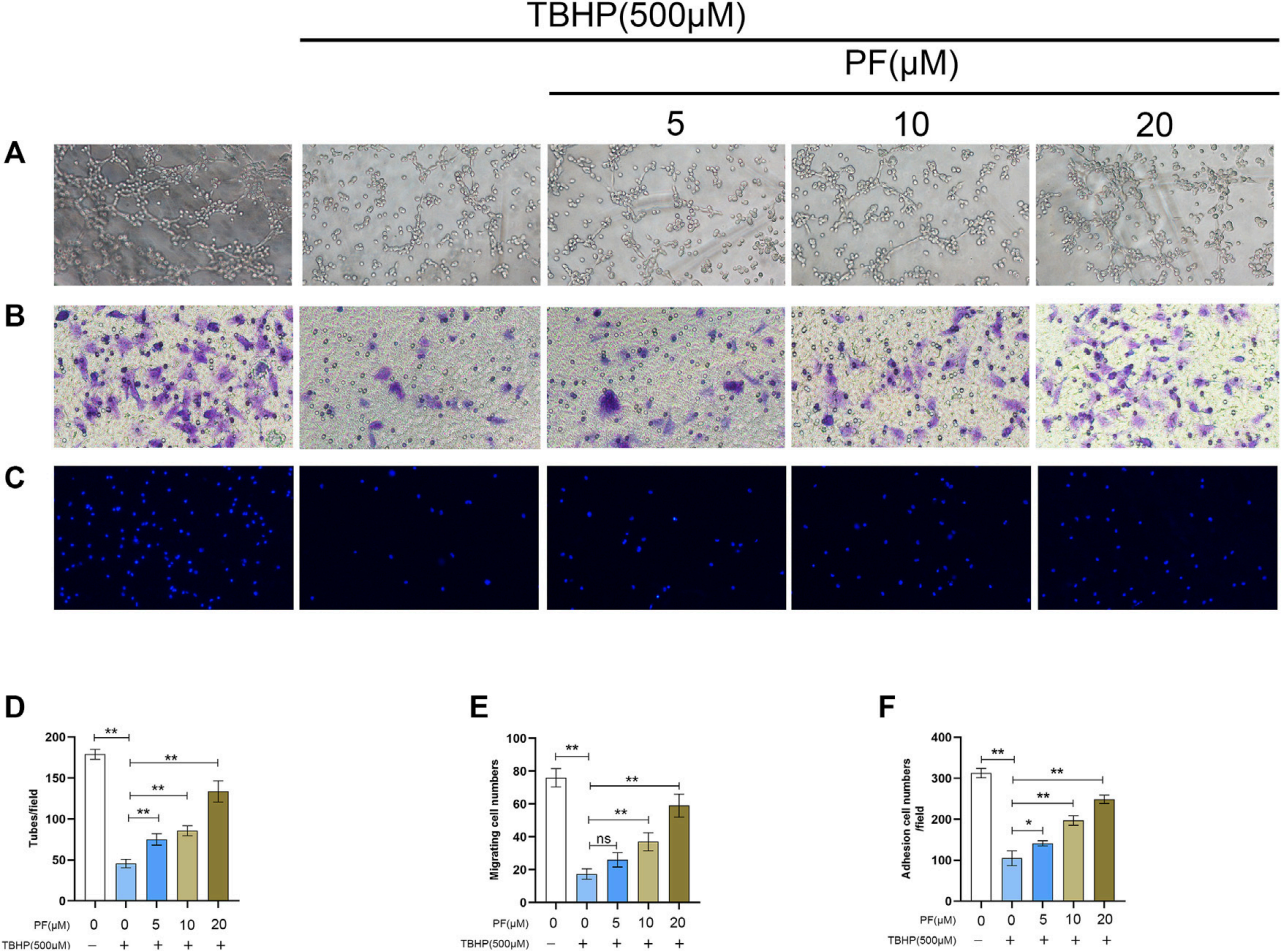
Paeoniflorin (PF) promoted cell function in TBHP-treated HUVECs. **(A,D)** Evaluation of PF-mediated HUVEC neovascularization using tube formation assay (scale bar, 100 μm). **(B,E)** Assessment of PF-mediated HUVEC migration using the transwell system (scale bar, 100 μm). **(C,F)** Evaluation of PF-mediated HUVEC adhesion using cell-matrix adhesion assay (scale bar, 100 μm). Data: mean ± SD, *p < 0.05 and **p < 0.01. *n* = 3.

To determine the therapeutic benefit of PF on cellular function, neovascularization evaluation was first performed *via* the tube formation assay, and the number of endothelial cell tubules in each group was counted. Based on a dose-dependent manner, it was found that TBHP markedly reduced HUVEC neovascularization, while PF pretreatment prevented TBHP effects (Figures 4A,D). Next, to elucidate the role of PF on HUVEC migration, transwell examination was performed. It was proved that markedly fewer cells had underwent migration after treated with TBHP, while PF reversed these effects (Figures 4B,E). Finally, to assess the effect of PF on HUVEC adhesion, a fibronectin adhesion assay was performed (Figures 4C,F). Based on the analysis, TBHP stimulation markedly lowered the quantity of adherent cells, and this effect was reversed by PF treatment.

### Paeoniflorin Protects HUVECs From TBHP-Induced Oxidative Stress *via* HO-1 Activation

**FIGURE 5:**
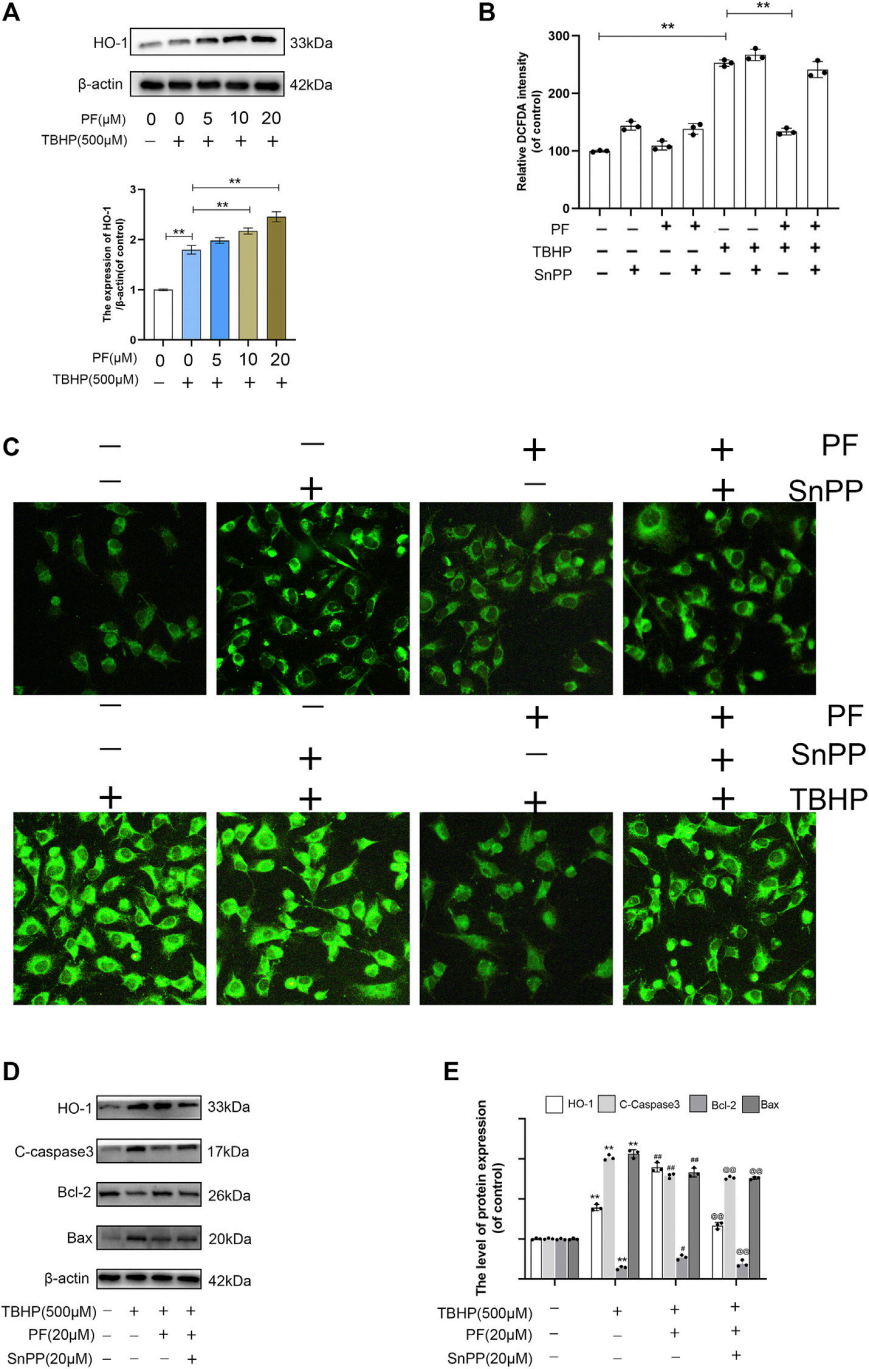
Paeoniflorin (PF) protected HUVECs from TBHP-induced oxidative stress *via* HO-1 activation. **(A)** Evaluation of total HO-1 protein by Western blotting. **(B)** Percentage of DCFDA staining intensity under different treated groups and detected by fluorescence microscope. **(C)** Representative images of DCFDA fluorescence performed to exhibit the intracellular ROS level under different conditions. **(D,E)** Evaluation of HO-1, cleaved caspase-3, Bcl-2, and Bax proteins using Western blotting for each group treated with/without TBHP, PF, and protoporphyrin IX (SnPP). Data: mean ± SD, **p < 0.01 vs controls. #*p* < 0.05, ##*p* < 0.01 vs. TBHP-stimulated HUVECs. @*p* < 0.05, @@*p* < 0.01 vs. TBHP+PF co-treated HUVECs. *n* = 3.

Nrf2 nuclear translocation directly regulates HO-1 promoter activity, which protects cells against oxidative stress and a variety of toxins (Ndisang, 2017). It was explored in the study whether PF could upregulate HO-1 under the oxidative stress condition. The results have shown that compared with the TBHP-processed group, PF had significantly increased HO-1 expression in a concentration-dependent manner (Figure 5A). The role of HO-1 in PF-mediated cytoprotection was demonstrated using SnPP, a known HO-1 inhibitor. The existence of SnPP vastly eliminated the inhibition of DCFDA intensity by PF mediation in TBHP-induced oxidative stress, which markedly promoted the intensity regardless of the presence of PF, indicating that PF inhibited ROS production by activating HO-1 expression (Figures 5B,C). The results have shown that HO-1 acted as the key detoxifying enzyme against TBHP-induced oxidative stress conditions. The Western blotting results also showed that TBHP, combined with HO-1 inhibitor SnPP, attenuated the protective effect on HUVECs induced by PF (Figures 5D,E). In conclusion, these experimental data have suggested that PF played a cytoprotective role through the activation of Nrf2/HO-1 axis.

### Paeoniflorin-Mediated Cytoprotection *via* Promoting Nucleus Translocation of Nrf2 and Activating the Nrf2/HO1 Signaling Pathway

**FIGURE 6:**
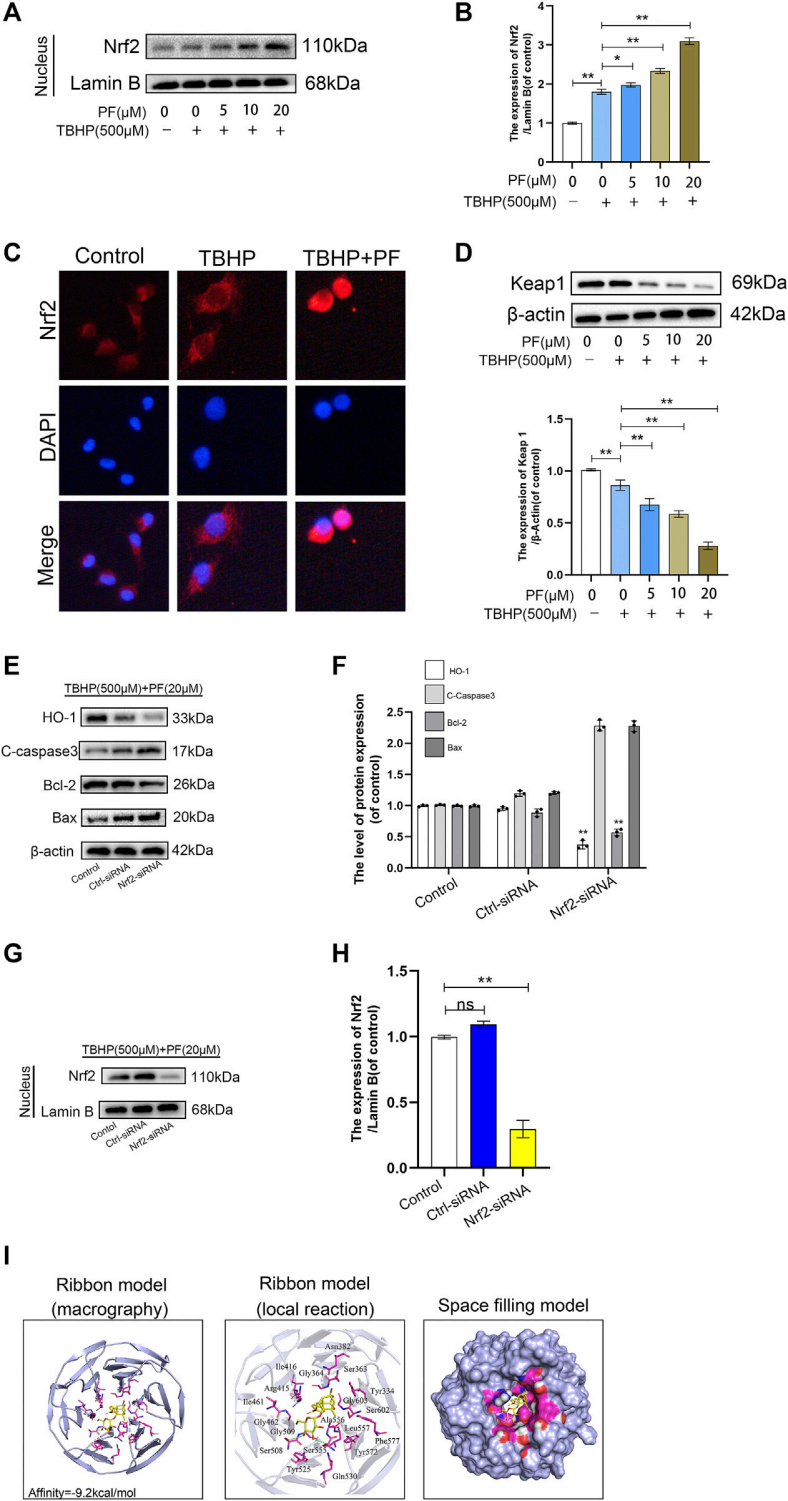
Paeoniflorin (PF)-mediated cytoprotection via promoting nucleus translocation of Nrf2 and activating the Nrf2/HO1 signaling pathway. **(A,B)** Expression of Nrf2 in the nucleus performed in the Western blotting assay and analysis Nrf2 expression in each group administrated as depicted above. **(C)** Representative immunofluorescence images of HUVECs treated with or without PF (20 μM) for 24 h and 500 μM TBHP for 2 h showing Nrf2 expression in the HUVECs. Scale bar: 20 μm. **(D)** Expression of Keap1 performed in Western blotting assay and analysis Keap1 expression in each group administrated as depicted above. **(E–H)** After transfection with Nrf2 small interfering ribonucleic acid, the expression of HO-1, cleaved caspase-3 (C-caspase 3), Bcl-2, Bax in the cytoplasm and Nrf2 in the nucleus were performed Western blotting assay and quantification corresponding proteins expression in each group were treated as depicted above. **(I)** Paeoniflorin (PF) interacts with nuclear factor erythroid-derived 2-like 2 (Nrf2) in a docking study. A ribbon model was used to represent protein residues, with a three-dimensional (3D) binding model shown. PF was able to dock strongly within the Nrf2 binding site (affinity = −9.2 kcal/mol). This proposed binding interaction involved PF interacting with Ser508 and Ser55 on Nrf2. The binding of PF in the Nrf2 pocket was shown using a space-filling model. Data: mean ± SD, *p < 0.05 and **p < 0.01. *n* = 3.

Since Nrf2 is an important transcription factor for maintaining the balance of redox responses (Khitan et al., 2014), the beneficial ways in which PF may be involved in HUVECs exposed to TBHP-mediated oxidative stress were investigated. First, nucleoprotein components were prepared to assess the expression of Nrf2 in different stimulation states through Western blotting. PF and TBHP co-treatments significantly upregulated the expression of Nrf2 and its nuclear translocation. However, Nrf2 was only slightly increased under the stimulation of TBHP. In particular, pretreatment with PF significantly increased the nuclear Nrf2 expression induced by TBHP, allowing much Nrf2 to translocate to the nucleus (Figures 6A,B). Immunostaining further confirmed the nuclear translocation of Nrf2 (Figure 6C). In addition, Western blotting showed that PF had significantly downregulated Keap1 expression with or without TBHP stimulation (Figure 6D), indicating that PF-mediated degradation of Keap1 would cause Nrf2 to migrate to the nucleus. And Nrf2 silencing abolished the PF-induced cytoprotection. After transfection with Nrf2 small interfering ribonucleic acid, Western blot analysis showed that small interfering RNA against Nrf2 had markedly diminished nuclear Nrf2 levels and reduced cytoplasmic HO-1 levels in TBHP and PF co-exposed HUVECs (Figures 6G,H). Furthermore, Nrf2 siRNA reversed PF-induced alteration of apoptotic proteins (Figures 6E,F). Furthermore, whether there was any affinity between PF and Nrf2 or upstream proteins in the Nrf2 pathway was assessed *via* computational molecular docking analysis (Luo et al., 2020). For this analysis, the PF chemical structure was used, as shown in Figure 1A. After the examination of all generated models, PF was found to have clearly interacted with and docked in the Nrf2 binding site (Figure 6I), with macro- and local-level views of these interactions shown using a ribbon model. A space-filling model was additionally used to illustrate this interaction. High-affinity (−9.2 kcal/mol) hydrogen binding events between PF and the Ser508 and Ser555 residues of Nrf2 were observed, indicating that PF might have functioned to partially inhibit the development of the flap survival through its ability to interact with Nrf2 in a manner promoting its nuclear translocation. These results indicated that PF treatment had significantly stimulated the nuclear translocation of Nrf2, leading to the activation of antioxidant defense mechanisms.

### Paeoniflorin Promotes the Skin Flaps Survival in Rats

**FIGURE 7:**
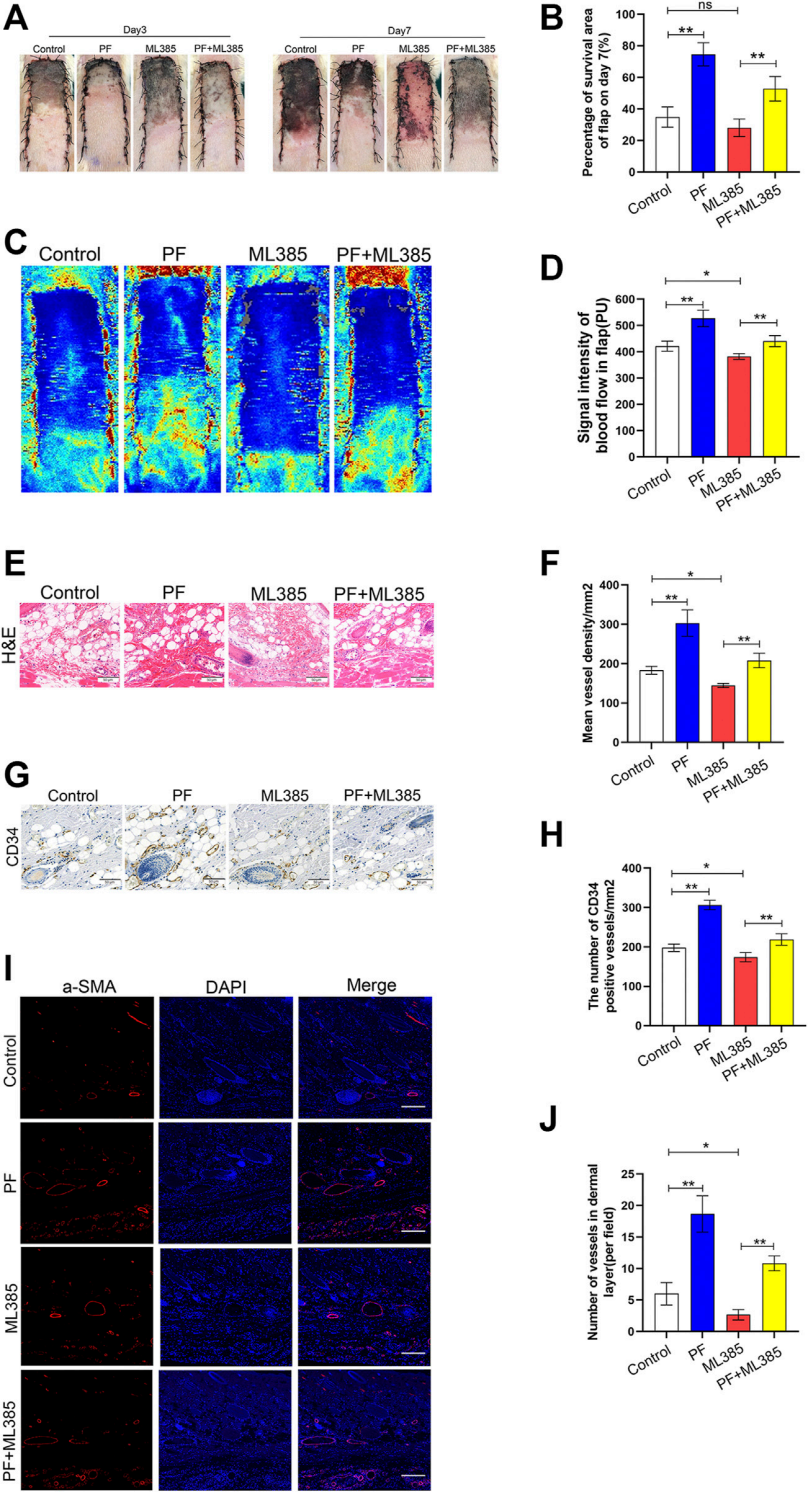
Paeoniflorin (PF) enhances random-pattern skin flaps survival in rats. **(A)** Digital images of the survival/necrotic area from three groups on POD 3 and 7. **(B)** Evaluation of survival area on POD 7. **(C)** Evaluation of Blood flow and vascular distribution by Laser Doppler Blood Flow in each group. **(D)** Histogram showed the intensity of the blood flow signal. **(E)** Evaluation of CD34 marking vessels in vascular endothelial cells using IHC (200×; scan bar, 50 μm). **(F)** Evaluation of CD34^+^ vessel density (%) using histogram. **(G)** Evaluation of subcutaneous blood vessels and inflammation via H&E staining in different groups (200×; scan bar, 50 μm). **(H)** Flap mean vessel density (MVD) assessment (/mm^2^) using histogram. **(I)** Evaluation of a-SMA expressing vessels in vascular endothelial cells, using immunofluorescence (200×; scan bar, 50 μm). **(J)** Quantification of a-SMA positive vessel density in dermal layer using histogram. Data: mean ± SD, *p < 0.05 and **p < 0.01. *n* = 6.

After operation, the distal skin flap appeared pale and swollen (Figure 7A). Tissue necrosis was first evident in zone III, where it became dry, crumpled, and dark. Necrosis gradually spread from distal areas to the proximal segment on POD 7. Based on our analysis and compared to PF+ML385-treated animals, a larger percentage of the random-pattern skin flaps survived in the PF-treated animals (34.90 ± 5.85%, 74.60 ± 6.69%, 28.06 ± 5.06% and 52.84 ± 7.13%, respectively, corresponding to the control group, PF group, ML385 group, and PF+ML385 group; Figure 7B). To assess whether PF affected microvascular network on the rat’s back, laser doppler was used to visualize the blood flow. It was revealed that compared to the control group, the PF-treated animals had evident blood flow signal in the flaps. Moreover, the enhanced blood flow was impaired in animals treated with PF+ML385 (421.36 ± 17.48PU, 527.04 ± 28.52PU, 383.24 ± 9.89PU, and 417.43 ± 10.42PU, respectively, corresponding to the control group, PF group, ML385 group, and PF+ML385 group; Figures 7C,D). Next, multiple angiogenesis markers have been used to evaluate the level of neovascularization in the PF-treated flaps. Hematoxylin–eosin staining results have demonstrated that PF-treated animals had remarkably more vessel density than those in the control group. Conversely, a combined treatment of PF+ML385 had dramatically reduced vessel density comparing to PF treatment alone (174.17 ± 13.64/mm^2^, 303.17 ± 30.62/mm^2^, 154.17 ± 13.26/mm^2^, and 190.00 ± 14.21/mm^2^, respectively, corresponding to the control group, PF group, ML385 group, and PF+ML385 group; Figures 7E,F). Similarly, the quantity of CD34-positive vessels in PF-treated animals was significantly higher than that of the controls, and the opposite was observed in animals co-treated with PF+ML385 (197.83 ± 8.57/mm^2^, 306.50 ± 11.06/mm^2^, 185.83 ± 7.00/mm^2^, and 218.83 ± 13.52/mm^2^, respectively, corresponding to the control group, PF group, ML385 group, and PF+ML385 group; Figures 7G,H). It was also explored whether the positive effect of PF on flap survival might be obtained *via* enhancing angiogenesis. IHC was employed for the detection of VEGF expression. Compared to the control group and the PF+ML385 group, VEGF expression was considerably enhanced in the flap II area of the PF group, while VEGF expression in the ML385 group was significantly lowered, as depicted in Supplementary Figures S1A,B. The results were consistent with those obtained from immunoblotting (Supplementary Figures S1C,D). Consistent with these results, immunofluorescence was employed for the detection of a-SMA–positive microvessels, which were more significant in the PF-treated animals than in the control animals, while the PF+ML385 co-treatment vastly reduced the a-SMA positive microvessel quantity comparing to PF treatment alone (Figures 7I,J). In all, these data have suggested that PF promoted angiogenesis and played a crucial role in improving the skin flap viability.

### Paeoniflorin Mediates Protection via the Nrf2 Pathway *In Vivo*

**FIGURE 8:**
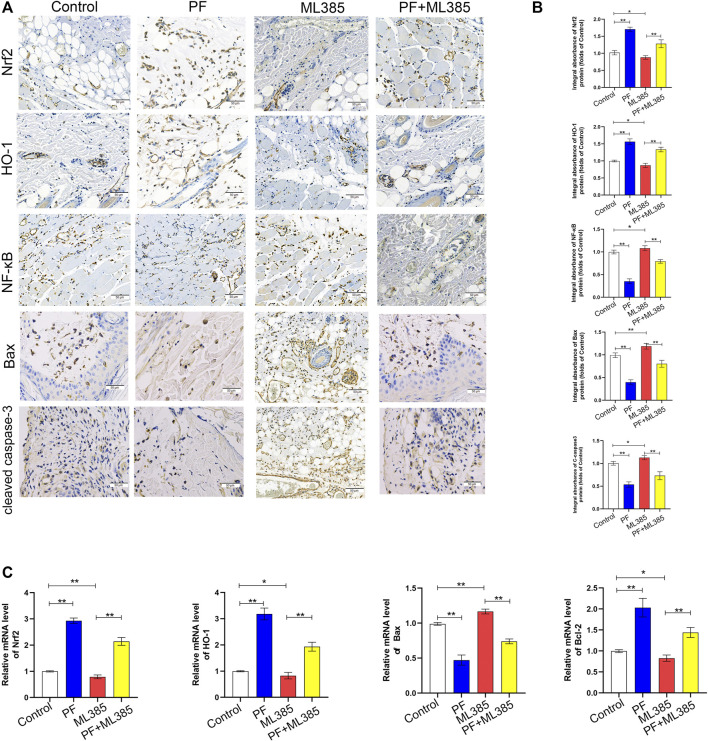
Paeoniflorin (PF)-mediated cytoprotection involved the Nrf2 pathway *in vivo*. **(A)** IHC of proteins Nrf2, HO-1, NF-κB, Bax and cleaved caspase-3 in ischemic tissues (original magnification, 200×; scan bar, 50 μm). **(B)** Quantification of integral absorbance of Nrf2, HO-1, NF-κB, Bax and cleaved caspase-3 in IHC. **(C)** The contents of Nrf2, HO-1, Bax, and Bcl-2 genes in the skin tissue were determined by real-time quantitative PCR and the data were normalized to GAPDH. (*n* = 6 mice per group). Data: mean ± SD, *p < 0.05 and **p < 0.01. *n* = 6.

To explore whether PF modulates the Nrf2 pathway *in vivo*, the levels of Nrf2 pathway-related markers with IHC were evaluated. IHC and integrated absorbance analysis indicated that the Nrf2 and HO-1 expression in the PF group were considerably increased compared to the control group and the PF+ML385 group. The Nrf2 and HO-1 expression were decreased in the ML385 group; however, no considerable variations were observed between the control group and the PF+ML385 group, as depicted in Figures 8A,B. Additionally, the level of NF-κB, C-caspase3, and Bax in the PF group analyzed by IHC showed significantly lower expression than that of those in the control group. And these protective effects of PF-induced were decreased by PF co-administration with ML385. The data of real-time quantitative PCR assays have demonstrated that transcription of Nrf2, HO-1, and Bcl-2 was reduced after being treated with ML385 (Figure 7C). The results also showed that the level of Bax expression was increased in the ML385 group compared to the control group and the PF+ML385 group, while no considerable variations were observed between the control group and the PF+ML385 group, as depicted in Figure 8C. To sum up, the IHC and real-time quantitative PCR assay results showed that PF promoted the skin flap viability *via* Nrf2/HO-1 signaling pathway *in vivo*.

## Discussion

**FIGURE 9:**
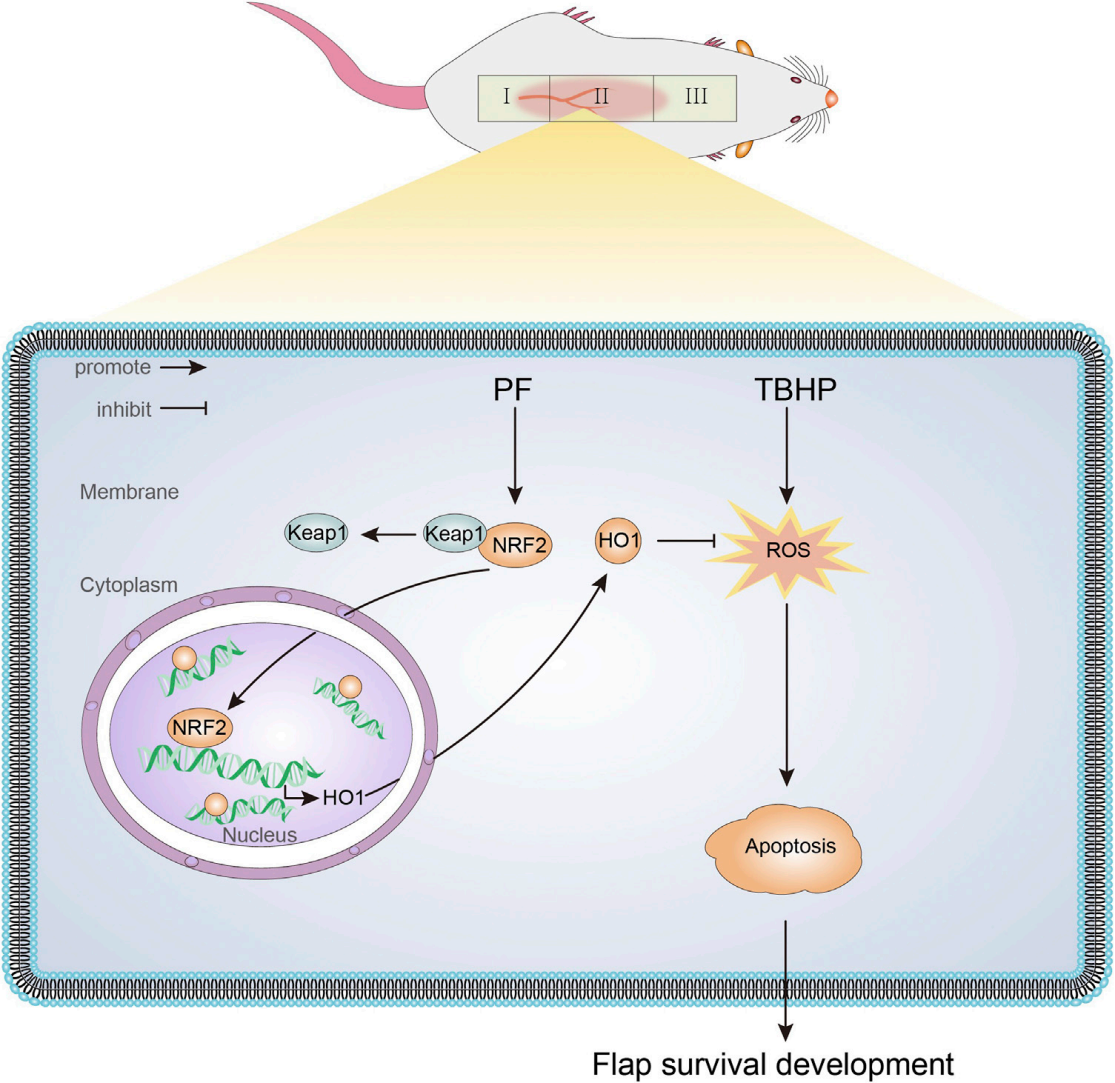
A schematic diagram illustrating the role of PF in protecting against oxidative stress *via* the Nrf2/HO-1 signaling pathway.

In this study, we first demonstrated that PF had a protective effect on TBHP-induced oxidative stress, mitochondrial dysfunction, and cell injury in HUVECs. As shown in Figure 9, PF mechanically activated the Nrf2/HO-1 antioxidant stress pathway and subsequently reduced intracellular ROS accumulation and mitochondrial superoxide levels in HUVECs. Additionally, it was also found that PF promoted the random-pattern skin flaps survival by activating the Nrf2/HO-1 axis. The obtained data revealed that ML385, the Nrf2 inhibitor, abolished PF activation on tissues.

The random-pattern skin flap survival requires a series of intricate events including fighting against ischemic necrosis. However, ischemic necrosis is a common complication, especially in the distal parts of the skin flaps (Zhou et al., 2021). Ischemia-reperfusion injury is mostly caused by oxidative stress and apoptosis, leading to further aggravation of tissue damage (Gottlieb and Engler, 1999; Kannan and Jain, 2000). Being a double-edged sword additionally, ROS and ROS signaling, is an important mechanism of inflammation, including migration, proliferation, and angiogenesis (Monkkonen and Debnath, 2018). Several studies have shown that a low continuous stimulation of ROS could vastly improve the skin flap survival. However, excessive activation can lead to cellular apoptosis (Rezaeian et al., 2010). Given the antioxidant properties of PF, its effect in attenuating TBHP-stimulated cell damage was examined.

Exogenous TBHP may stimulate intracellular ROS production by simulating endogenous ROS signaling pathways. Acting as a second messenger like calcium ions, TBHP can initiate signal cascades through high diffusion of the plasma membrane (Li et al., 2019), eventually resulting in the formation and accumulation of ROS, thus inhibiting cell proliferation and promoting apoptosis mediated by mitochondrial dysfunction (Liu et al., 2016). A previous study has reported that Nrf2 upregulates Bcl2 (Niture and Jaiswal, 2012). In normal physiological environment, mitochondrial membrane potential balance is strictly controlled. However, TBHP induces apoptosis through the increasing generation of mtROS associated with the interruption of mitochondrial membrane potential, promoting the release of cytochrome C from mitochondria to cytoplasm by activating apoptotic protein Bax and inactivating antiapoptotic protein Bcl-2 (Ott et al., 2002). The released cytochrome C activates caspase-9 and in turn stimulates the caspase effect, which hydrolyzes the intracellular DNA repair enzyme RARP and eventually leads to irreversible cell death (Liu et al., 2016). The results in this study have shown that PF protected HUVECs from TBHP-induced apoptosis by inhibiting the production of mtROS and inhibiting caspase, suggesting that PF is an effective antioxidant that can protect HUVECs from oxidative stress. PF pretreatment could significantly improve cellular dysfunction caused by TBHP stimulation on HUVECs. In addition, PF could also restore TBHP-induced mitochondrial membrane potential depolarization and downregulate the Bax/Bcl-2 ratio.

In a previous study, small interfering RNA (siRNA)-mediated Nrf2 gene silencing increased sensitivity to TBHP-induced cytotoxicity by downregulating HO-1 expression (Jian et al., 2011), indicating that Nrf2-mediated HO-1 could reduce ROS production and thus ameliorate oxidative stress–mediated apoptosis. In particular, mtROS could promote cytochrome C release from mitochondria through activating the internal pathway under the condition of oxidative stress, which was mediated by caspase-9 (Cheng et al., 2019), leading to mitochondrial membrane potential disturbance and therefore increasing cell apoptosis. However, Nrf2 activation, to some extent, can be a double-edged sword, especially for certain types and stages of cancer because activation of Nrf2 can boost cancer cell survival (Ikeda et al., 2004). However, it was found in our study that PF enhanced the expression of HO-1 and Nrf2 nuclear translocation, thus protecting HUVECs from TBHP-induced apoptosis by preserving mitochondrial membrane potential and inhibiting the generation of mtROS. In addition, the effect of SnPP, a specific HO-1 inhibitor, reversed the cytoprotective effect of PF in TBHP-induced oxidative stress, suggesting that Nrf2-mediated HO-1 activation had played a critical role in protecting TBHP-induced HUVECs from oxidative stress injury. In addition, Nrf2−/− deficient mice (Nrf2−/−) had higher susceptibility to carcinogenesis (Yokoo et al., 2016) and increased metastatic ability of cancer cells (Zhu et al., 2016). Therefore, increasing Nrf2/HO-1 activity is a promising field for the treatment of traumatic stress and cancer.

To elucidate PF activity *in vivo*, the random-pattern skin flap was performed to evaluate the flap survival on the seventh day. Based on our analysis and compared to animals in the control group, PF-treated animals exhibited enhanced skin flap viability. To evaluate PF’s activity on angiogenesis, H&E and IHC staining of CD34-positive vascular cells were carried out, and it was found that PF had increased the density of blood vessels in the flap tissue. Then whether FGF21 modulated VEGF was investigated, which were found to have enhanced the angiogenesis. VEGF contributed to multiple processes of angiogenesis (particularly mitosis of vascular cells), promoting the formation and maturation of neovascularization. The results of the study have shown that with PF treatment, VEGF was increased in the ischemic flap tissue, indicating that PF was a promoter of angiogenesis in the rats’ random-pattern flap model. However, once administered the Nrf2 inhibitor ML385, the potent PF oxidative response disappeared. Using IHC, it was revealed that the Nrf2 and HO-1 signals were augmented in the PF-treated animals compared with the control group, and these signals were reduced in animals co-treated with PF and ML385. Similarly, it was proved that the NF-κB, Bax and C-caspase three levels were low in the PF-treated animals compared with the control group. However, the proteins were highly expressed in animals receiving combined PF+ML385 treatment compared with the ML385 group. Collectively, these findings have clearly shown the efficacy of PF in stimulating the Nrf2 signaling network in order to alleviate oxidative damage. Hence, PF treatment could vastly improve the random-pattern skin flap survival *via* stimulation of the Nrf2/HO-1 pathway. In conclusion, these findings indicated the potential of PF in enhancing the skin flap survival in transplant and plastic surgery patients.

## Conclusion

In summary, based on our *in vivo* and *in vitro* data, PF can serve as a potential therapy for enhancing the random-pattern skin flap survival. Its protective function on endothelial fibrosis has been demonstrated, followed by its attenuation of oxidative stress–induced apoptosis and amelioration of endothelial cell function. The significance of the Nrf2/HO-1 pathway in mediating the cellular actions of PF has also been revealed.

## Data Availability

The datasets presented in this study can be found in online repositories. The names of the repository/repositories and accession number(s) can be found in the article/Supplementary Material.

## Supplementary Material

Supplementary Figure S1 Paeoniflorin (PF) upregulated angiogenesis in flaps. On the 7th day after operation, the samples were harvested for the evaluation of angiogenesis in ischemic skin flaps. **(A)** IHC of proteins VEGF in ischemic skin flaps (original magnification, 200×; scan bar, 50 μm). **(B)** Quantification of integral absorbance of VEGF in IHC. **(C)** Western blotting performed for expression of VEGF, which was corrected by β-actin as an internal control. **(D)** Quantization of optical density values of VEGF expressions in the flaps. Significance: **p* < 0.05 and ***p* < 0.01 vs. control group. Data were expressed as means ± SD (*n* = 6 per group).
