# Supplementary figures and images for: Paeoniflorin Suppresses TBHP-Induced Oxidative Stress and Apoptosis in Human Umbilical Vein Endothelial Cells via the Nrf2/HO-1 Signaling Pathway and Improves Skin Flap Survival (part 2 of 2)
Source: Front Pharmacol. 2021 Nov 4;12:735530. doi: 10.3389/fphar.2021.735530 (PMC8600365; doi:10.3389/fphar.2021.735530)

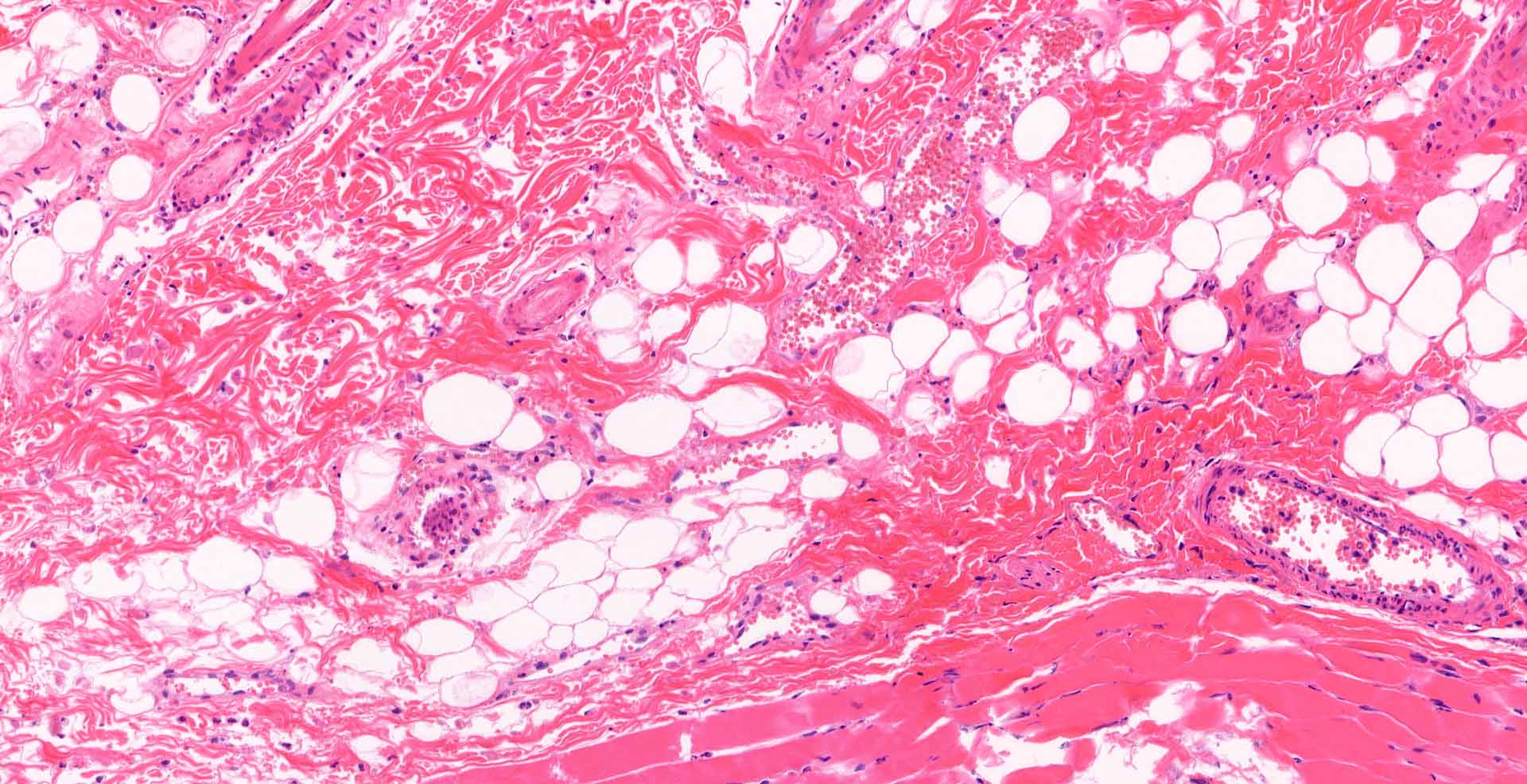

Supplement: Supplementary file 1 [file DataSheet1.ZIP › Raw Data/figure7/fig7. E/PF.jpg]

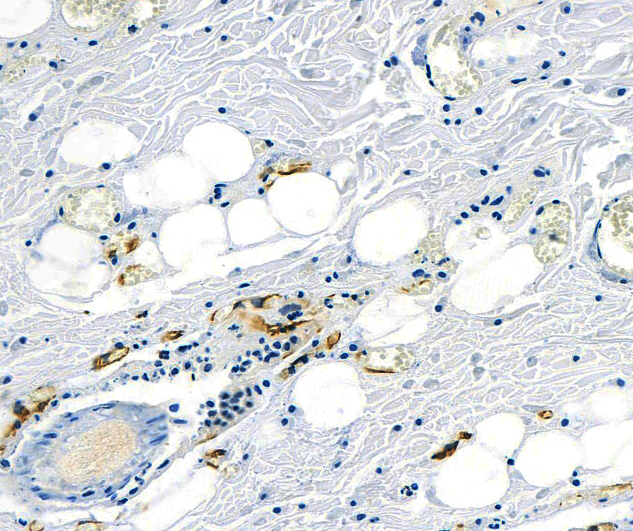

Supplement: Supplementary file 1 [file DataSheet1.ZIP › Raw Data/figure7/fig7. G/control.jpg]

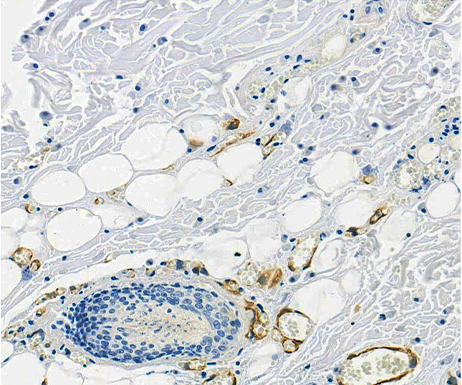

Supplement: Supplementary file 1 [file DataSheet1.ZIP › Raw Data/figure7/fig7. G/ML385.jpg]

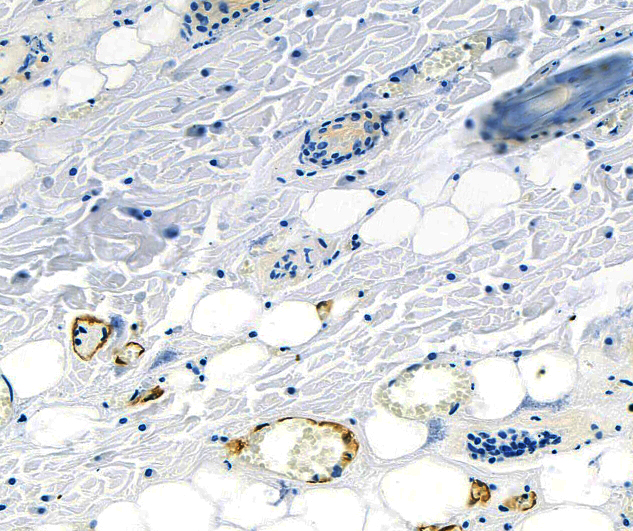

Supplement: Supplementary file 1 [file DataSheet1.ZIP › Raw Data/figure7/fig7. G/PF+ML385.jpg]

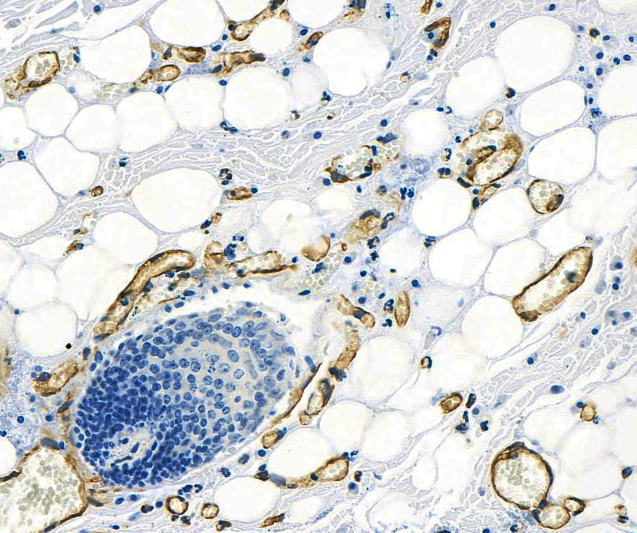

Supplement: Supplementary file 1 [file DataSheet1.ZIP › Raw Data/figure7/fig7. G/PF.jpg]

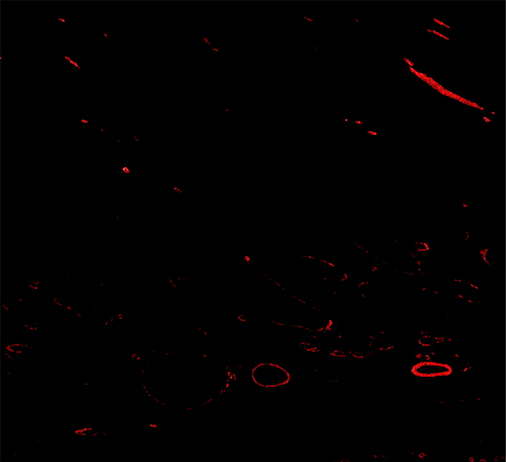

Supplement: Supplementary file 1 [file DataSheet1.ZIP › Raw Data/figure7/fig7. I/Control a-SMA.jpg]

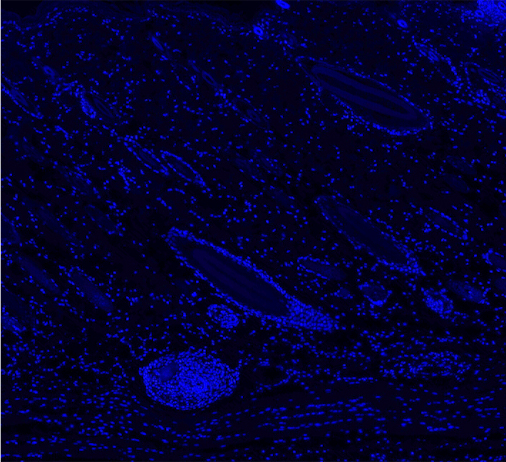

Supplement: Supplementary file 1 [file DataSheet1.ZIP › Raw Data/figure7/fig7. I/control DAPI.jpg]

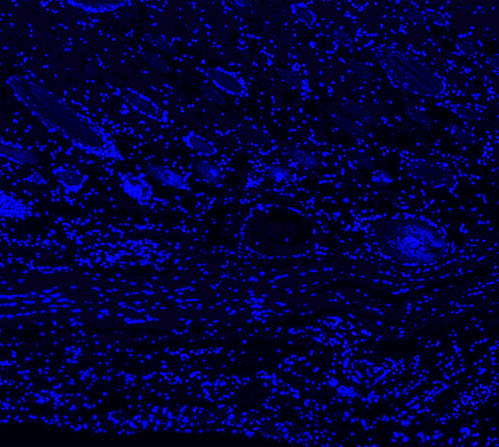

Supplement: Supplementary file 1 [file DataSheet1.ZIP › Raw Data/figure7/fig7. I/ML385 DAPI.jpg]

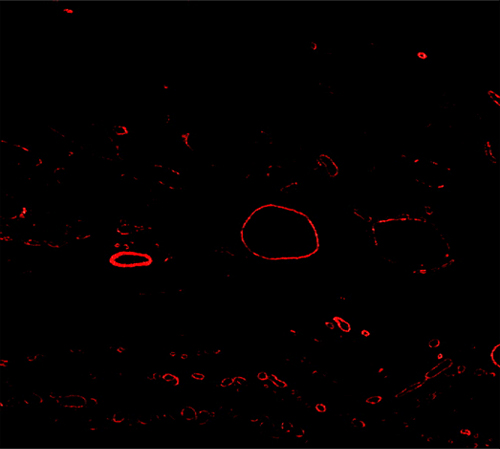

Supplement: Supplementary file 1 [file DataSheet1.ZIP › Raw Data/figure7/fig7. I/ML385.jpg]

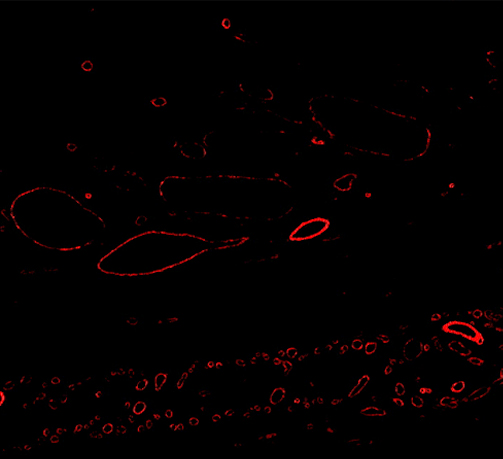

Supplement: Supplementary file 1 [file DataSheet1.ZIP › Raw Data/figure7/fig7. I/PF a-SMA.jpg]

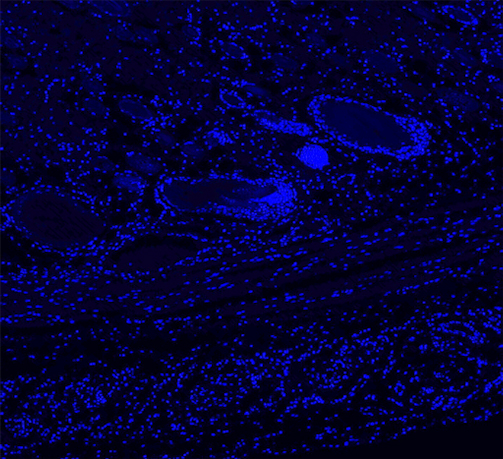

Supplement: Supplementary file 1 [file DataSheet1.ZIP › Raw Data/figure7/fig7. I/PF DAPI.jpg]

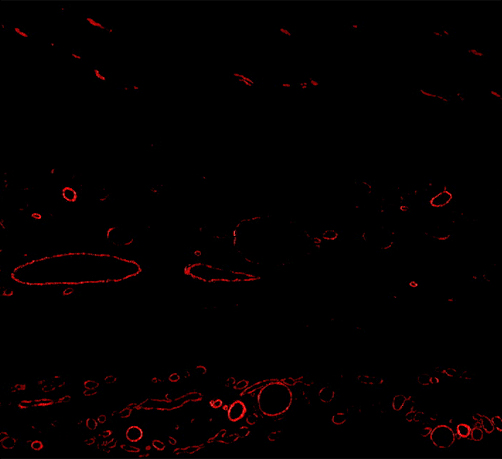

Supplement: Supplementary file 1 [file DataSheet1.ZIP › Raw Data/figure7/fig7. I/PF+ML385 a-SMA.jpg]

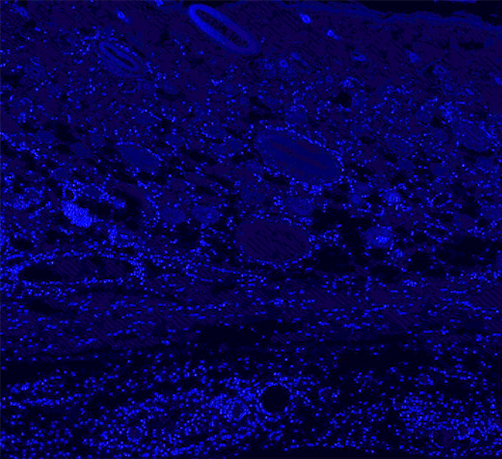

Supplement: Supplementary file 1 [file DataSheet1.ZIP › Raw Data/figure7/fig7. I/PF+ML385 DAPI.jpg]

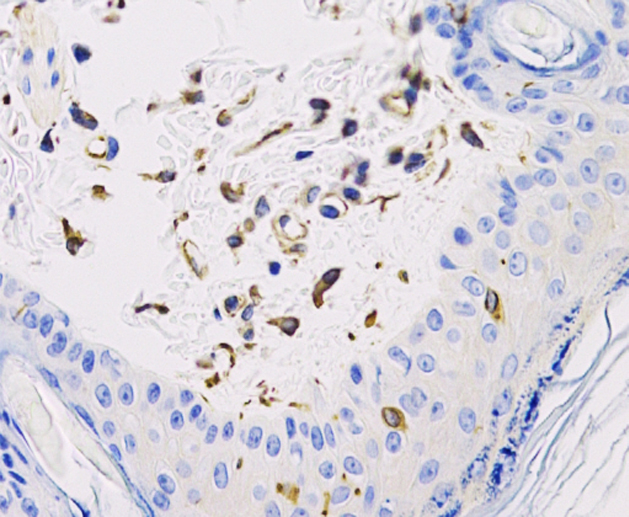

Supplement: Supplementary file 1 [file DataSheet1.ZIP › Raw Data/figure8/IHC/Bax control.jpg]

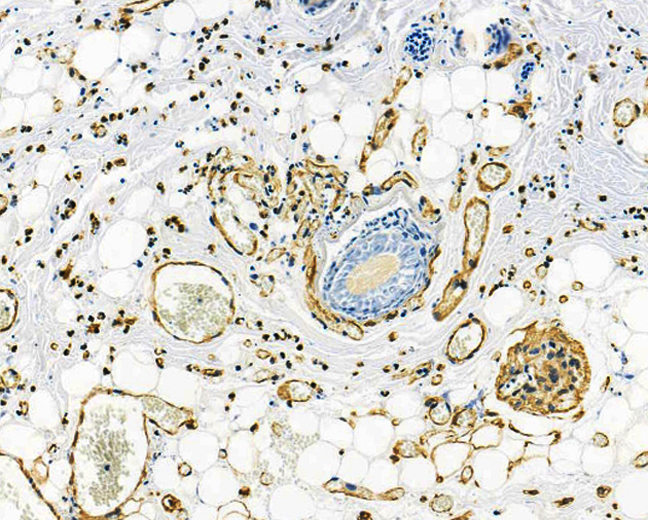

Supplement: Supplementary file 1 [file DataSheet1.ZIP › Raw Data/figure8/IHC/Bax ML385.jpg]

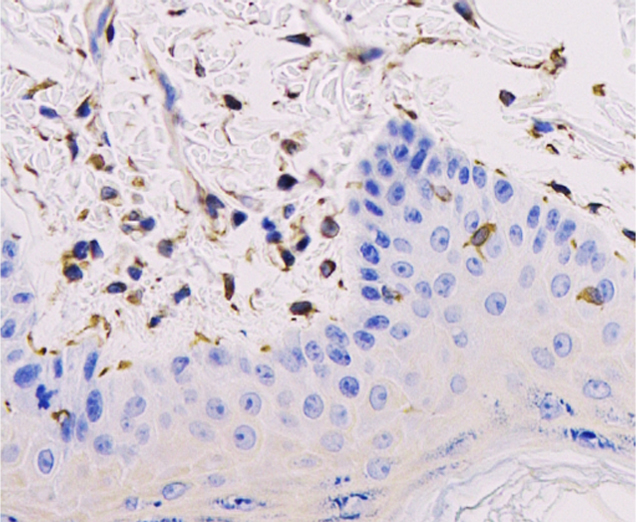

Supplement: Supplementary file 1 [file DataSheet1.ZIP › Raw Data/figure8/IHC/Bax PF+ML385.jpg]

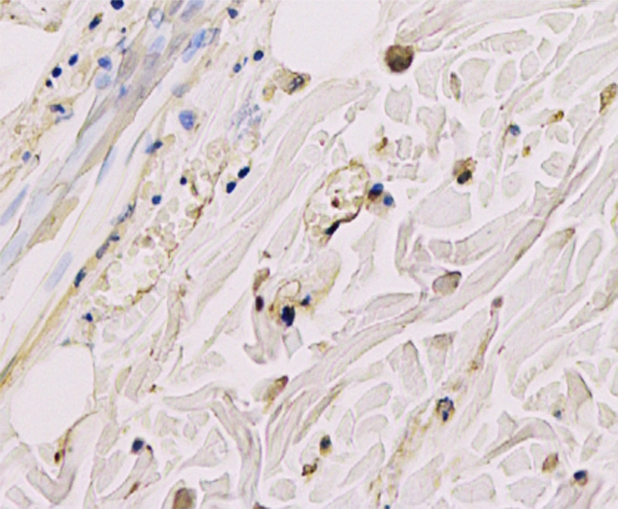

Supplement: Supplementary file 1 [file DataSheet1.ZIP › Raw Data/figure8/IHC/Bax PF.jpg]

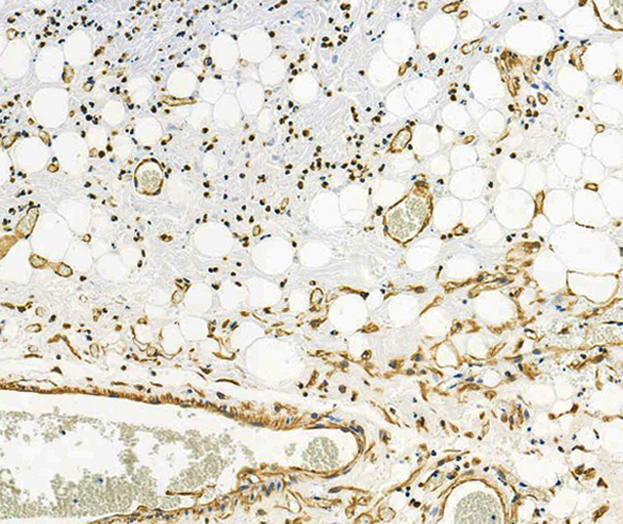

Supplement: Supplementary file 1 [file DataSheet1.ZIP › Raw Data/figure8/IHC/c-cas ML385.jpg]

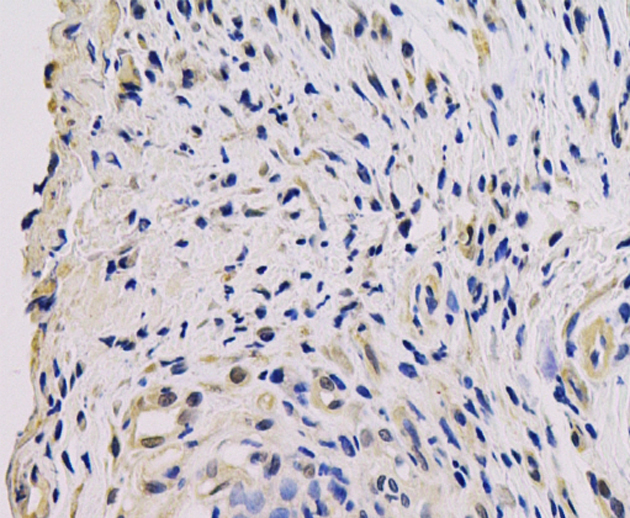

Supplement: Supplementary file 1 [file DataSheet1.ZIP › Raw Data/figure8/IHC/c-cas3 control.jpg]

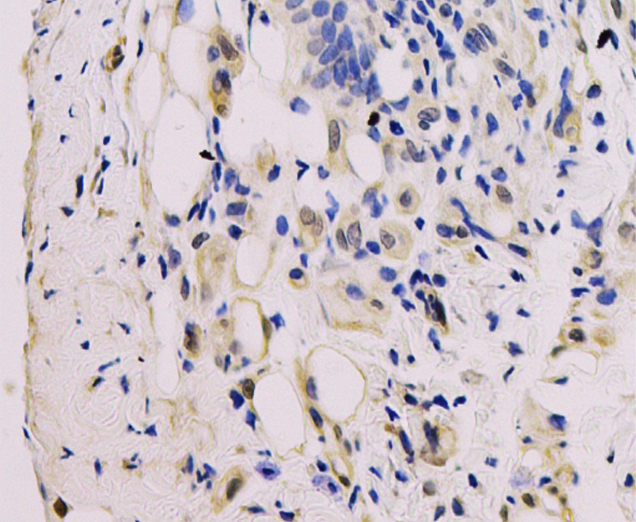

Supplement: Supplementary file 1 [file DataSheet1.ZIP › Raw Data/figure8/IHC/C-CAS3 PF+ML385.jpg]

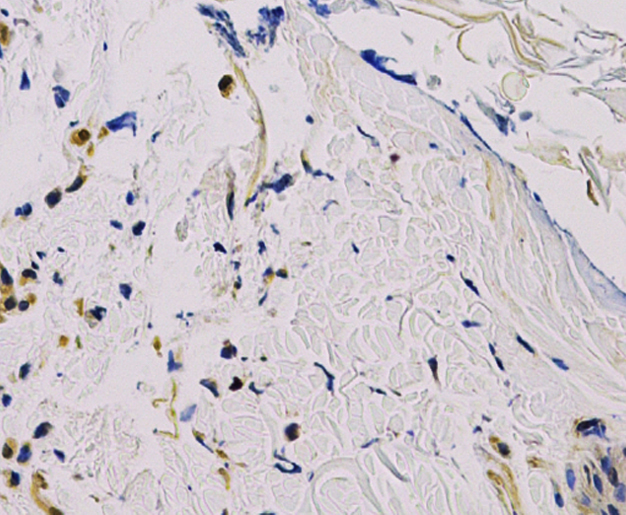

Supplement: Supplementary file 1 [file DataSheet1.ZIP › Raw Data/figure8/IHC/c-cas3 PF.jpg]

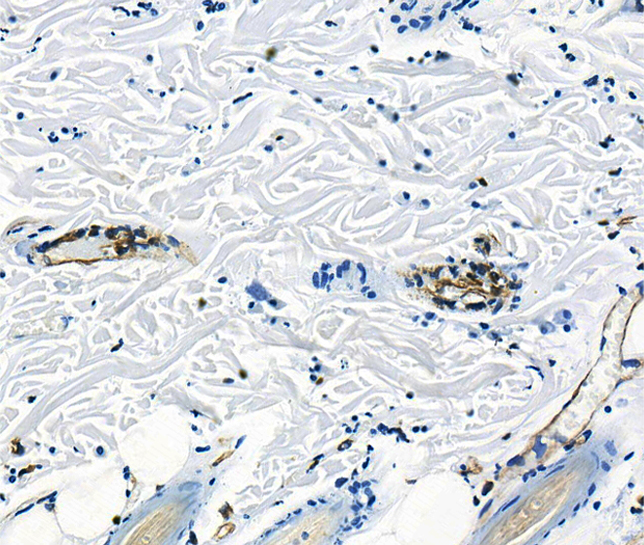

Supplement: Supplementary file 1 [file DataSheet1.ZIP › Raw Data/figure8/IHC/HO-1 control.jpg]

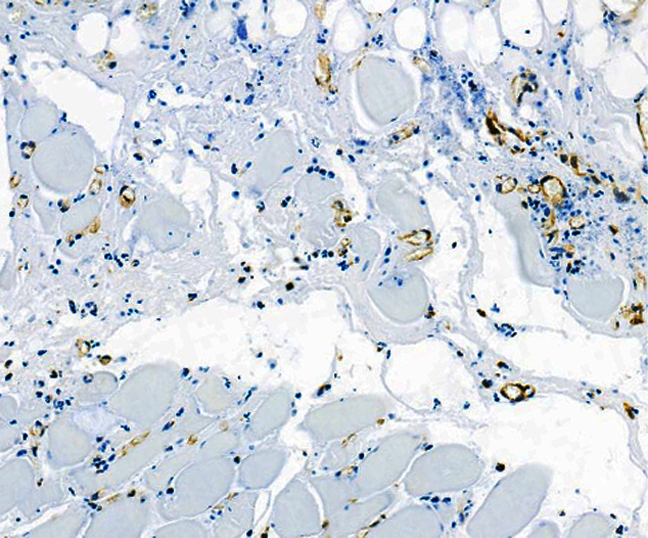

Supplement: Supplementary file 1 [file DataSheet1.ZIP › Raw Data/figure8/IHC/HO-1 ML385.jpg]

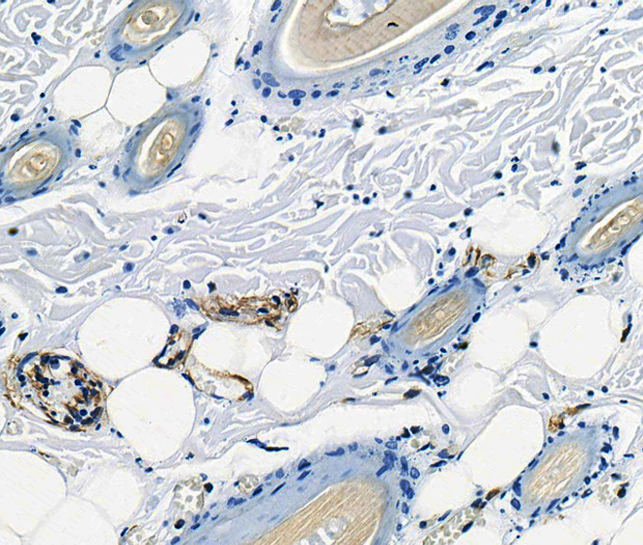

Supplement: Supplementary file 1 [file DataSheet1.ZIP › Raw Data/figure8/IHC/HO-1 PF+ML385.jpg]

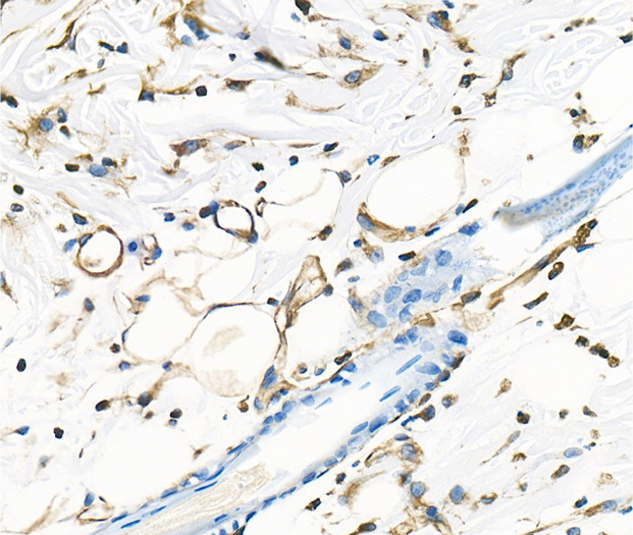

Supplement: Supplementary file 1 [file DataSheet1.ZIP › Raw Data/figure8/IHC/HO-1 PF.jpg]

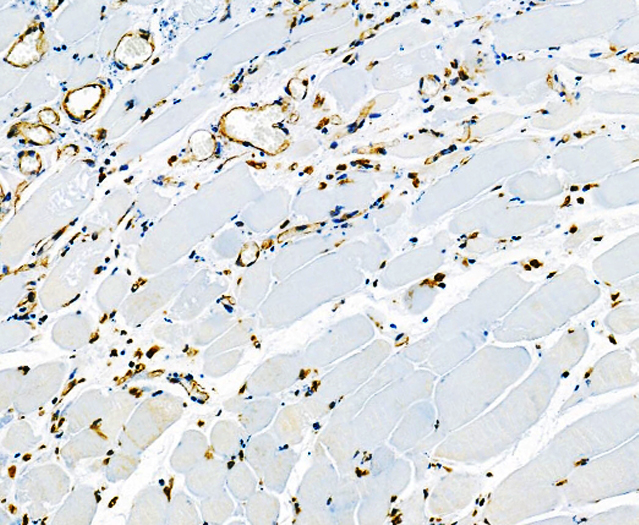

Supplement: Supplementary file 1 [file DataSheet1.ZIP › Raw Data/figure8/IHC/NF-KB control.jpg]

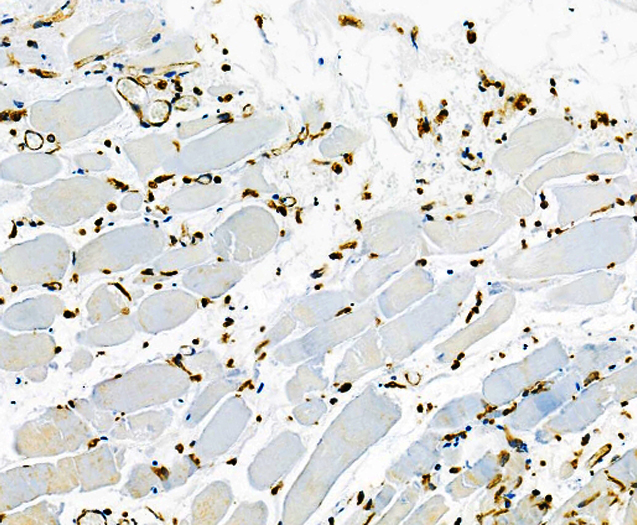

Supplement: Supplementary file 1 [file DataSheet1.ZIP › Raw Data/figure8/IHC/NF-KB ML385.jpg]

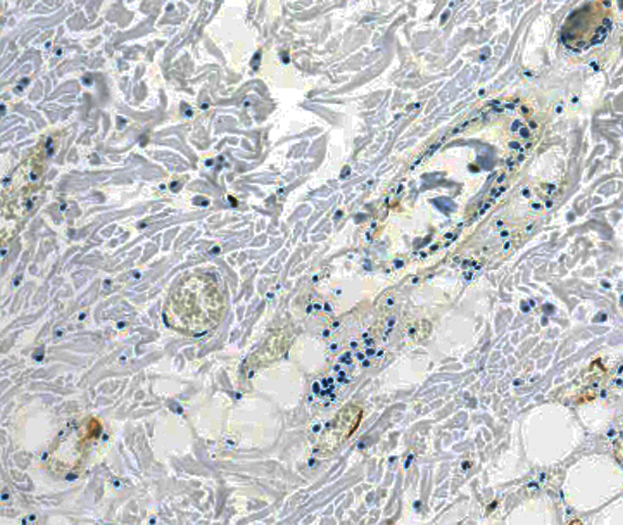

Supplement: Supplementary file 1 [file DataSheet1.ZIP › Raw Data/figure8/IHC/NF-KB PF+ML385.jpg]

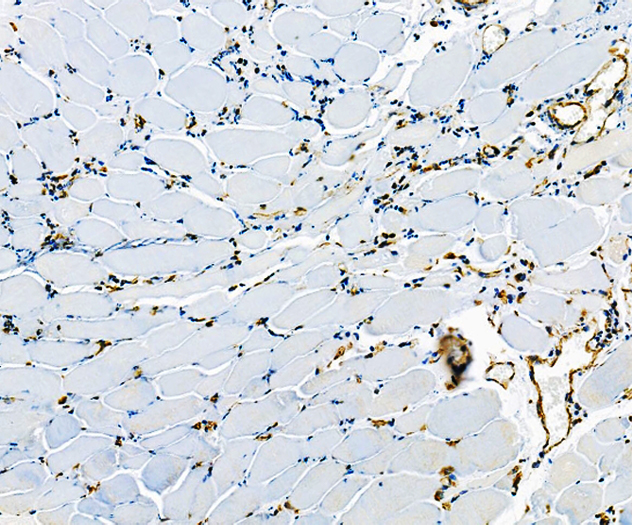

Supplement: Supplementary file 1 [file DataSheet1.ZIP › Raw Data/figure8/IHC/NF-KB PF.jpg]

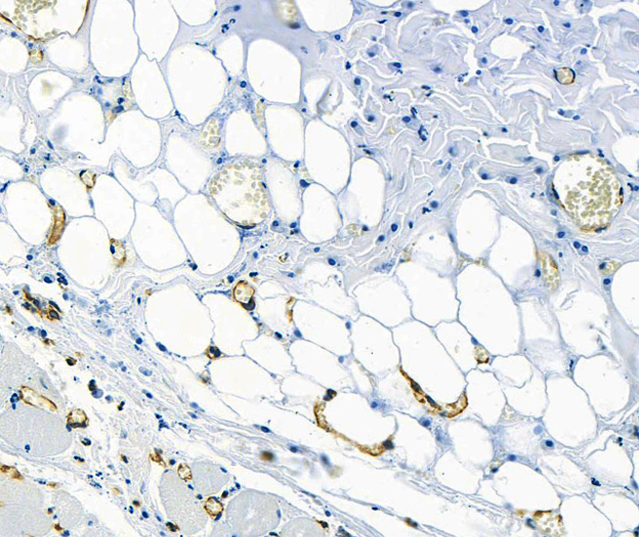

Supplement: Supplementary file 1 [file DataSheet1.ZIP › Raw Data/figure8/IHC/Nrf2 control.jpg]

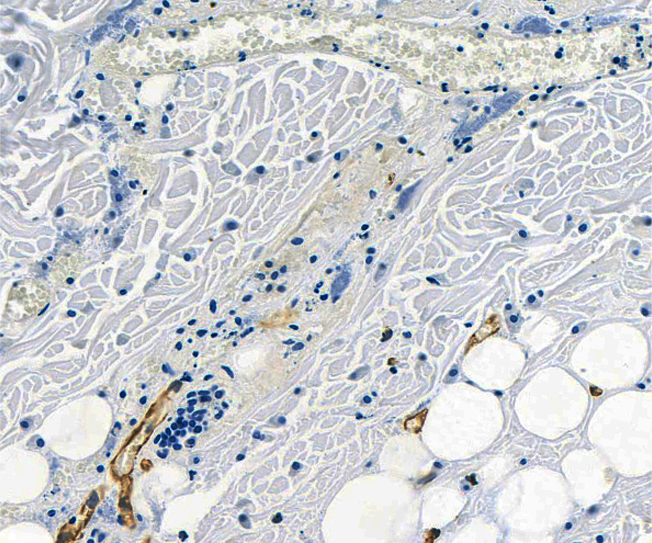

Supplement: Supplementary file 1 [file DataSheet1.ZIP › Raw Data/figure8/IHC/Nrf2 ML385.jpg]

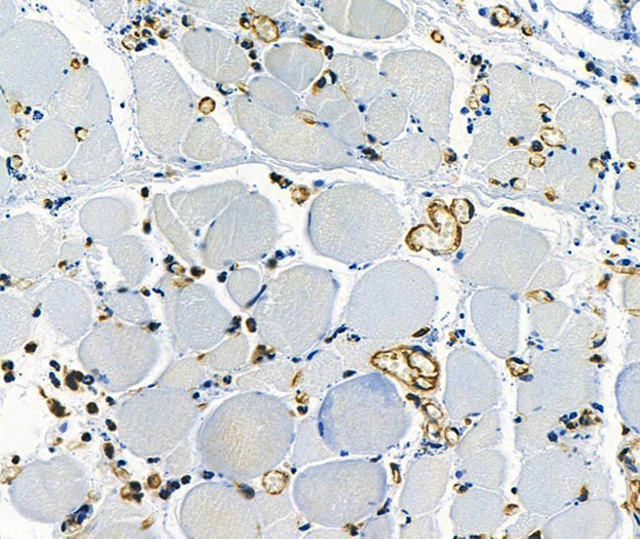

Supplement: Supplementary file 1 [file DataSheet1.ZIP › Raw Data/figure8/IHC/Nrf2 PF+ML385.jpg]

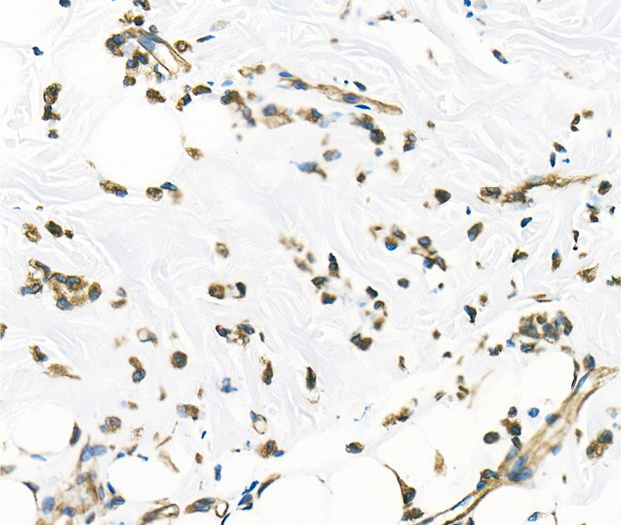

Supplement: Supplementary file 1 [file DataSheet1.ZIP › Raw Data/figure8/IHC/Nrf2 PF.jpg]

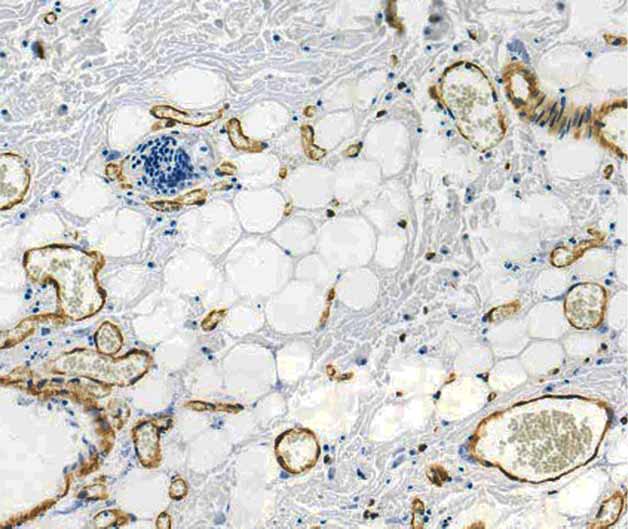

Supplement: Supplementary file 1 [file DataSheet1.ZIP › Raw Data/figureS1/control.jpg]

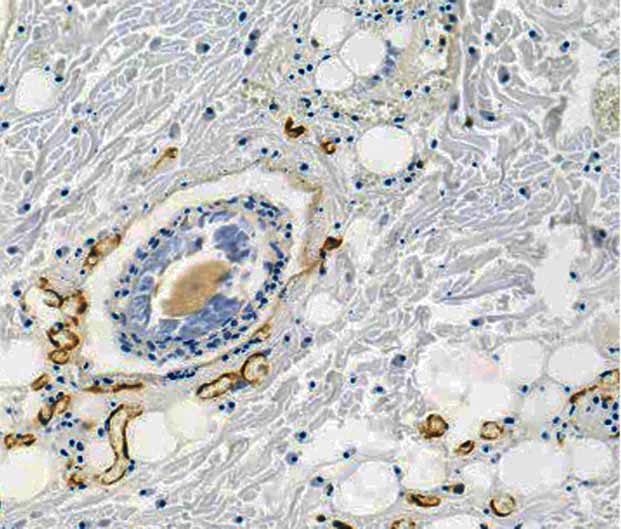

Supplement: Supplementary file 1 [file DataSheet1.ZIP › Raw Data/figureS1/ML385.jpg]

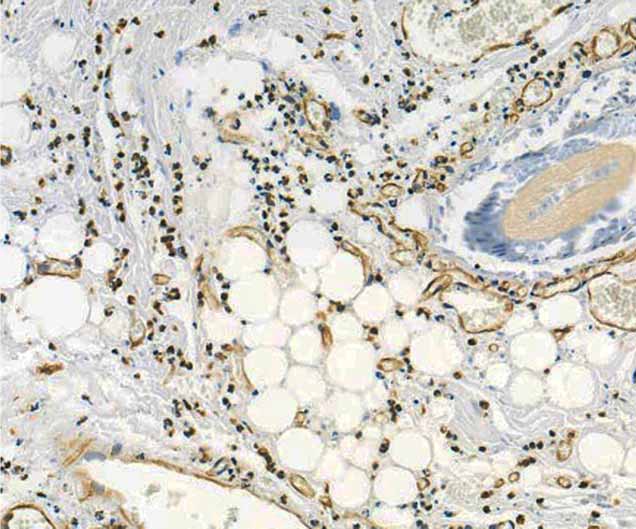

Supplement: Supplementary file 1 [file DataSheet1.ZIP › Raw Data/figureS1/PF.jpg]

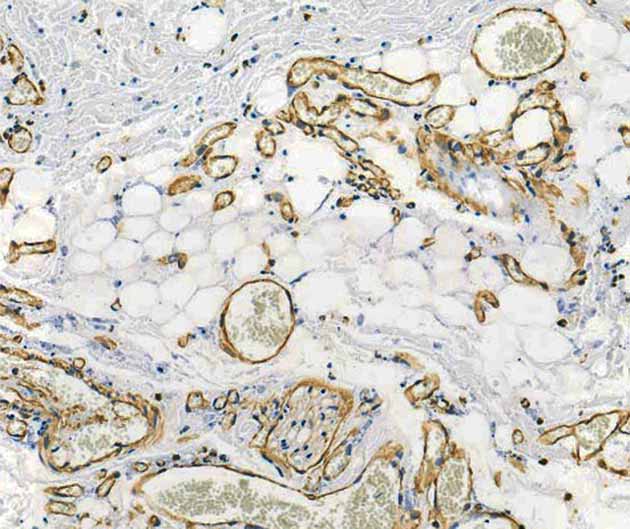

Supplement: Supplementary file 1 [file DataSheet1.ZIP › Raw Data/figureS1/PL+ML385.jpg]

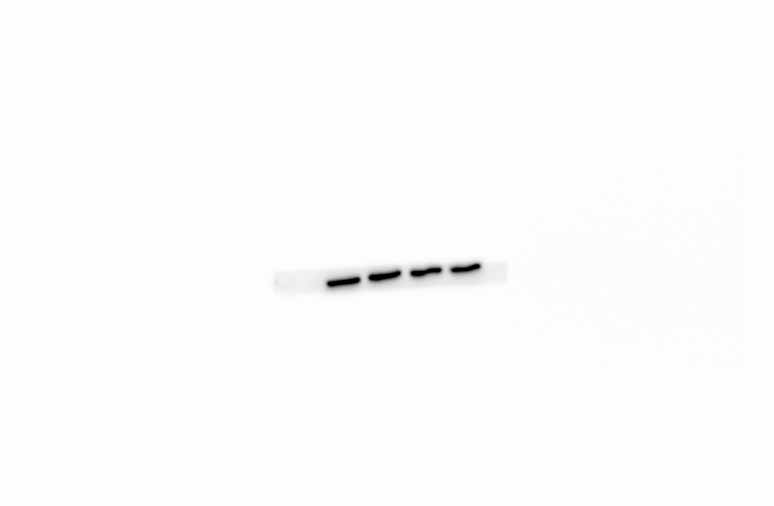

Supplement: Supplementary file 1 [file DataSheet1.ZIP › Raw Data/figureS1/WB/actin-1111.jpg]

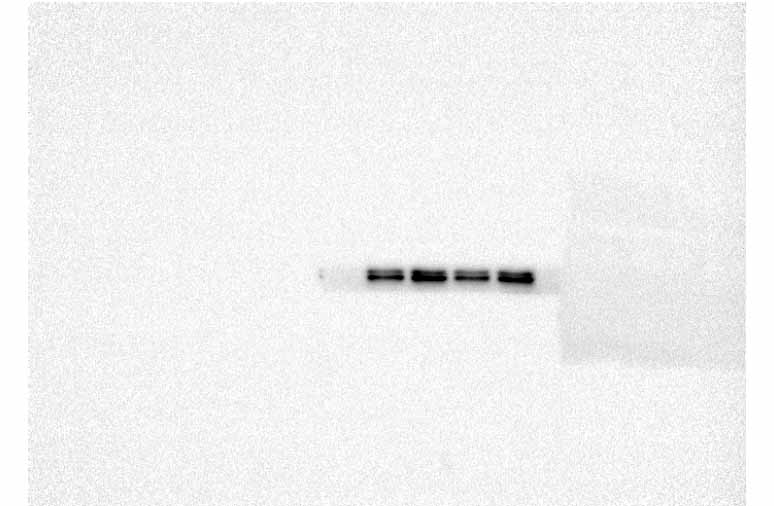

Supplement: Supplementary file 1 [file DataSheet1.ZIP › Raw Data/figureS1/WB/VEGF.jpg]
