# Supplementary figures and images for: Paeoniflorin Suppresses TBHP-Induced Oxidative Stress and Apoptosis in Human Umbilical Vein Endothelial Cells via the Nrf2/HO-1 Signaling Pathway and Improves Skin Flap Survival (part 1 of 2)
Source: Front Pharmacol. 2021 Nov 4;12:735530. doi: 10.3389/fphar.2021.735530 (PMC8600365; doi:10.3389/fphar.2021.735530)

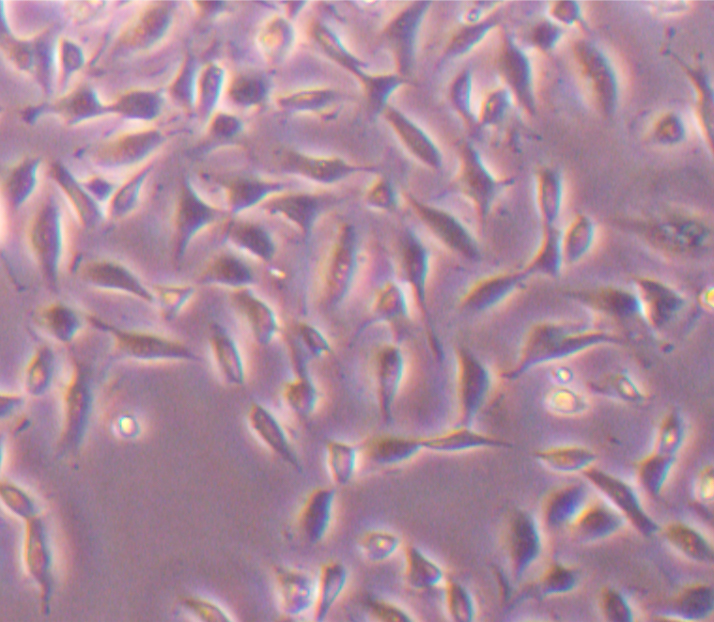

Supplement: Supplementary file 1 [file DataSheet1.ZIP › Raw Data/figure1/fig1 D/control.jpg]

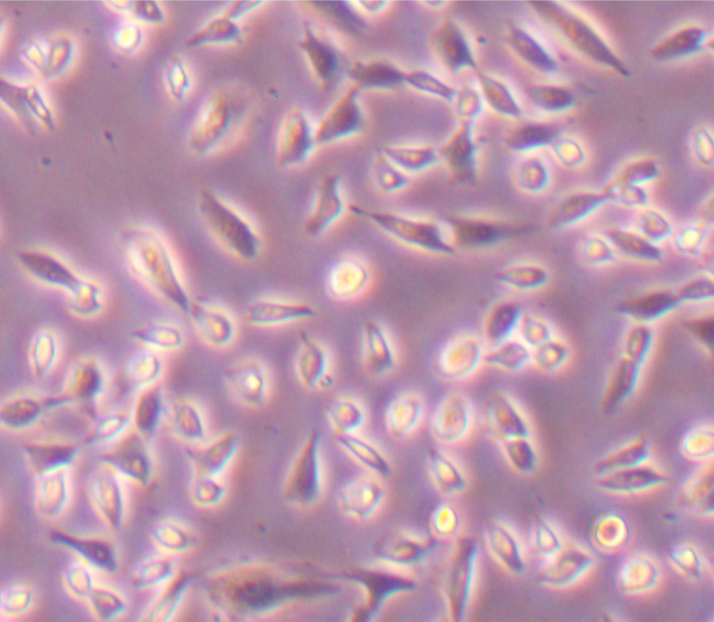

Supplement: Supplementary file 1 [file DataSheet1.ZIP › Raw Data/figure1/fig1 D/PF(0)+TBHP(500).jpg]

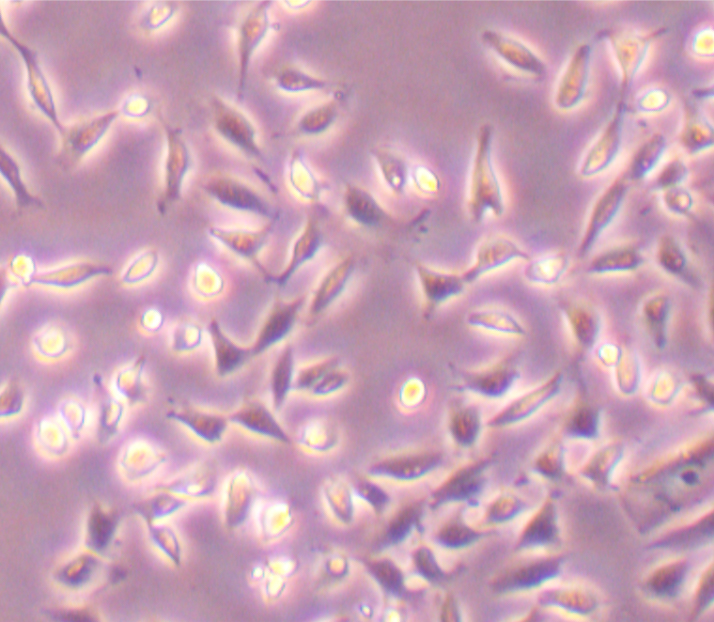

Supplement: Supplementary file 1 [file DataSheet1.ZIP › Raw Data/figure1/fig1 D/PF(10)+TBHP(500).jpg]

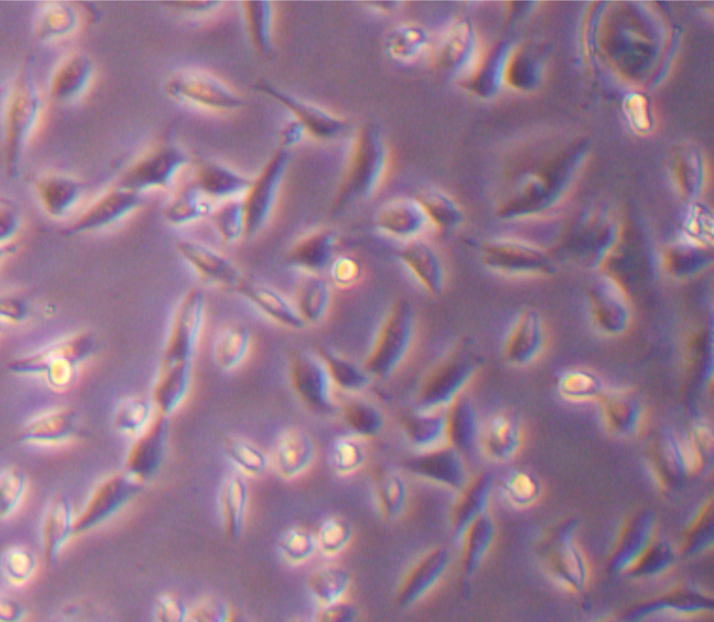

Supplement: Supplementary file 1 [file DataSheet1.ZIP › Raw Data/figure1/fig1 D/PF(20)+TBHP(500).jpg]

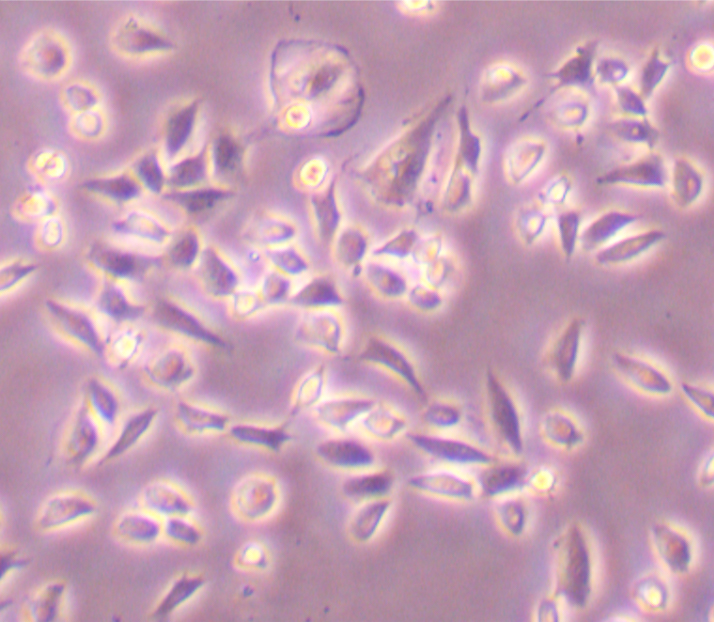

Supplement: Supplementary file 1 [file DataSheet1.ZIP › Raw Data/figure1/fig1 D/PF(5)+TBHP(500).jpg]

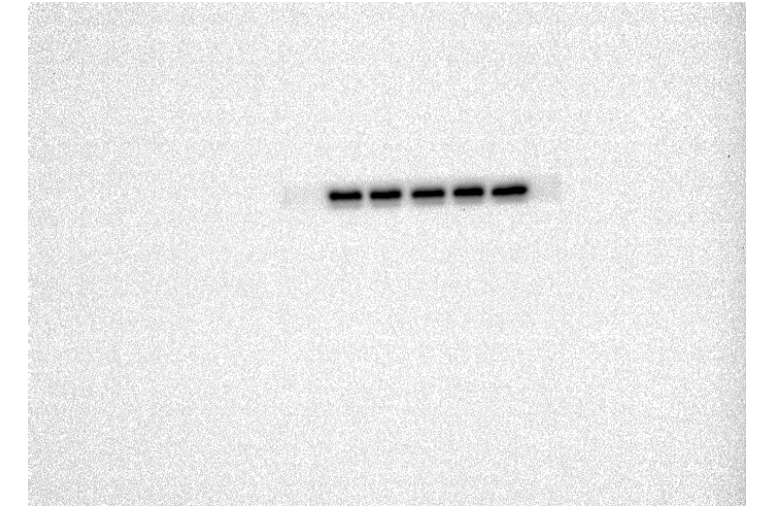

Supplement: Supplementary file 1 [file DataSheet1.ZIP › Raw Data/figure2/fig2 WB/B-actin.jpg]

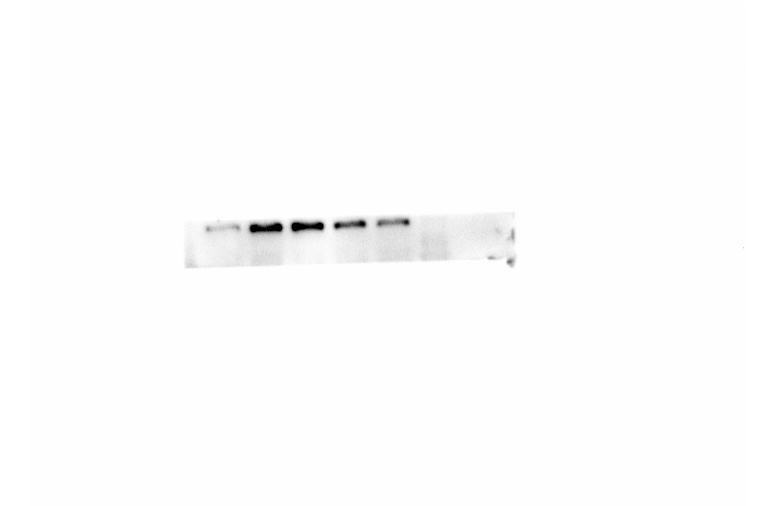

Supplement: Supplementary file 1 [file DataSheet1.ZIP › Raw Data/figure2/fig2 WB/Bax.jpg]

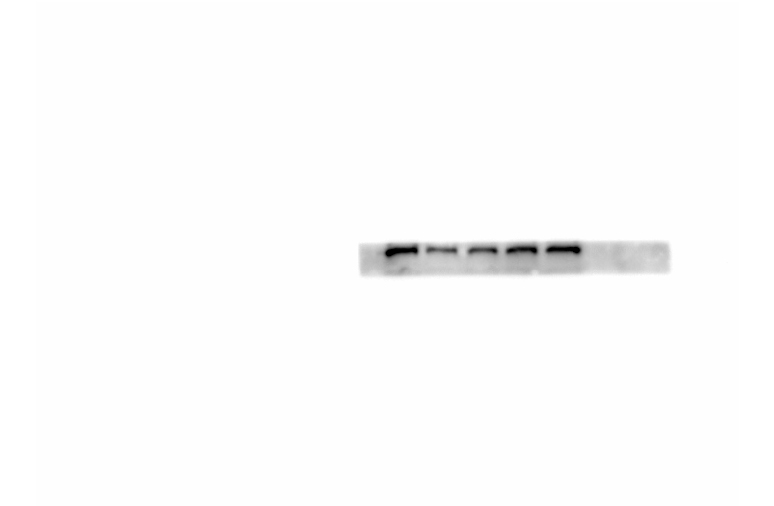

Supplement: Supplementary file 1 [file DataSheet1.ZIP › Raw Data/figure2/fig2 WB/Bcl-2.jpg]

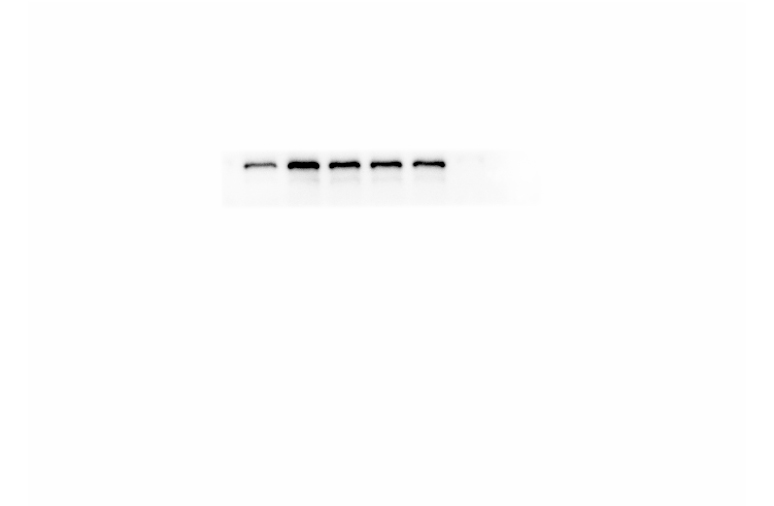

Supplement: Supplementary file 1 [file DataSheet1.ZIP › Raw Data/figure2/fig2 WB/cleaved cas3.jpg]

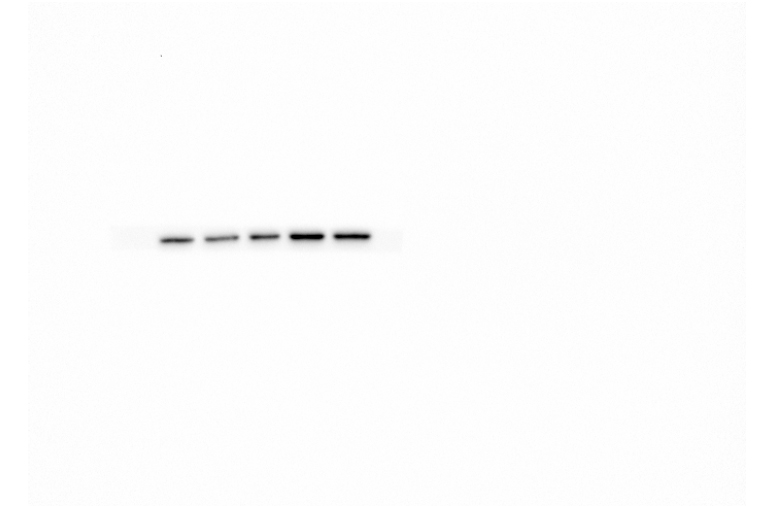

Supplement: Supplementary file 1 [file DataSheet1.ZIP › Raw Data/figure2/fig2 WB/PRAR.jpg]

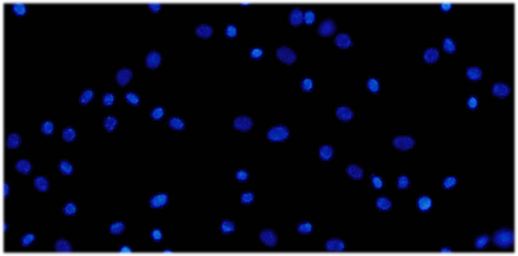

Supplement: Supplementary file 1 [file DataSheet1.ZIP › Raw Data/figure2/Tunel/control DAPI.jpg]

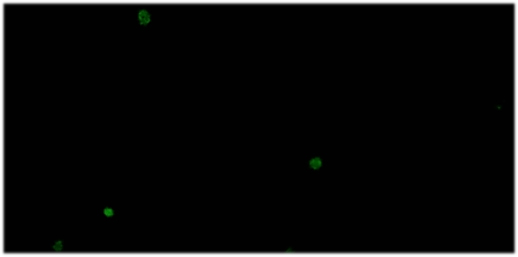

Supplement: Supplementary file 1 [file DataSheet1.ZIP › Raw Data/figure2/Tunel/control.jpg]

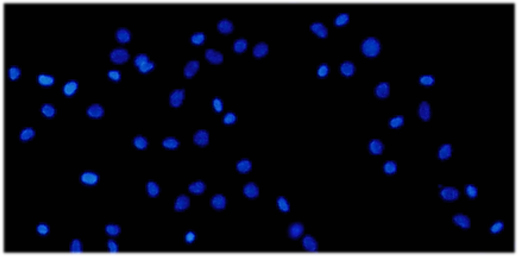

Supplement: Supplementary file 1 [file DataSheet1.ZIP › Raw Data/figure2/Tunel/PF(0) DAPI.jpg]

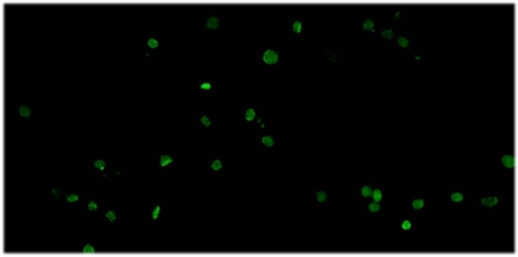

Supplement: Supplementary file 1 [file DataSheet1.ZIP › Raw Data/figure2/Tunel/PF(0)+TBHP(500).jpg]

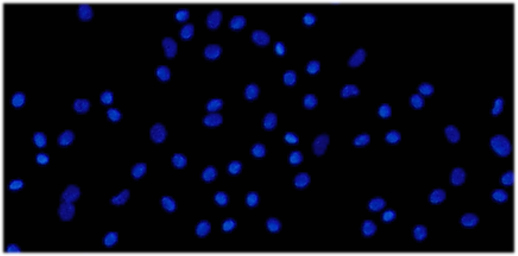

Supplement: Supplementary file 1 [file DataSheet1.ZIP › Raw Data/figure2/Tunel/PF(10) DAPI.jpg]

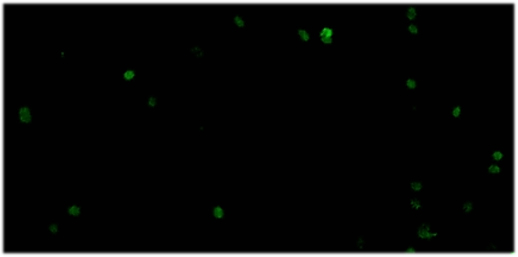

Supplement: Supplementary file 1 [file DataSheet1.ZIP › Raw Data/figure2/Tunel/PF(10).jpg]

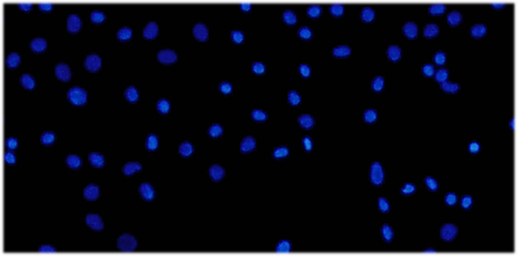

Supplement: Supplementary file 1 [file DataSheet1.ZIP › Raw Data/figure2/Tunel/PF(20) DAPI.jpg]

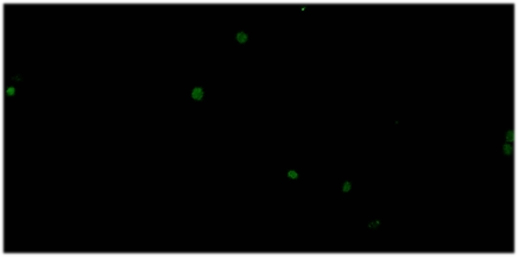

Supplement: Supplementary file 1 [file DataSheet1.ZIP › Raw Data/figure2/Tunel/PF(20).jpg]

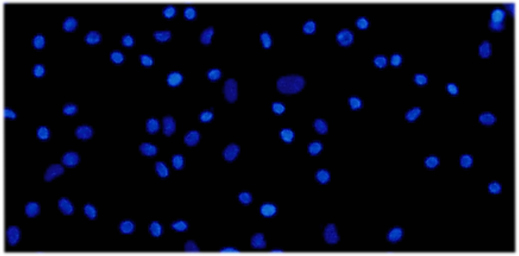

Supplement: Supplementary file 1 [file DataSheet1.ZIP › Raw Data/figure2/Tunel/PF(5) DAPI.jpg]

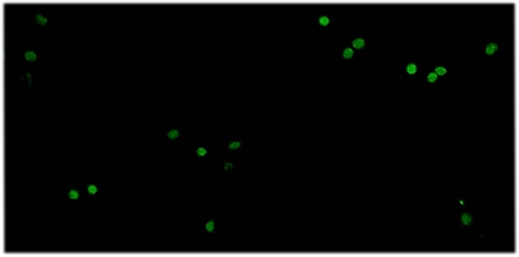

Supplement: Supplementary file 1 [file DataSheet1.ZIP › Raw Data/figure2/Tunel/PF(5)+TBHP(500).jpg]

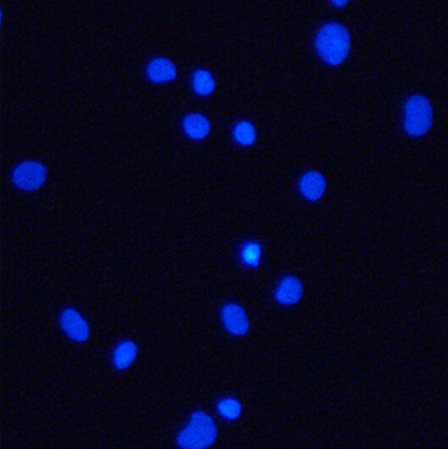

Supplement: Supplementary file 1 [file DataSheet1.ZIP › Raw Data/figure3/fig3 C,D/Hoechst control.jpg]

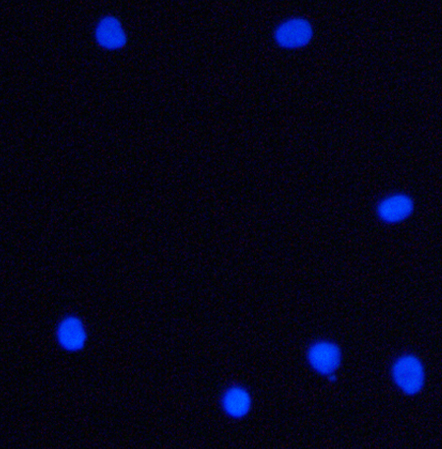

Supplement: Supplementary file 1 [file DataSheet1.ZIP › Raw Data/figure3/fig3 C,D/Hoechst TBHP+PF.jpg]

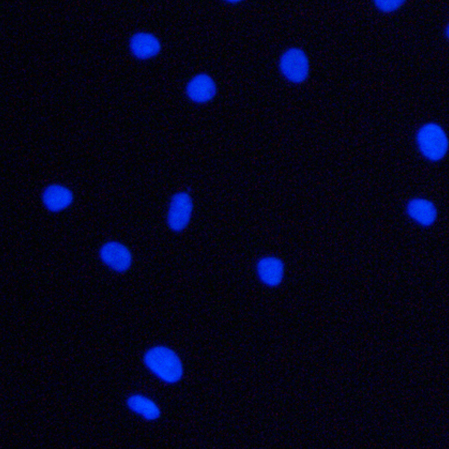

Supplement: Supplementary file 1 [file DataSheet1.ZIP › Raw Data/figure3/fig3 C,D/Hoechst TBHP.jpg]

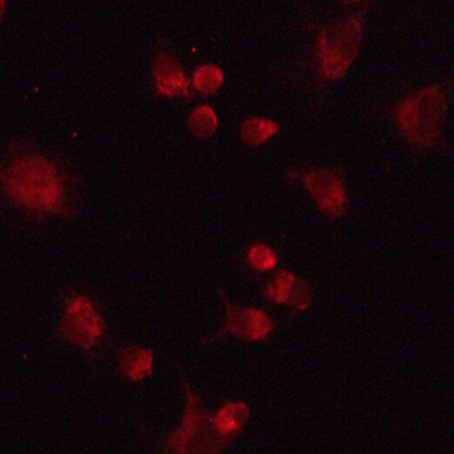

Supplement: Supplementary file 1 [file DataSheet1.ZIP › Raw Data/figure3/fig3 C,D/Mito Tracker control.jpg]

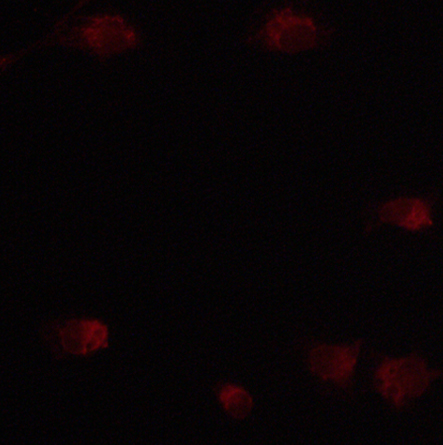

Supplement: Supplementary file 1 [file DataSheet1.ZIP › Raw Data/figure3/fig3 C,D/Mito Tracker TBHP+PF.jpg]

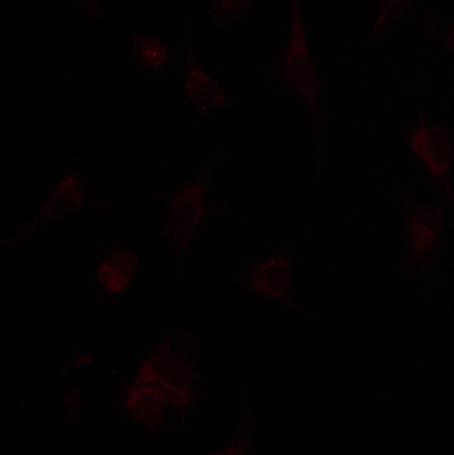

Supplement: Supplementary file 1 [file DataSheet1.ZIP › Raw Data/figure3/fig3 C,D/Mito Tracker TBHP.jpg]

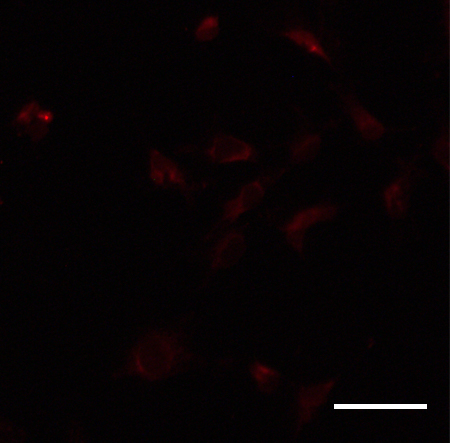

Supplement: Supplementary file 1 [file DataSheet1.ZIP › Raw Data/figure3/fig3 C,D/Mitosox control.jpg]

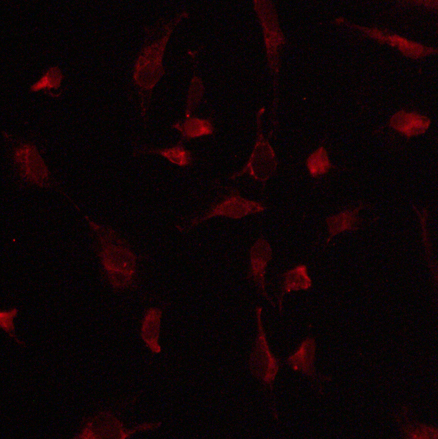

Supplement: Supplementary file 1 [file DataSheet1.ZIP › Raw Data/figure3/fig3 C,D/Mitosox TBHP+PF.jpg]

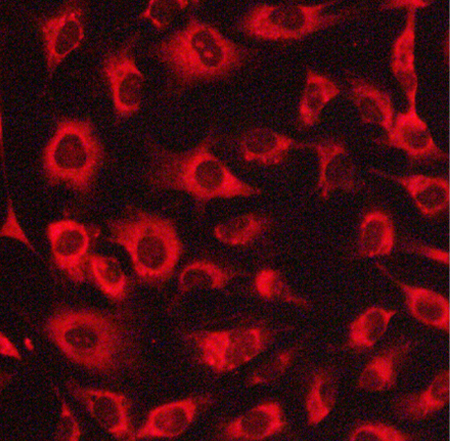

Supplement: Supplementary file 1 [file DataSheet1.ZIP › Raw Data/figure3/fig3 C,D/Mitosox TBHP.jpg]

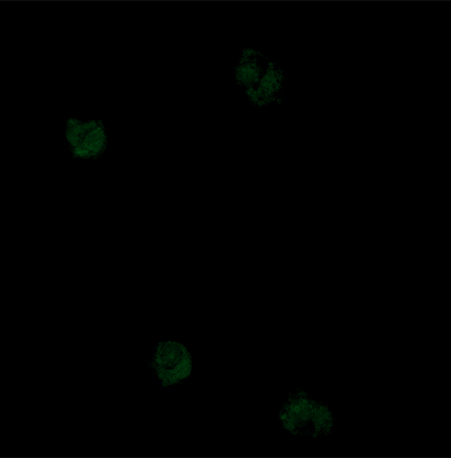

Supplement: Supplementary file 1 [file DataSheet1.ZIP › Raw Data/figure3/fig3 E/JC 1.1.jpg]

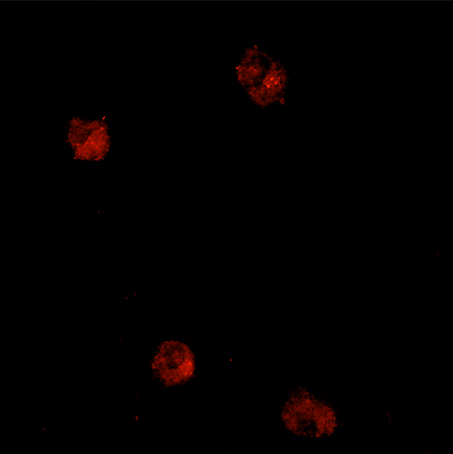

Supplement: Supplementary file 1 [file DataSheet1.ZIP › Raw Data/figure3/fig3 E/JC 1.jpg]

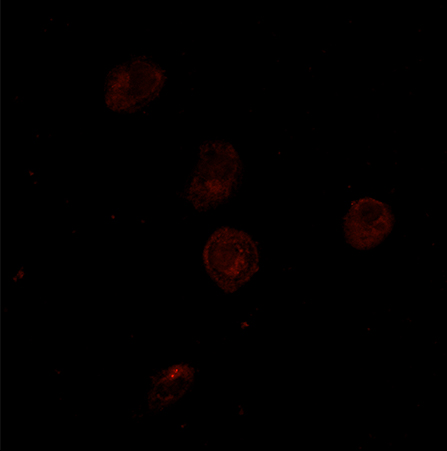

Supplement: Supplementary file 1 [file DataSheet1.ZIP › Raw Data/figure3/fig3 E/JC 2.0.jpg]

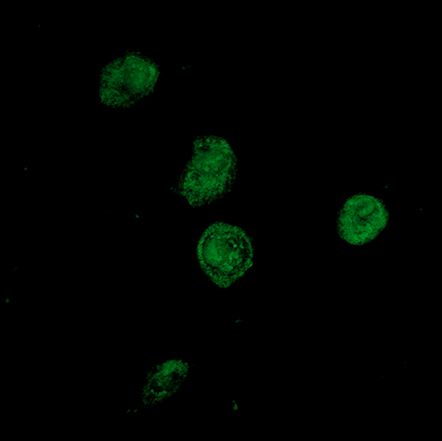

Supplement: Supplementary file 1 [file DataSheet1.ZIP › Raw Data/figure3/fig3 E/JC 2.1.jpg]

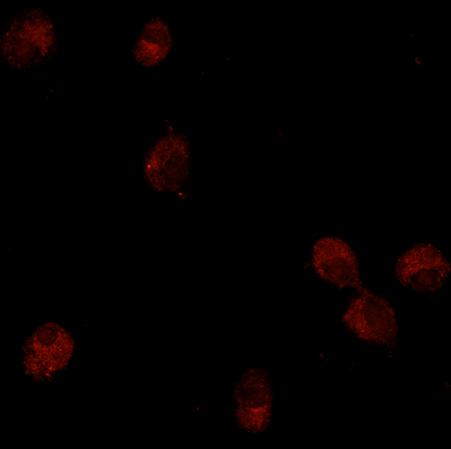

Supplement: Supplementary file 1 [file DataSheet1.ZIP › Raw Data/figure3/fig3 E/JC 3.0.jpg]

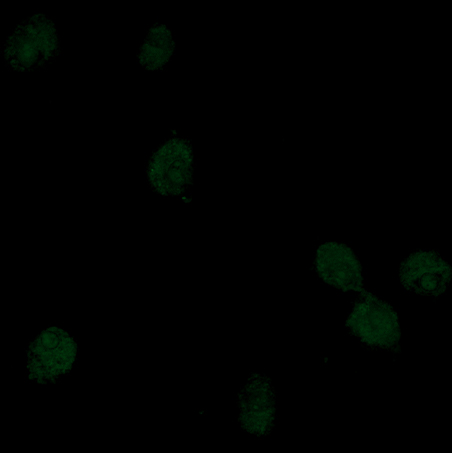

Supplement: Supplementary file 1 [file DataSheet1.ZIP › Raw Data/figure3/fig3 E/JC 3.1.jpg]

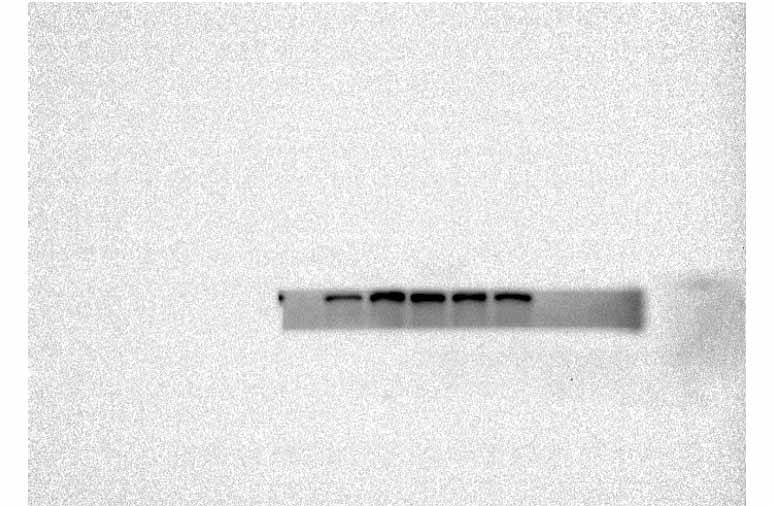

Supplement: Supplementary file 1 [file DataSheet1.ZIP › Raw Data/figure3/fig3 WB/cytoplasmic cytochrome C.jpg]

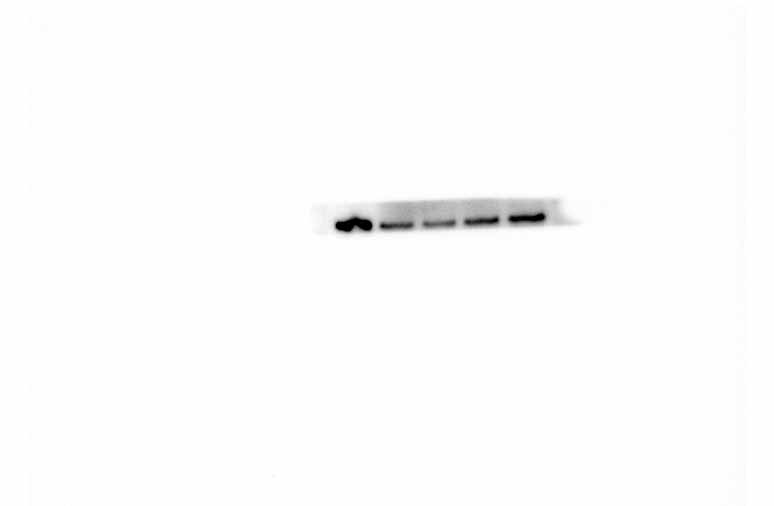

Supplement: Supplementary file 1 [file DataSheet1.ZIP › Raw Data/figure3/fig3 WB/mitochondria cytochrome C.jpg]

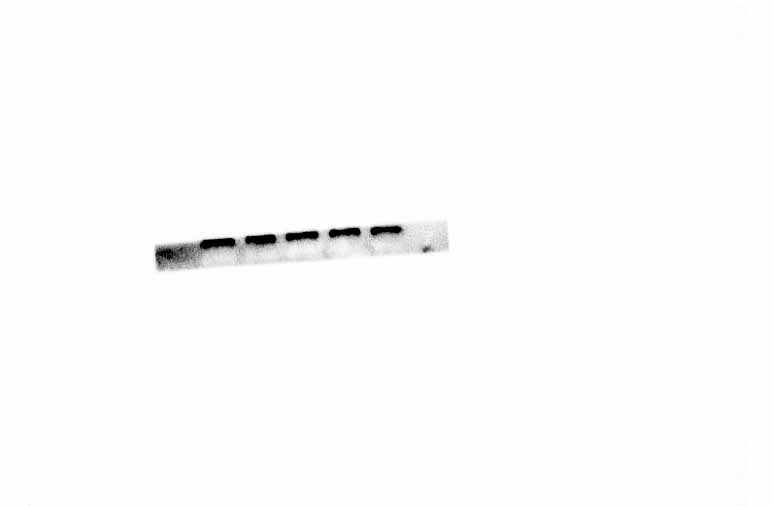

Supplement: Supplementary file 1 [file DataSheet1.ZIP › Raw Data/figure3/fig3 WB/β-actin(1).jpg]

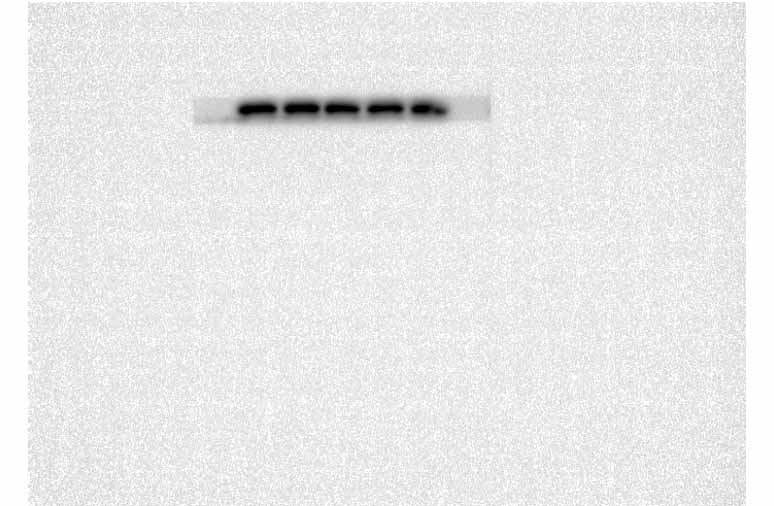

Supplement: Supplementary file 1 [file DataSheet1.ZIP › Raw Data/figure3/fig3 WB/β-actin.jpg]

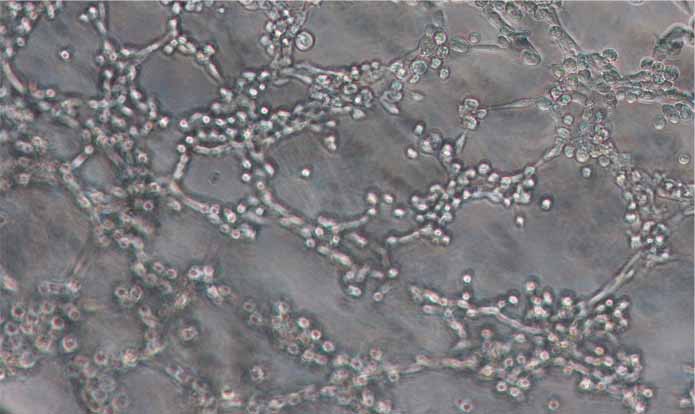

Supplement: Supplementary file 1 [file DataSheet1.ZIP › Raw Data/figure4/fig4 A/1.jpg]

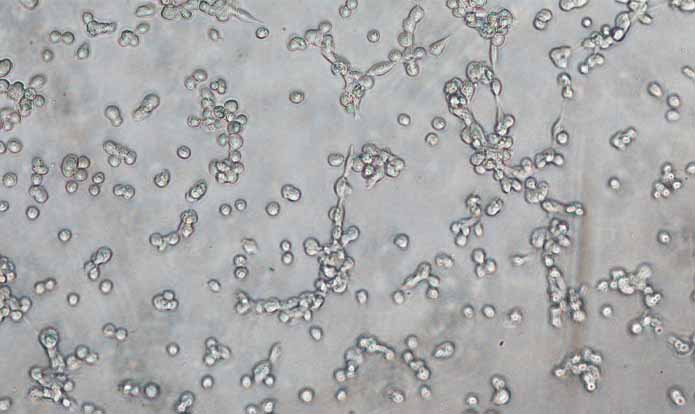

Supplement: Supplementary file 1 [file DataSheet1.ZIP › Raw Data/figure4/fig4 A/2.jpg]

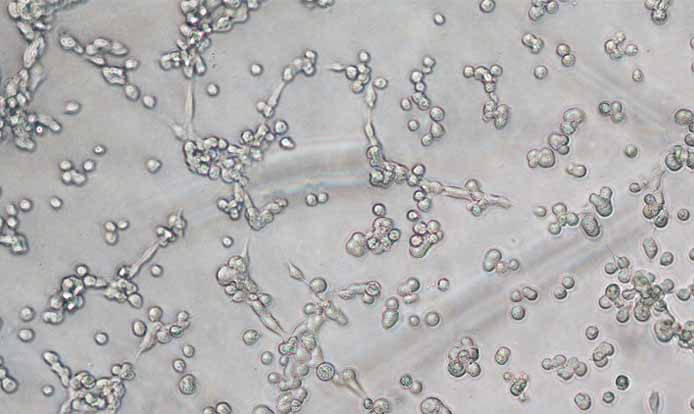

Supplement: Supplementary file 1 [file DataSheet1.ZIP › Raw Data/figure4/fig4 A/3.jpg]

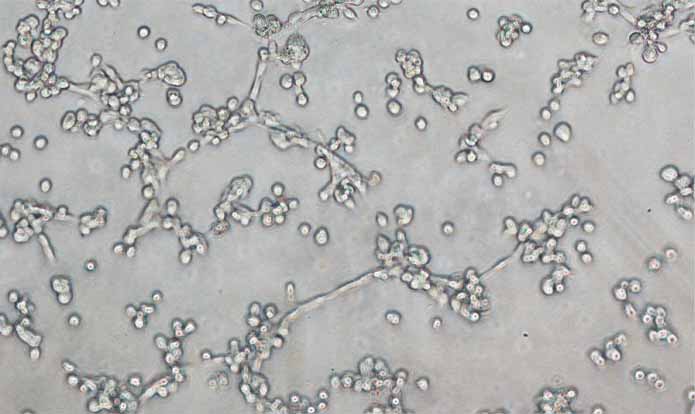

Supplement: Supplementary file 1 [file DataSheet1.ZIP › Raw Data/figure4/fig4 A/4.jpg]

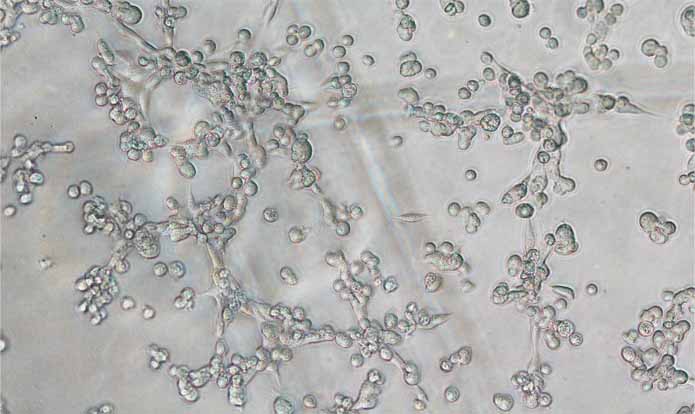

Supplement: Supplementary file 1 [file DataSheet1.ZIP › Raw Data/figure4/fig4 A/5.jpg]

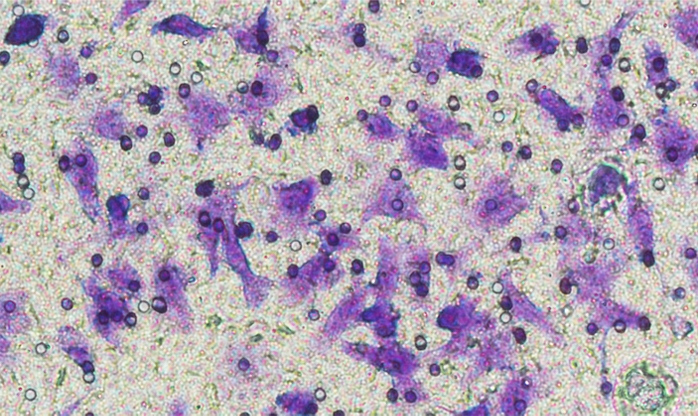

Supplement: Supplementary file 1 [file DataSheet1.ZIP › Raw Data/figure4/fig4 B/1.jpg]

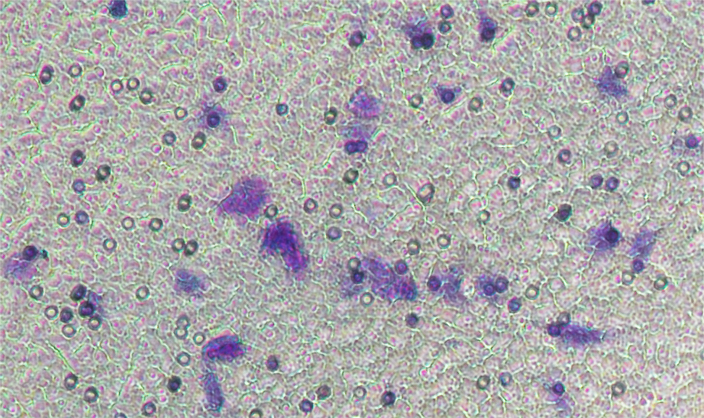

Supplement: Supplementary file 1 [file DataSheet1.ZIP › Raw Data/figure4/fig4 B/2.jpg]

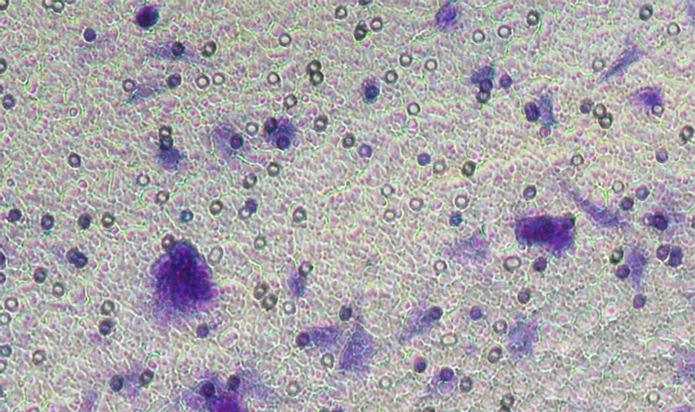

Supplement: Supplementary file 1 [file DataSheet1.ZIP › Raw Data/figure4/fig4 B/3.jpg]

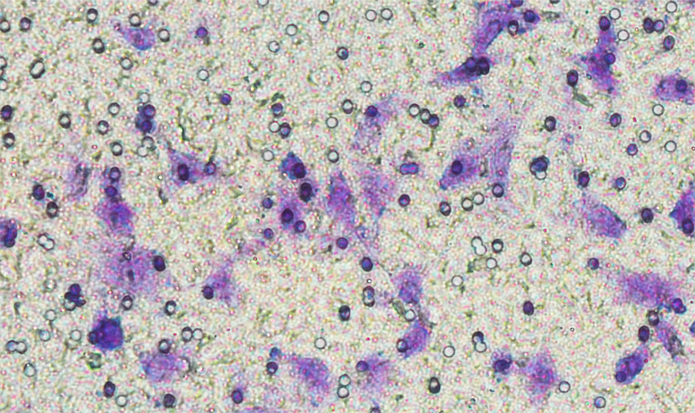

Supplement: Supplementary file 1 [file DataSheet1.ZIP › Raw Data/figure4/fig4 B/4.jpg]

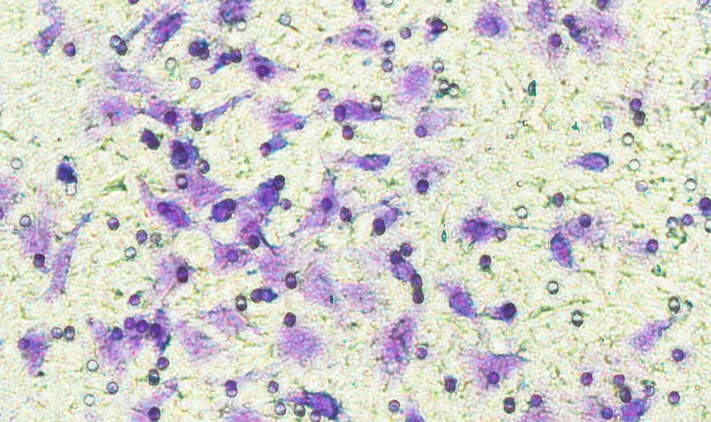

Supplement: Supplementary file 1 [file DataSheet1.ZIP › Raw Data/figure4/fig4 B/5.jpg]

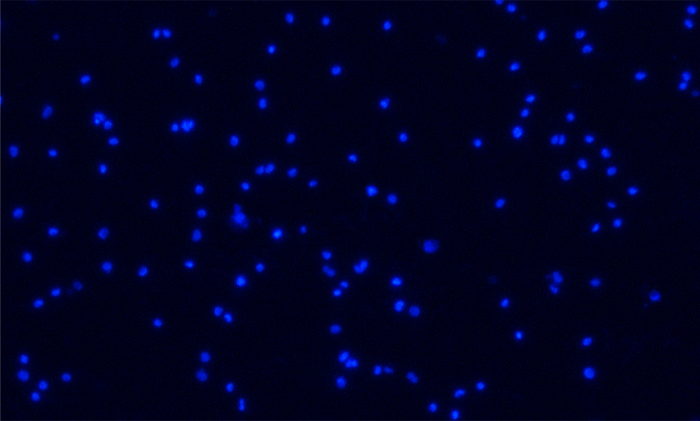

Supplement: Supplementary file 1 [file DataSheet1.ZIP › Raw Data/figure4/fig4 C/1.jpg]

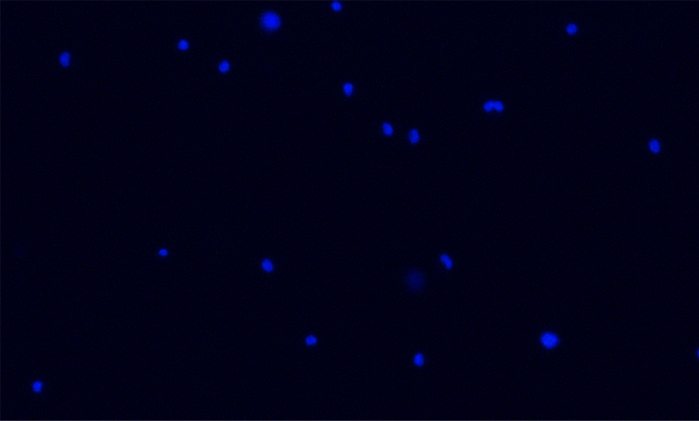

Supplement: Supplementary file 1 [file DataSheet1.ZIP › Raw Data/figure4/fig4 C/2.jpg]

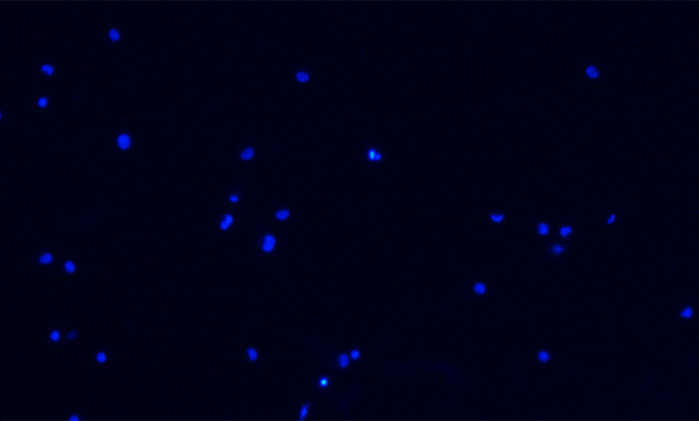

Supplement: Supplementary file 1 [file DataSheet1.ZIP › Raw Data/figure4/fig4 C/3.jpg]

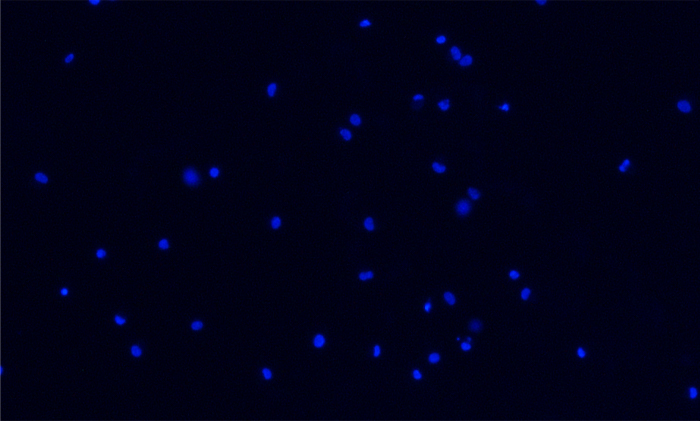

Supplement: Supplementary file 1 [file DataSheet1.ZIP › Raw Data/figure4/fig4 C/4.jpg]

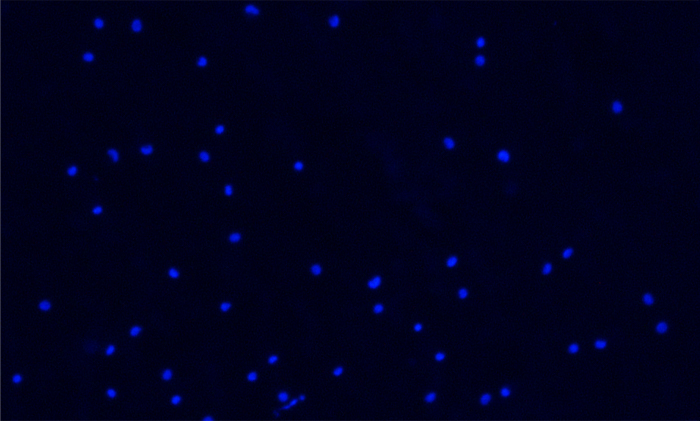

Supplement: Supplementary file 1 [file DataSheet1.ZIP › Raw Data/figure4/fig4 C/5.jpg]

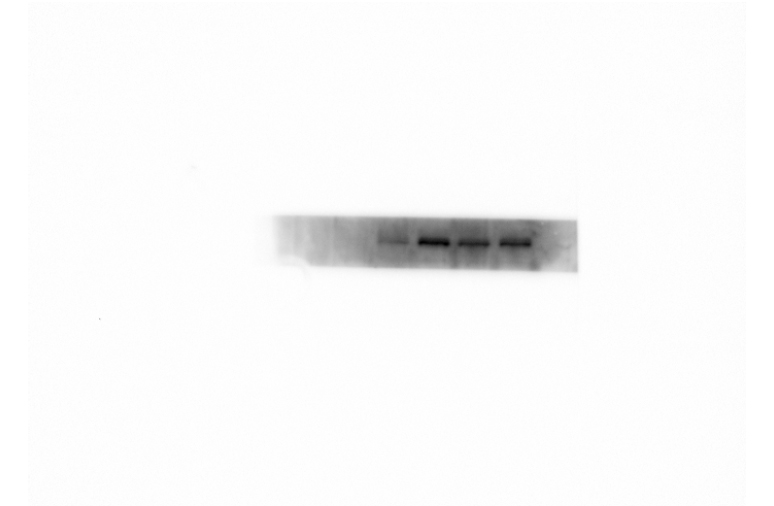

Supplement: Supplementary file 1 [file DataSheet1.ZIP › Raw Data/figure5/fig5 WB/Bax.jpg]

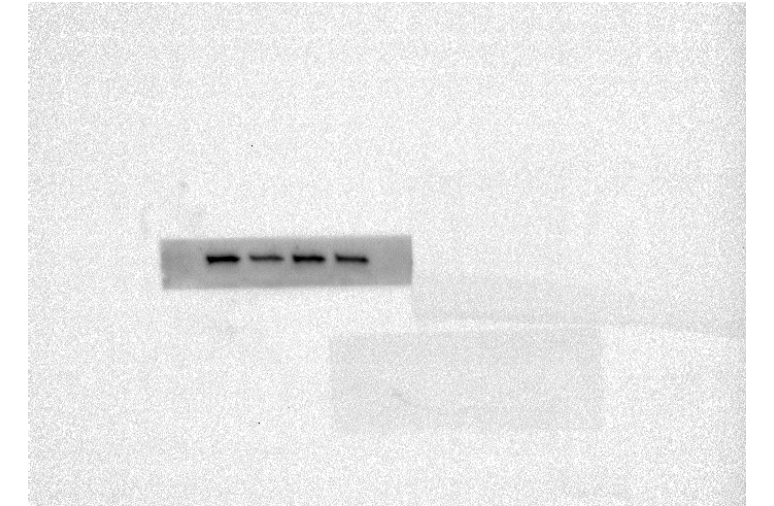

Supplement: Supplementary file 1 [file DataSheet1.ZIP › Raw Data/figure5/fig5 WB/Bcl-2.jpg]

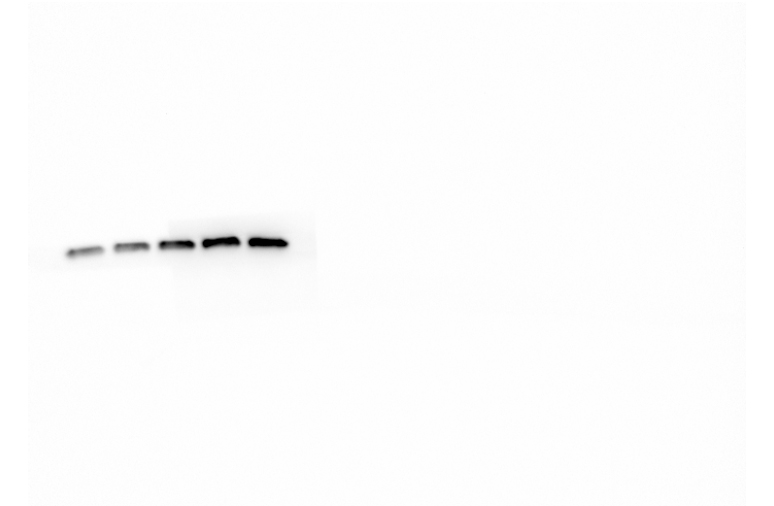

Supplement: Supplementary file 1 [file DataSheet1.ZIP › Raw Data/figure5/fig5 WB/HO-1 a.jpg]

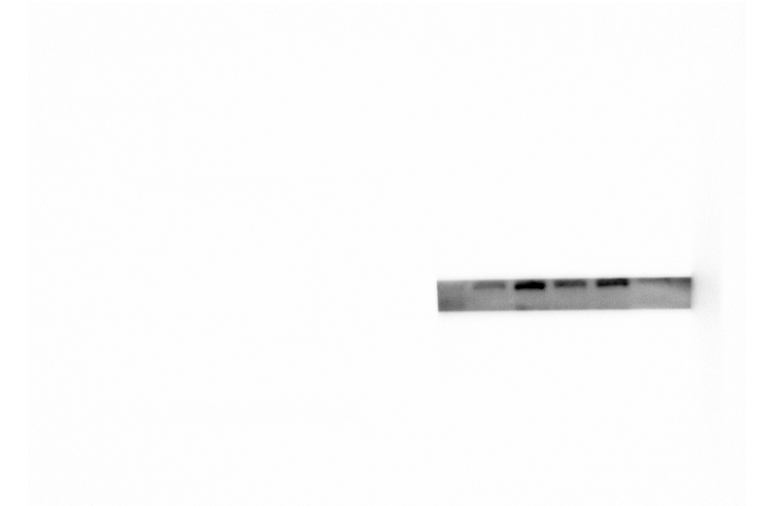

Supplement: Supplementary file 1 [file DataSheet1.ZIP › Raw Data/figure5/fig5 WB/HO-1 b.jpg]

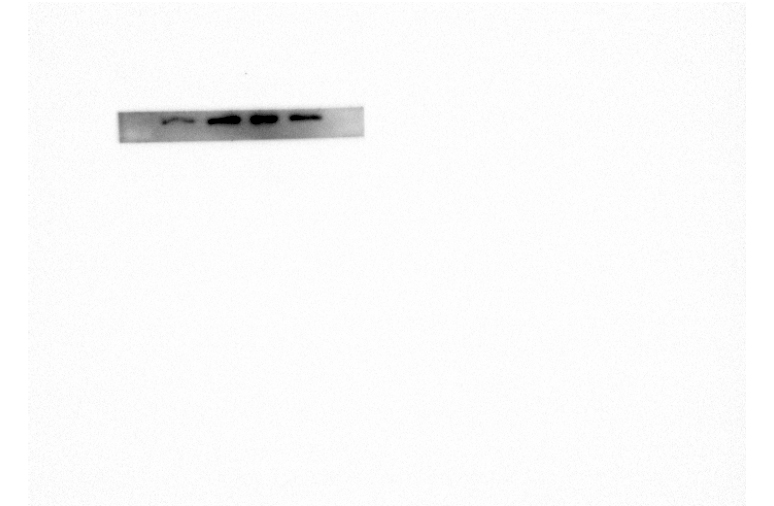

Supplement: Supplementary file 1 [file DataSheet1.ZIP › Raw Data/figure5/fig5 WB/HO-1 c.jpg]

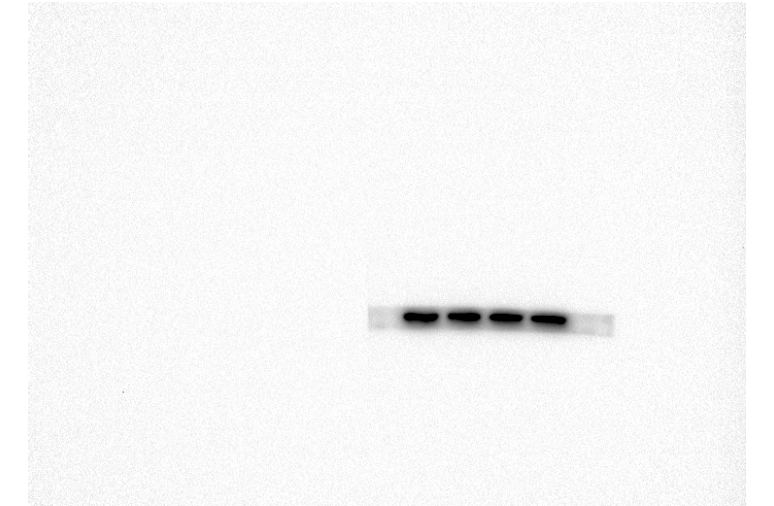

Supplement: Supplementary file 1 [file DataSheet1.ZIP › Raw Data/figure5/fig5 WB/β-actin.jpg]

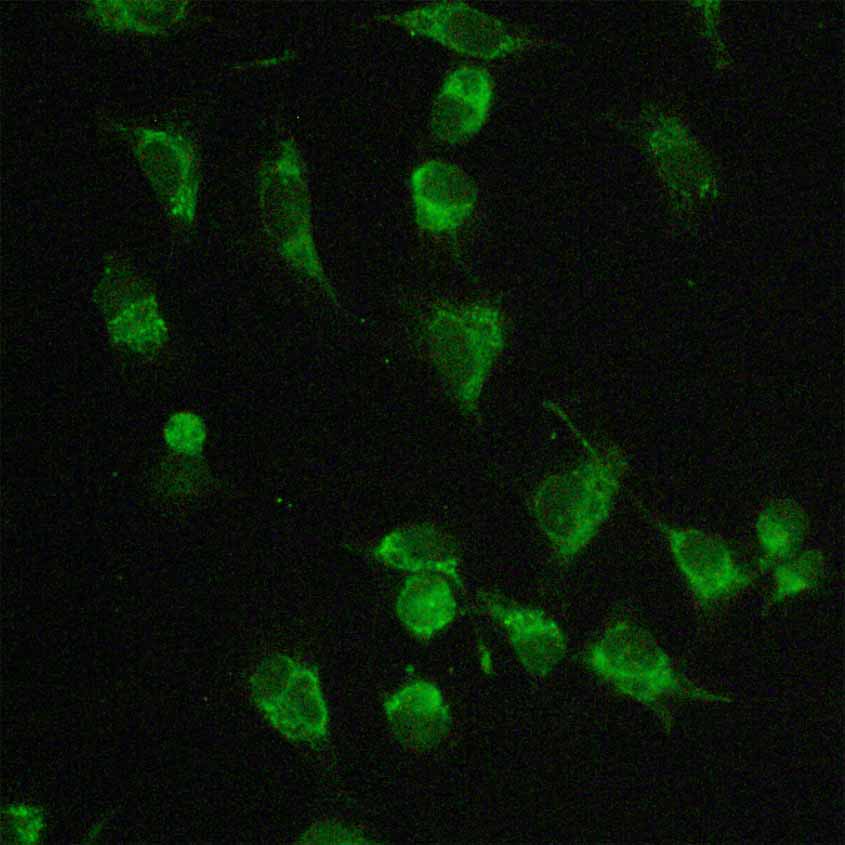

Supplement: Supplementary file 1 [file DataSheet1.ZIP › Raw Data/figure5/fig5.C/1.jpg]

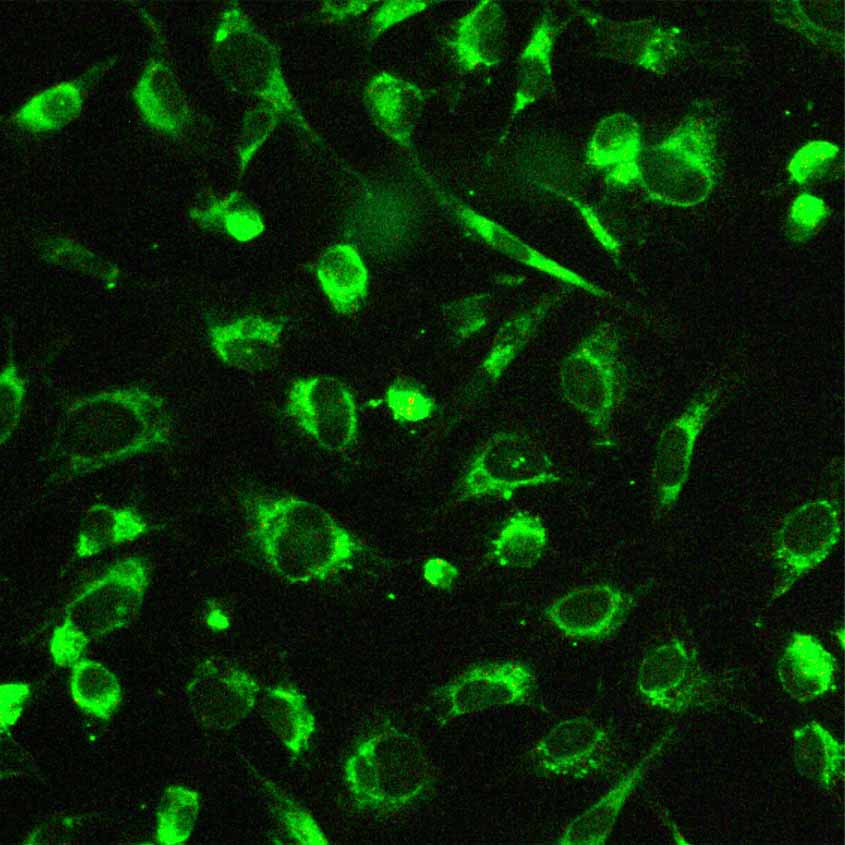

Supplement: Supplementary file 1 [file DataSheet1.ZIP › Raw Data/figure5/fig5.C/2.jpg]

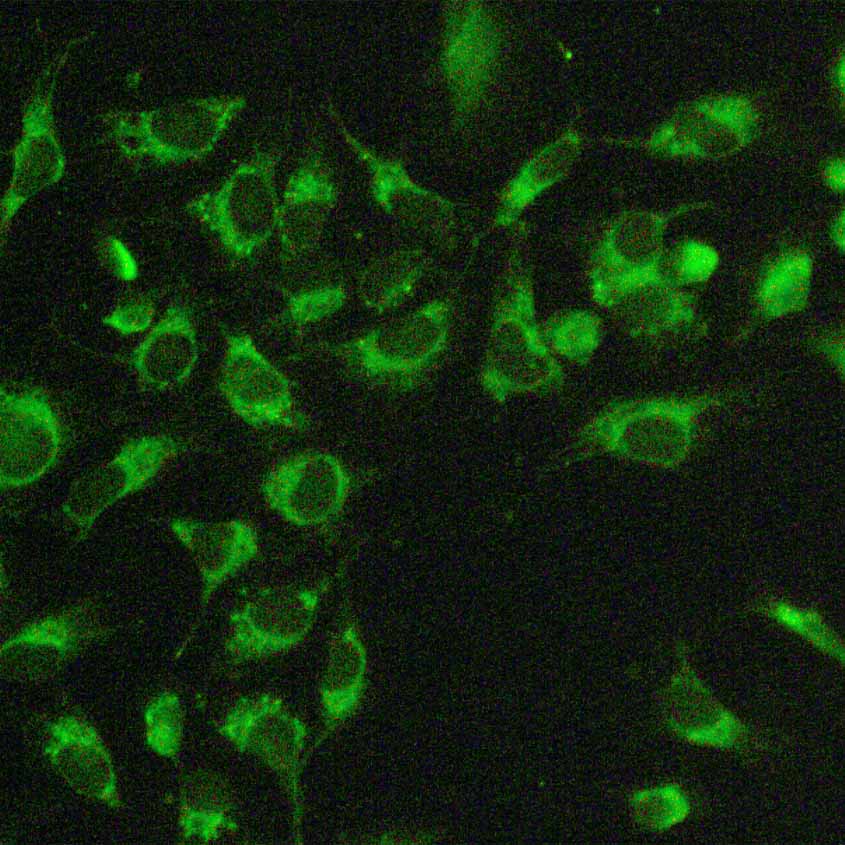

Supplement: Supplementary file 1 [file DataSheet1.ZIP › Raw Data/figure5/fig5.C/3.jpg]

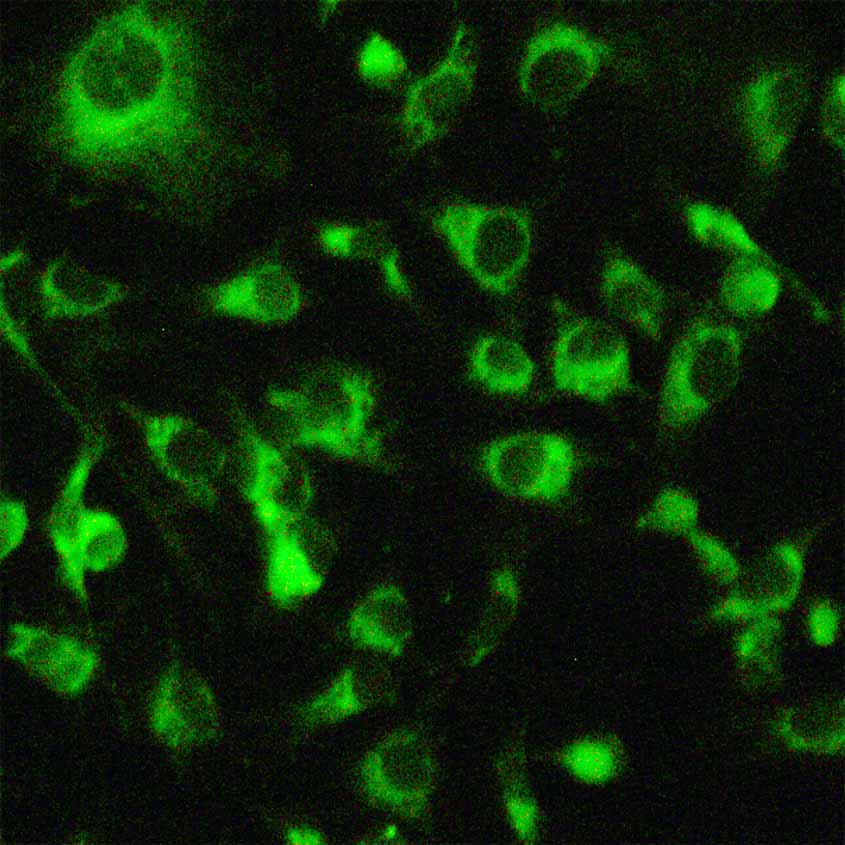

Supplement: Supplementary file 1 [file DataSheet1.ZIP › Raw Data/figure5/fig5.C/4.jpg]

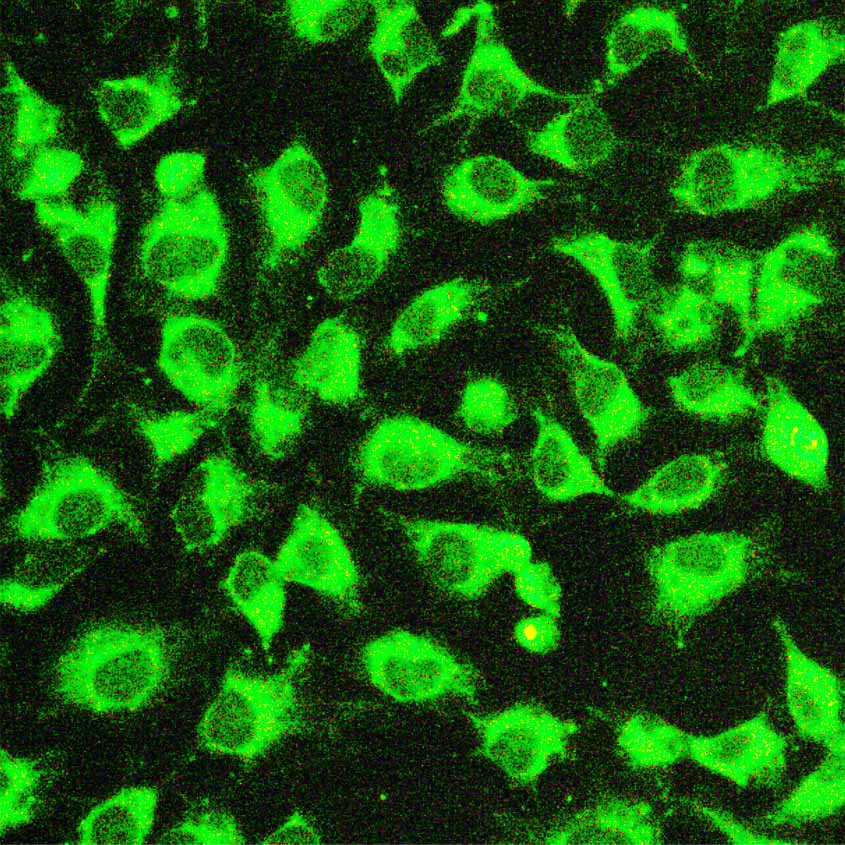

Supplement: Supplementary file 1 [file DataSheet1.ZIP › Raw Data/figure5/fig5.C/5.jpg]

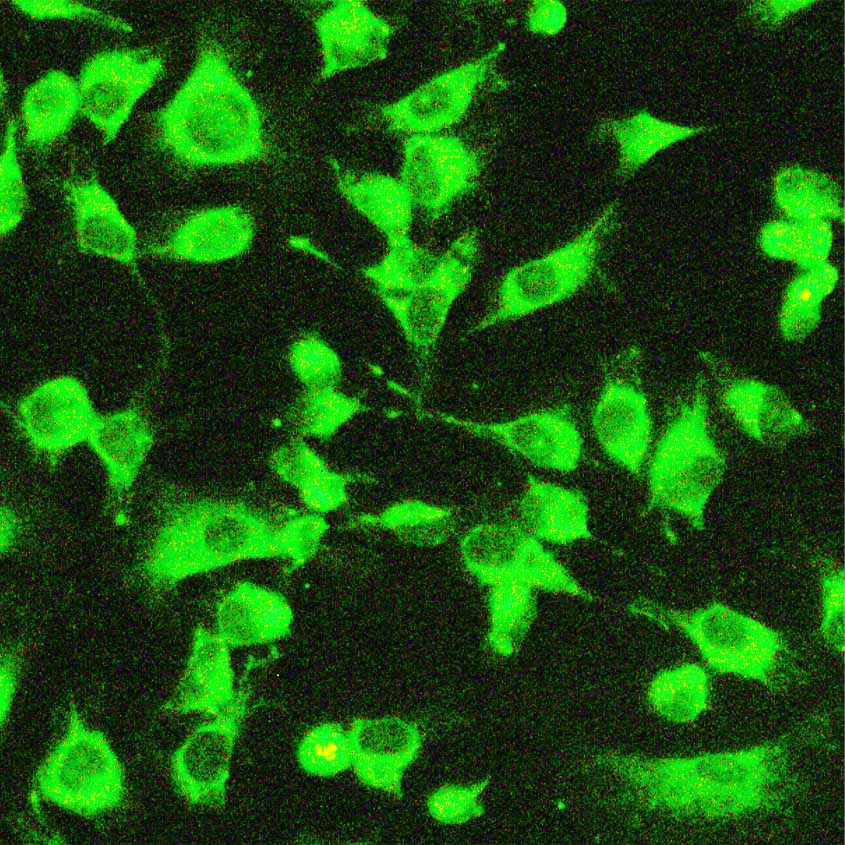

Supplement: Supplementary file 1 [file DataSheet1.ZIP › Raw Data/figure5/fig5.C/6.jpg]

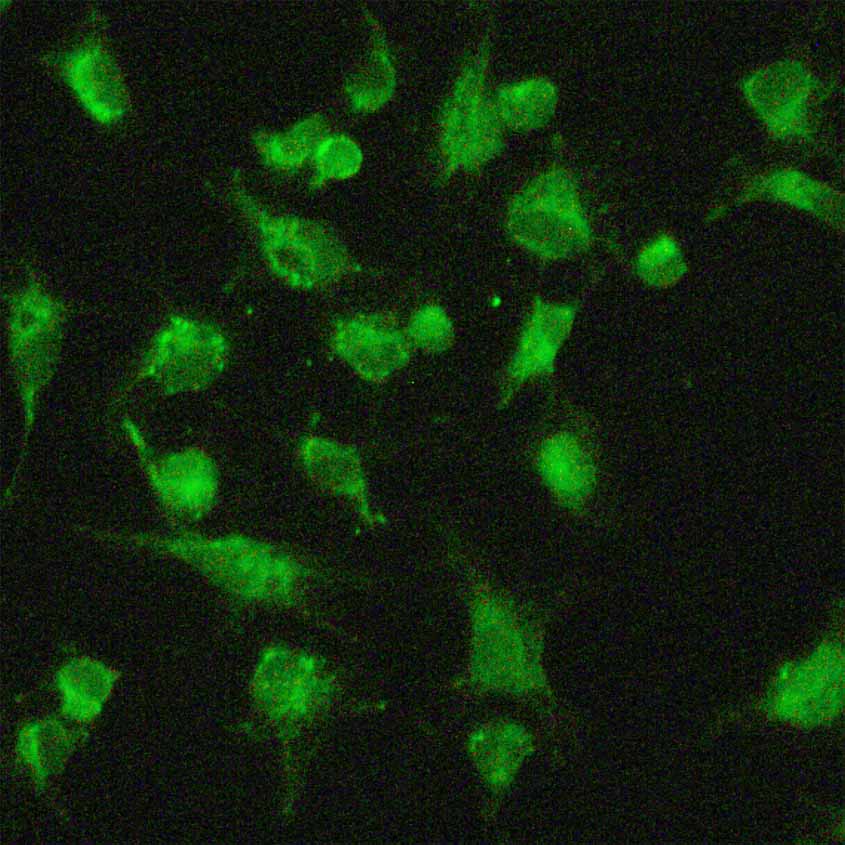

Supplement: Supplementary file 1 [file DataSheet1.ZIP › Raw Data/figure5/fig5.C/7.jpg]

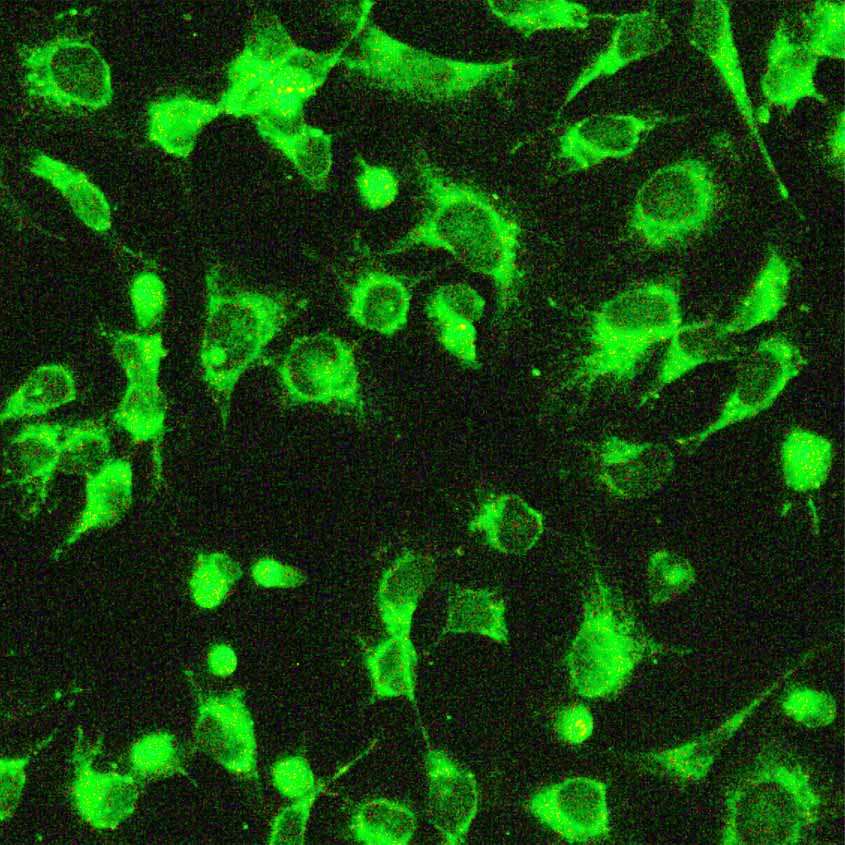

Supplement: Supplementary file 1 [file DataSheet1.ZIP › Raw Data/figure5/fig5.C/8.jpg]

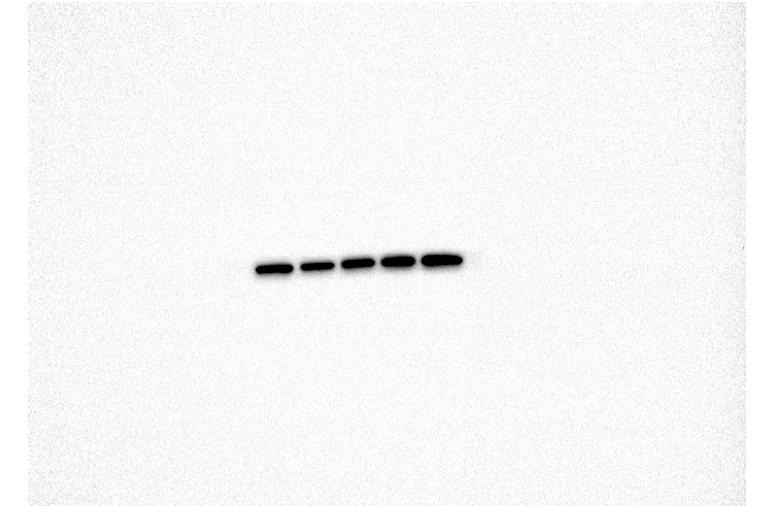

Supplement: Supplementary file 1 [file DataSheet1.ZIP › Raw Data/figure6/fig6. WB/B-actin.jpg]

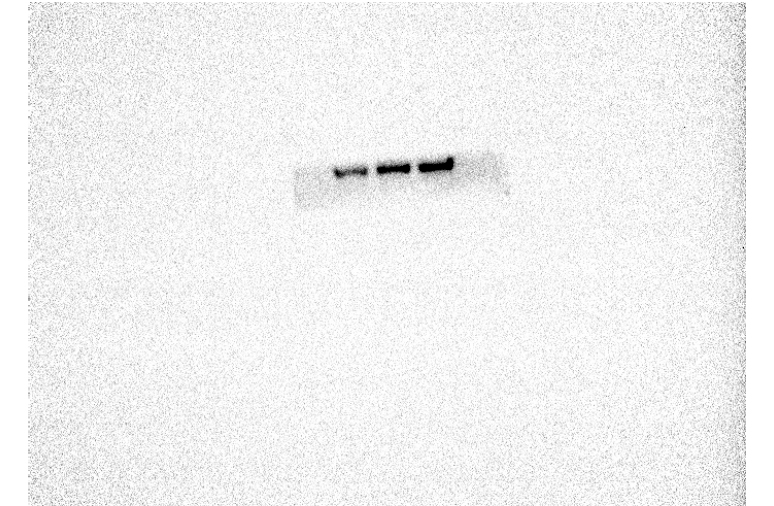

Supplement: Supplementary file 1 [file DataSheet1.ZIP › Raw Data/figure6/fig6. WB/Bax.jpg]

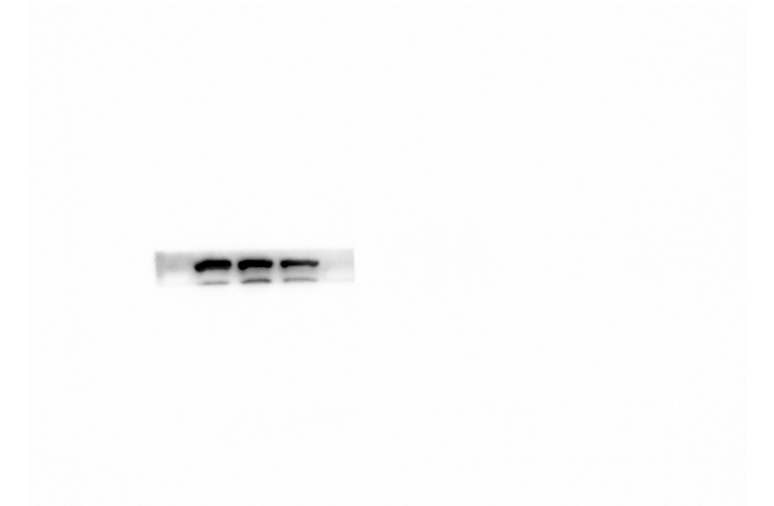

Supplement: Supplementary file 1 [file DataSheet1.ZIP › Raw Data/figure6/fig6. WB/Bcl-2.jpg]

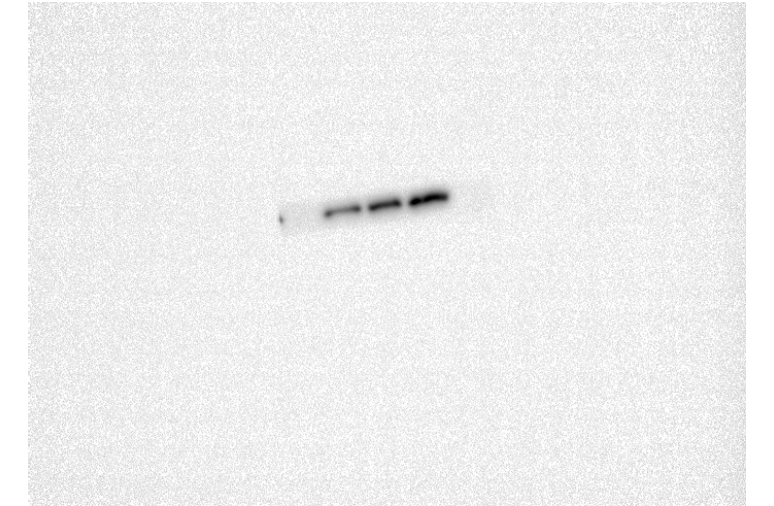

Supplement: Supplementary file 1 [file DataSheet1.ZIP › Raw Data/figure6/fig6. WB/C-cas3.jpg]

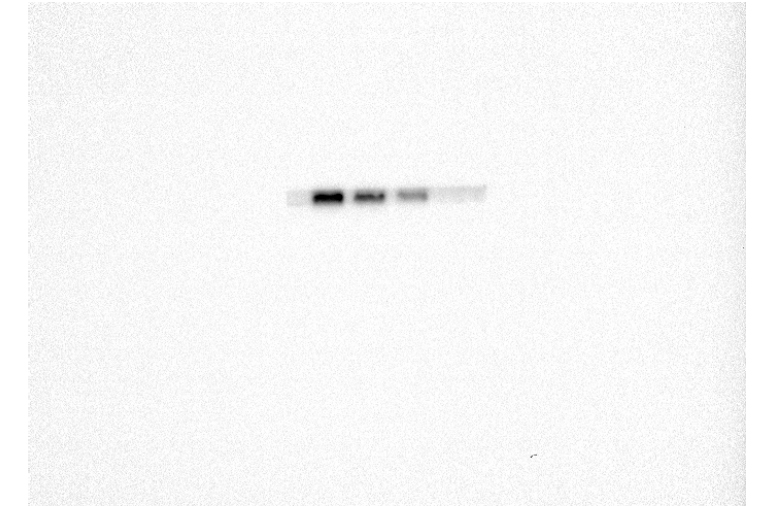

Supplement: Supplementary file 1 [file DataSheet1.ZIP › Raw Data/figure6/fig6. WB/HO-1.jpg]

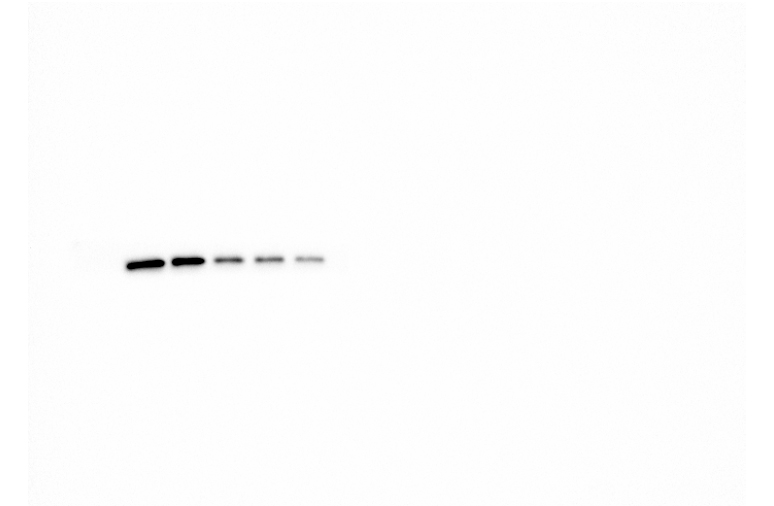

Supplement: Supplementary file 1 [file DataSheet1.ZIP › Raw Data/figure6/fig6. WB/Keap1.jpg]

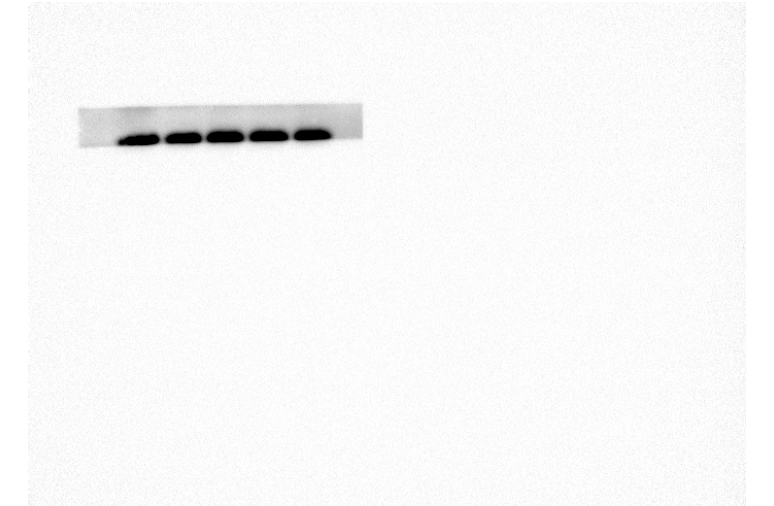

Supplement: Supplementary file 1 [file DataSheet1.ZIP › Raw Data/figure6/fig6. WB/LaminB 1.jpg]

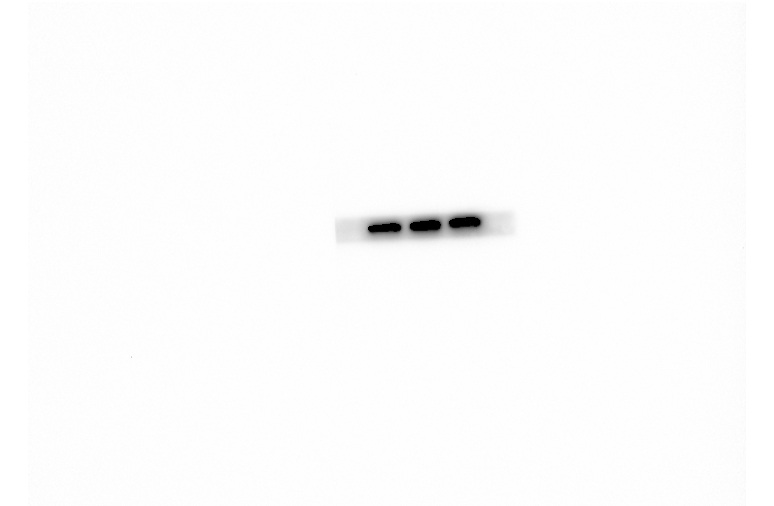

Supplement: Supplementary file 1 [file DataSheet1.ZIP › Raw Data/figure6/fig6. WB/LaminB 2.jpg]

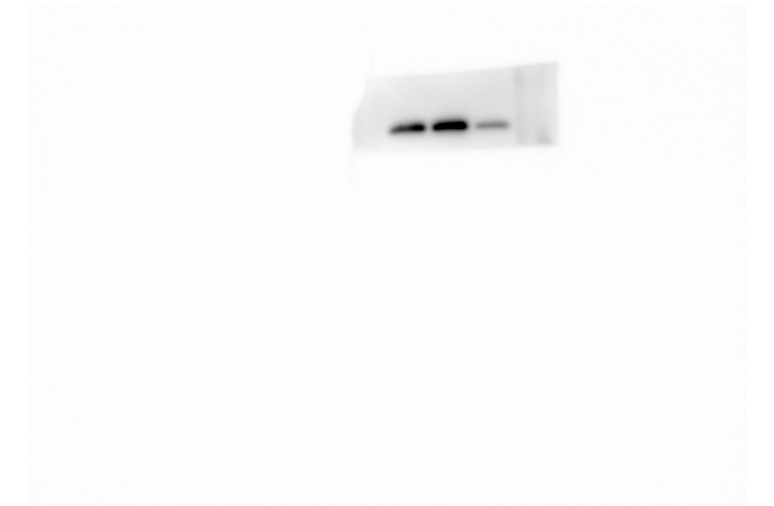

Supplement: Supplementary file 1 [file DataSheet1.ZIP › Raw Data/figure6/fig6. WB/Nrf2 2.jpg]

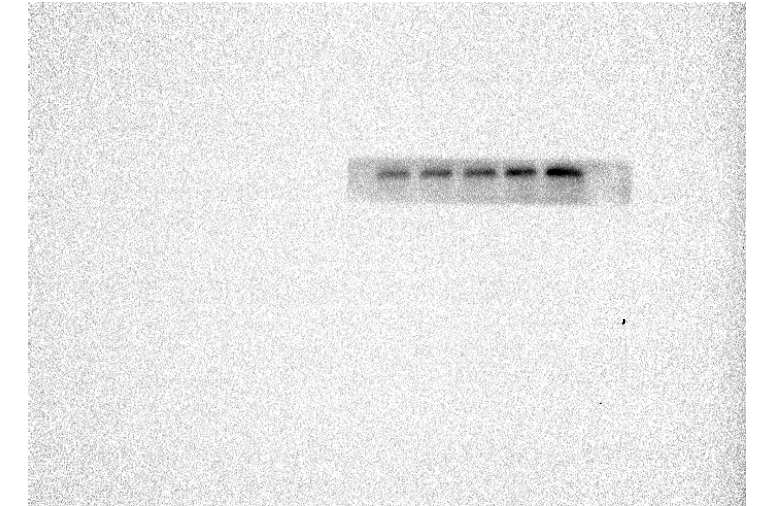

Supplement: Supplementary file 1 [file DataSheet1.ZIP › Raw Data/figure6/fig6. WB/Nrf2.jpg]

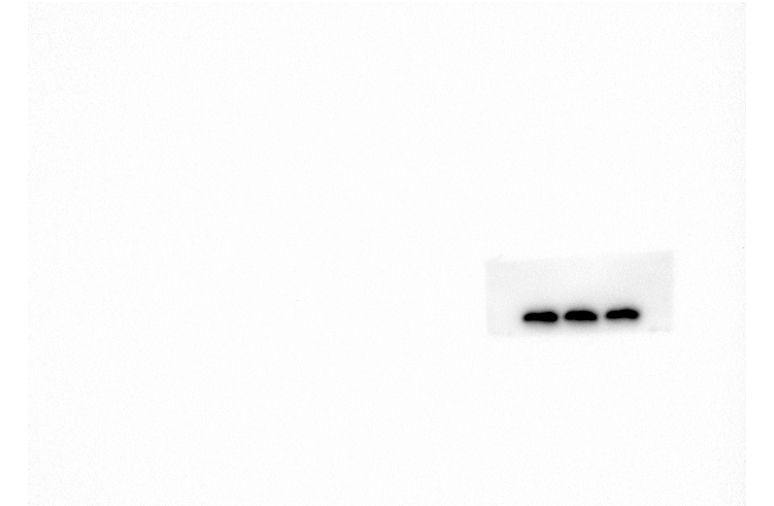

Supplement: Supplementary file 1 [file DataSheet1.ZIP › Raw Data/figure6/fig6. WB/β-actin.jpg]

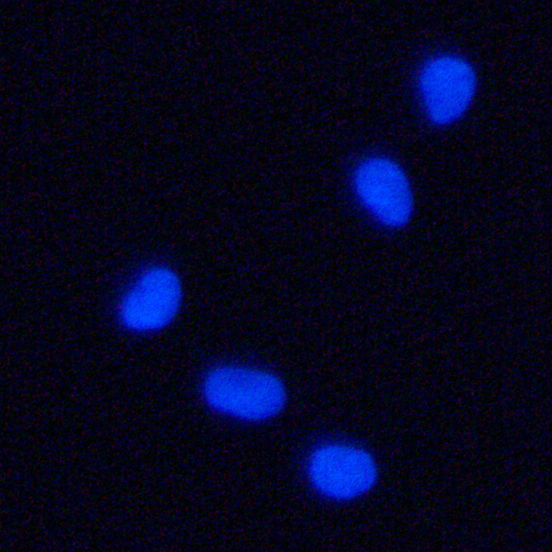

Supplement: Supplementary file 1 [file DataSheet1.ZIP › Raw Data/figure6/fig6.C/DAPI control.jpg]

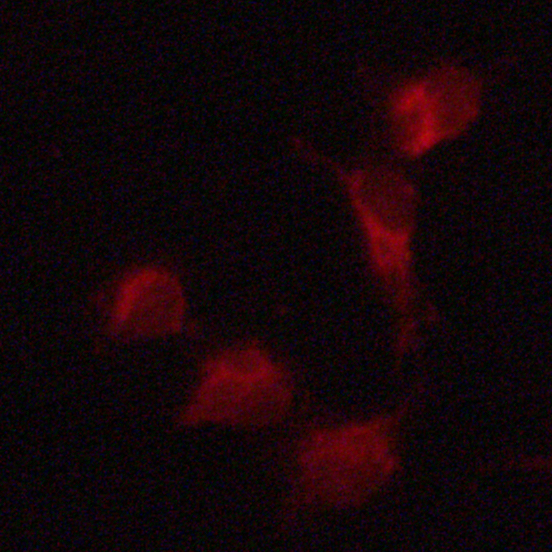

Supplement: Supplementary file 1 [file DataSheet1.ZIP › Raw Data/figure6/fig6.C/Nrf2 control.jpg]

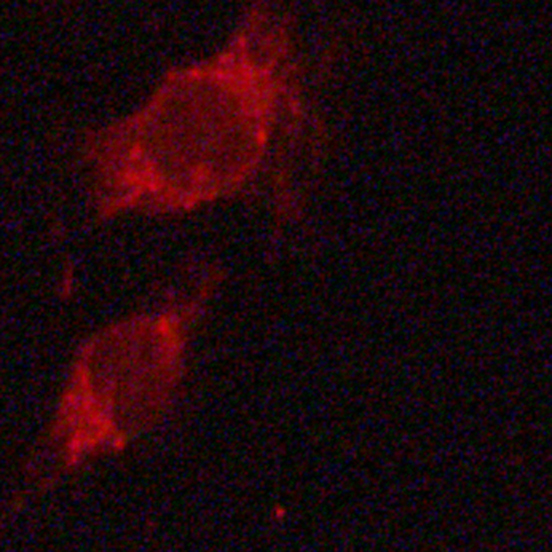

Supplement: Supplementary file 1 [file DataSheet1.ZIP › Raw Data/figure6/fig6.C/Nrf2 TBHP.jpg]

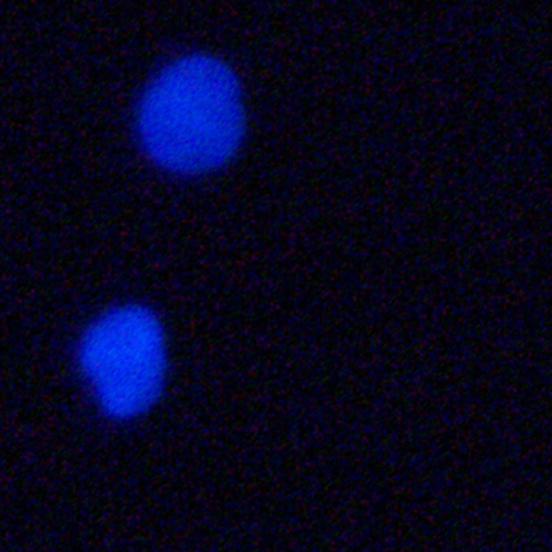

Supplement: Supplementary file 1 [file DataSheet1.ZIP › Raw Data/figure6/fig6.C/TBHP DAPI.jpg]

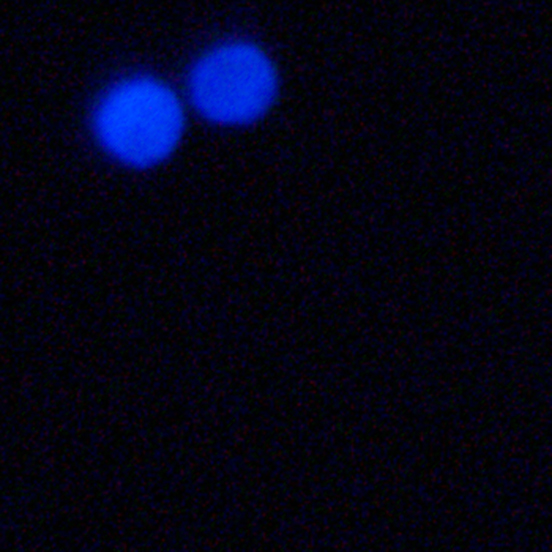

Supplement: Supplementary file 1 [file DataSheet1.ZIP › Raw Data/figure6/fig6.C/TBHP+PF DAPI.jpg]

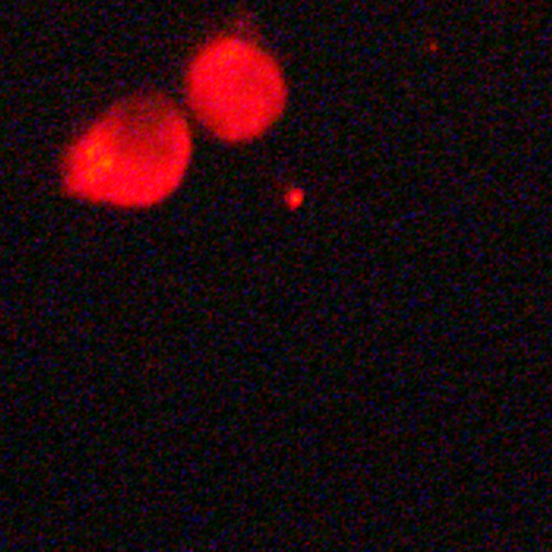

Supplement: Supplementary file 1 [file DataSheet1.ZIP › Raw Data/figure6/fig6.C/TBHP+PF.jpg]

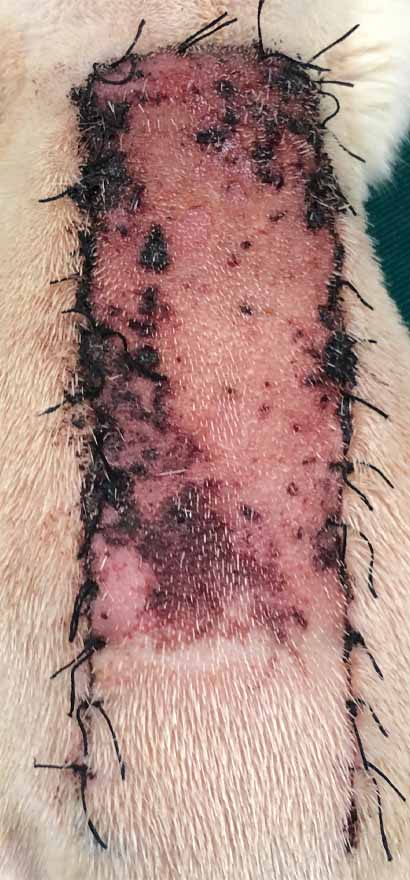

Supplement: Supplementary file 1 [file DataSheet1.ZIP › Raw Data/figure7/fig7. A/Day 7 ML385.jpg]

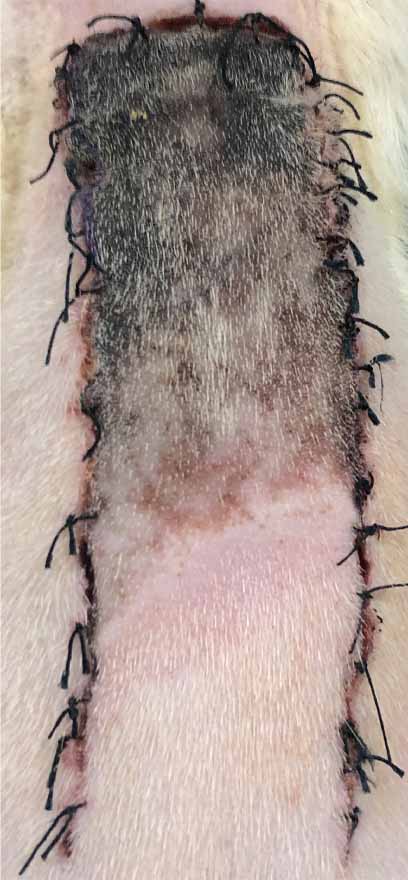

Supplement: Supplementary file 1 [file DataSheet1.ZIP › Raw Data/figure7/fig7. A/Day ML385.jpg]

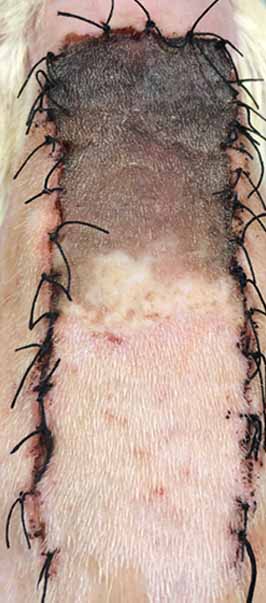

Supplement: Supplementary file 1 [file DataSheet1.ZIP › Raw Data/figure7/fig7. A/POD3 control.jpg]

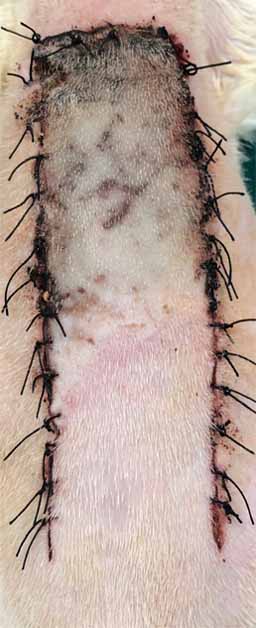

Supplement: Supplementary file 1 [file DataSheet1.ZIP › Raw Data/figure7/fig7. A/POD3 PF+ML385.jpg]

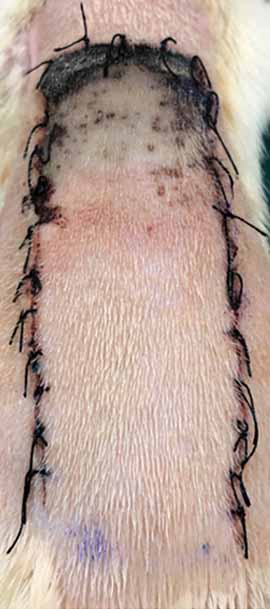

Supplement: Supplementary file 1 [file DataSheet1.ZIP › Raw Data/figure7/fig7. A/POD3 PF.jpg]

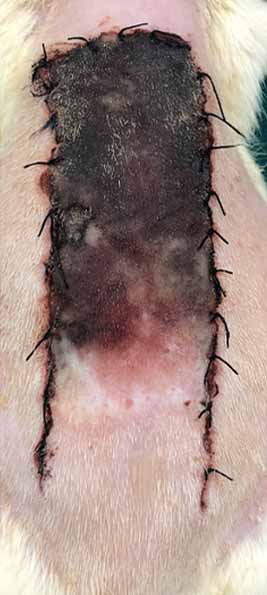

Supplement: Supplementary file 1 [file DataSheet1.ZIP › Raw Data/figure7/fig7. A/POD7 control.jpg]

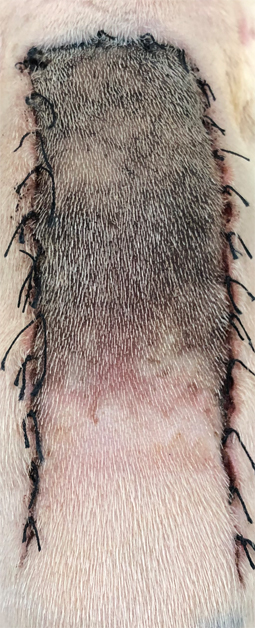

Supplement: Supplementary file 1 [file DataSheet1.ZIP › Raw Data/figure7/fig7. A/POD7 PF+ML385.jpg]

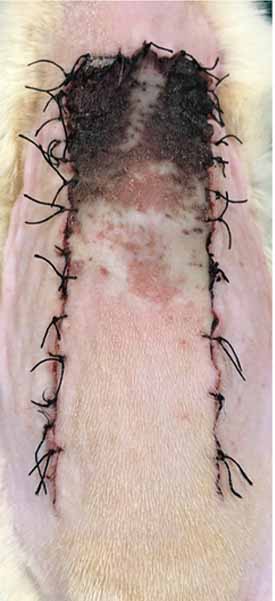

Supplement: Supplementary file 1 [file DataSheet1.ZIP › Raw Data/figure7/fig7. A/POD7 PF.jpg]

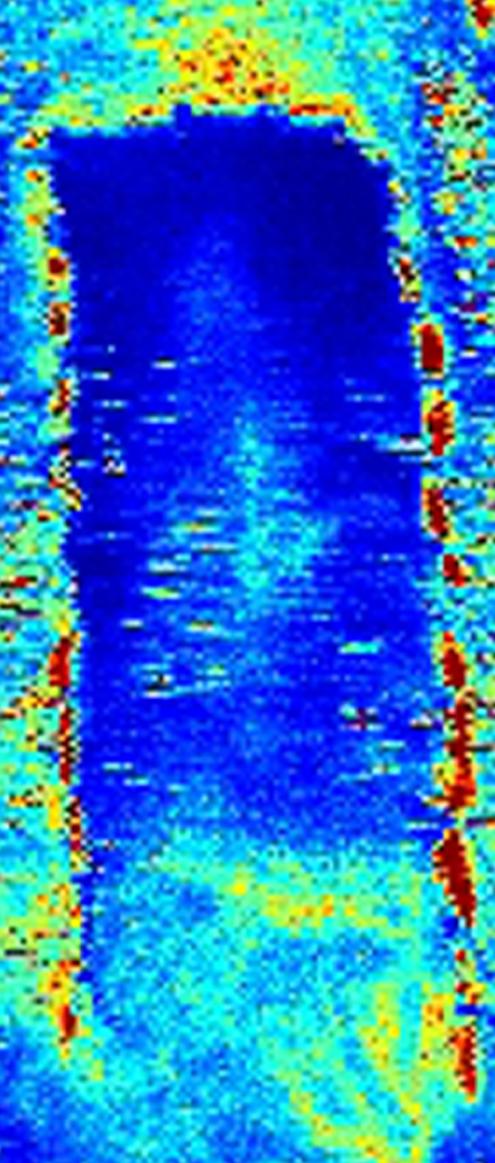

Supplement: Supplementary file 1 [file DataSheet1.ZIP › Raw Data/figure7/fig7. C/Control.jpg]

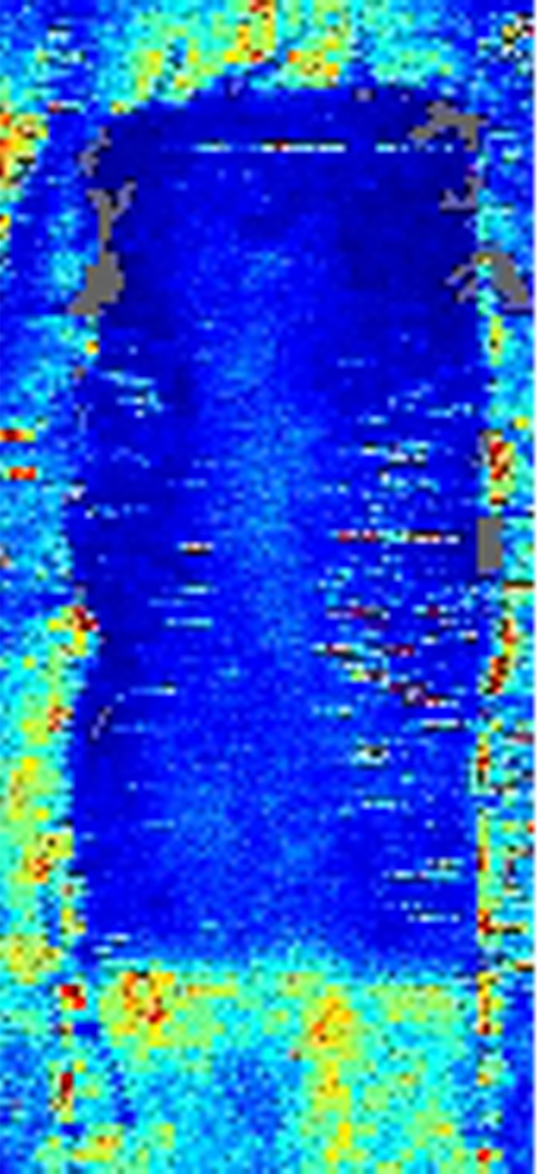

Supplement: Supplementary file 1 [file DataSheet1.ZIP › Raw Data/figure7/fig7. C/ML385.jpg]

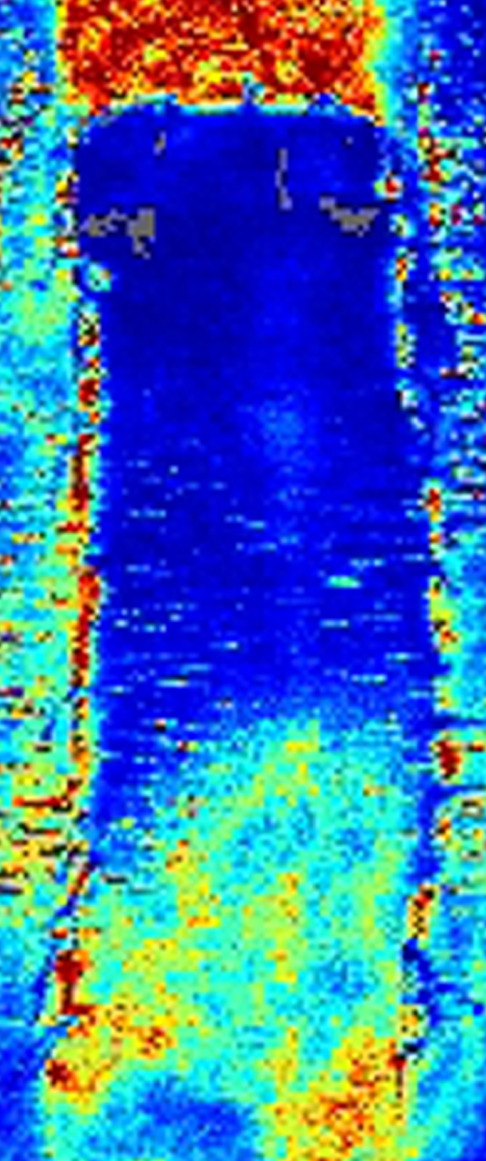

Supplement: Supplementary file 1 [file DataSheet1.ZIP › Raw Data/figure7/fig7. C/PF+ML385.jpg]

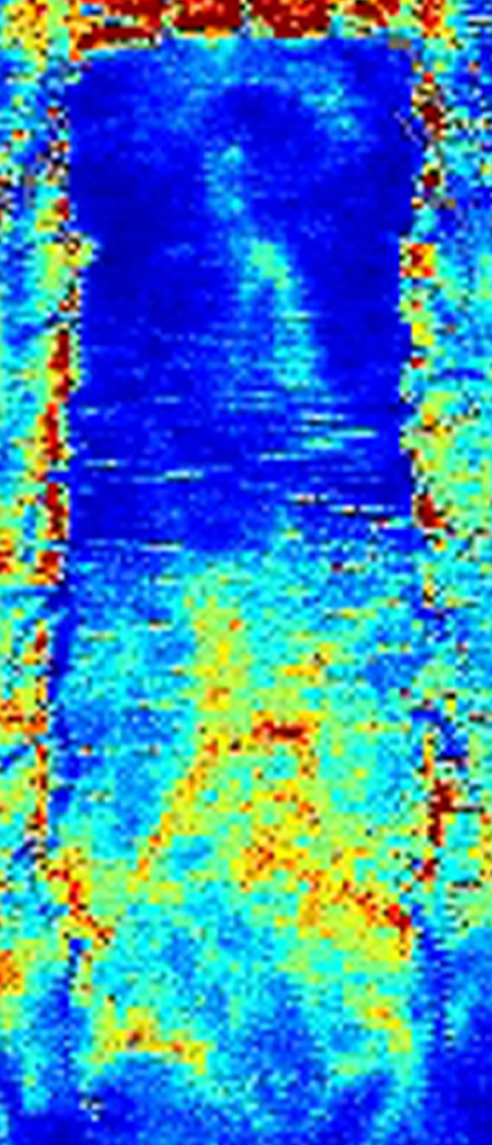

Supplement: Supplementary file 1 [file DataSheet1.ZIP › Raw Data/figure7/fig7. C/PF.jpg]

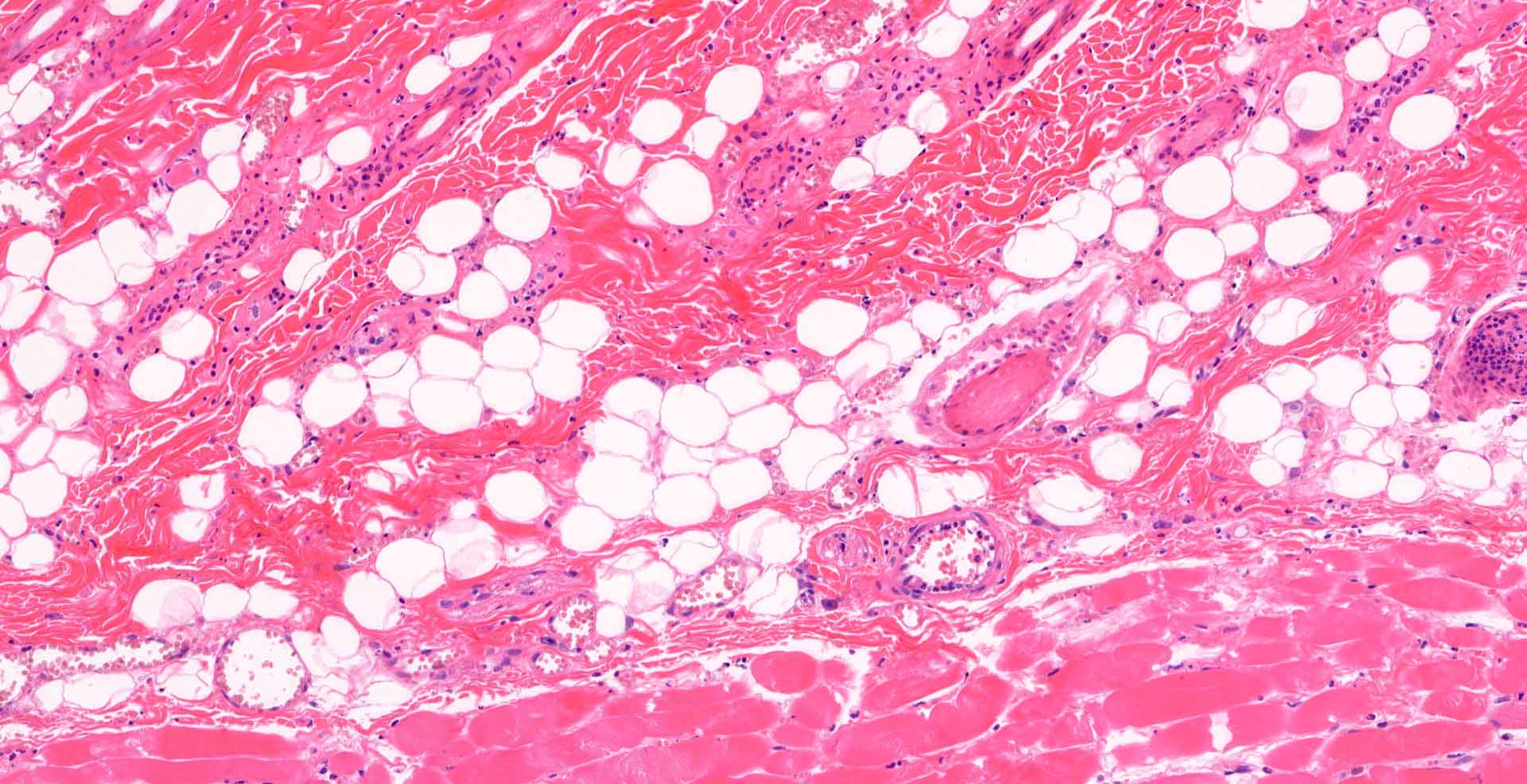

Supplement: Supplementary file 1 [file DataSheet1.ZIP › Raw Data/figure7/fig7. E/control.jpg]

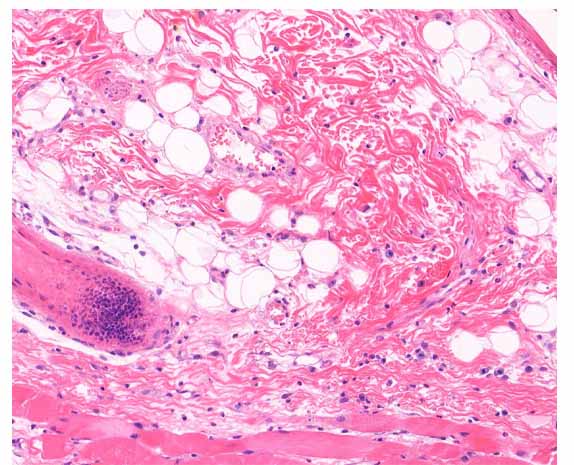

Supplement: Supplementary file 1 [file DataSheet1.ZIP › Raw Data/figure7/fig7. E/ML385.jpg]

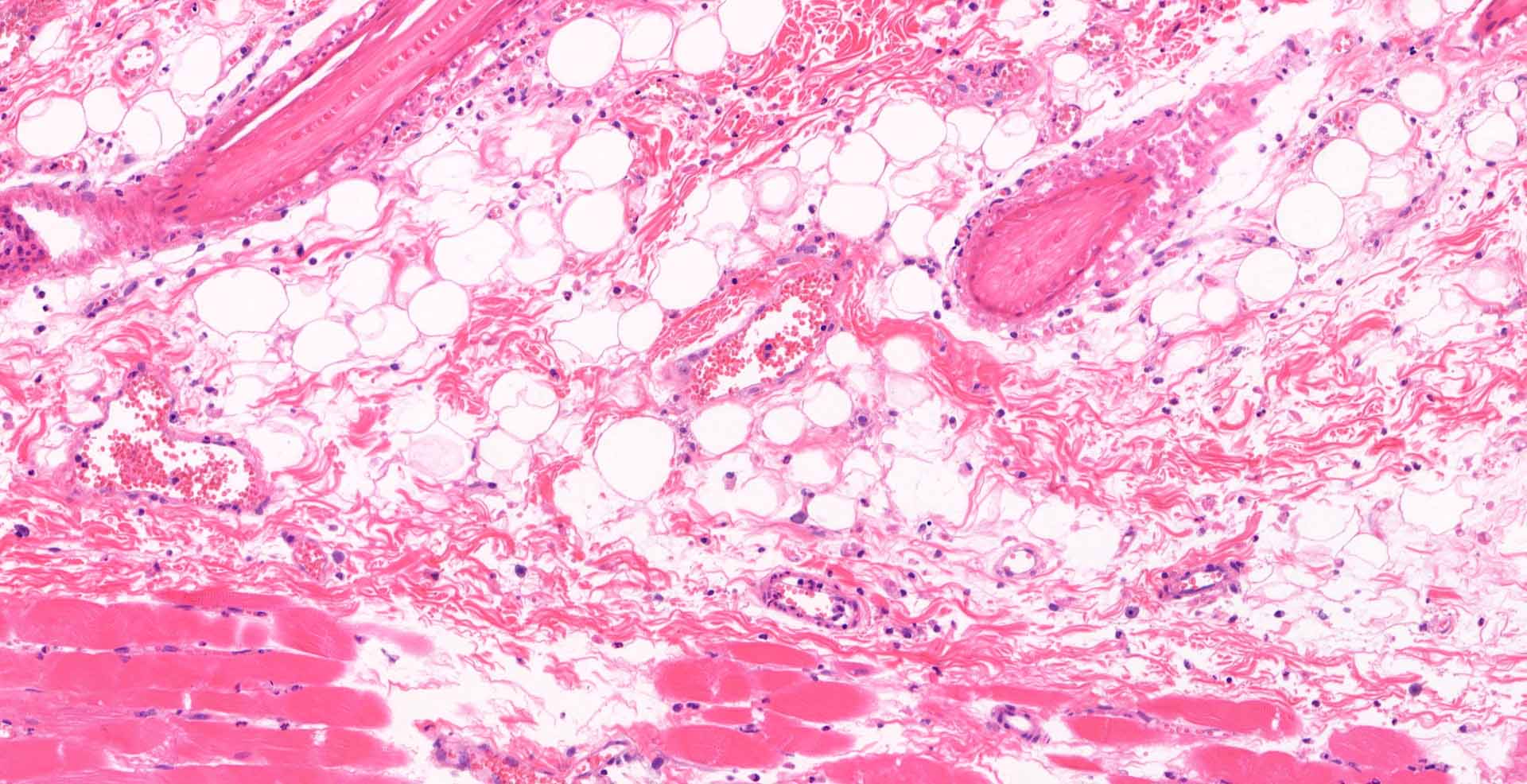

Supplement: Supplementary file 1 [file DataSheet1.ZIP › Raw Data/figure7/fig7. E/PF+ML385.jpg]
